# Relationship between screen-play scenarios' effectiveness and player classification in elite wheelchair basketball based on match results of Tokyo 2020 Paralympic Games

**DOI:** 10.3389/fspor.2024.1418130

**Published:** 2024-09-13

**Authors:** Taku Yasuda, Kaori Tachibana, Hirotaka Mutsuzaki

**Affiliations:** ^1^ Center for Medical Sciences, Ibaraki Prefectural University of Health Sciences, Ami, Japan; ^2^ Department of Physical Therapy, School of Healthcare, Ibaraki Prefectural University of Health Sciences, Ami, Japan

**Keywords:** tactical behavior, para-sports, performance analysis, sports performances, team sports

## Abstract

**Background:**

The competitiveness of wheelchair basketball has increased over time. However, screen-play, considered a vital offensive tactic in running basketball, is still poorly clarified. Therefore, this study aimed to clarify the impact of screen-play on scoring and game results in wheelchair basketball and assess the roles of each player classification (PC).

**Methods:**

Information regarding screen-play, including 13 categories such as shot success, location, and PC, was recorded for 22 wheelchair basketball games in the Tokyo 2020 Paralympic Games. This information was analyzed using the chi-square test to evaluate the significant differences in the appearance frequency of variables in each category (categorical variable) between the winning and losing teams and the shot-success rate.

**Results:**

Except for PC-related categorical variables, comparing the appearance frequency of the winning and losing teams confirmed a significant difference for screen and pass locations (all *p* < 0.05). Regarding the shot-success rates of the winning and losing teams, a significant difference in five categories was confirmed, including shot and pass locations (all *p* < 0.05). Regarding the PC, comparing the appearance frequency of the winning and losing teams confirmed a significant difference for PC of the screener (*p* < 0.05). Significant differences were found in the shot-success rates of the winning and losing teams in nine, five, three, and four categories regarding the PCs of the shooter, user, screener, and passer, respectively, such as shot location, pass location, and type of screen (*p* < 0.05, respectively).

**Conclusion:**

In wheelchair basketball offenses, it may be effective to consider the following points in the scenario lead-up to a shot: Using two different spaces, in the paint and the 3-point field goal area, could be crucial in screen-play. Improving the accuracy of on-the-ball screen plays appears vital, and using off-the-ball screens could also contribute to winning. Allocating approximately 50% of screeners to the middle-point classification (Middle) players and the rest to the low-point (Low) and high-point (High) classification players, at approximately 25% each, may be practical. Regarding winning team player roles, using High shooters and users; Low, Middle, and High screeners; and Middle and High passers contributed to play success.

## 1 Introduction

Wheelchair basketball is a sport originally used mainly as a treatment for war-injured soldiers and was adopted by the first Paralympic Games in Rome in 1960 (1). As international tournaments are flourishing, not only rehabilitation aspects but also competitive aspects are garnering more attention (2). To date, 108 national institutions have been affiliated with the International Wheelchair Basketball Federation (3). Wheelchair basketball is one of the world's top-rated sports featured in the Paralympics.

Wheelchair basketball is based on similar rules as running basketball; however, the use of wheelchairs and player classification (PC) simultaneously differentiates it from running basketball. PC involves eight categories, ranging from 1.0 to 4.5, with 0.5-point increments. The eight categories of PC are determined by the difference between the players' volume of action (the limit to which a player can move voluntarily in any direction and return to the upright seated position with control without holding the wheelchair for support or using the upper extremities to aid the movement) (4).

Some studies on wheelchair basketball have been conducted based on the players' performance in actual games, as described below. These studies examined differences between individual competitors, [categorized by PC] based on statistical data recorded in an official match, including the success or failure of shots, number of rebounds, assists, and turnovers. For example, Vanlandewijck et al. (5) analyzed the players' performance based on statistical data collected from the World Championship men's games in 1998. They reported that the PC represented the functional potential of the players. For instance, this study indicated that the 4.0- and 4.5-point players are significantly superior in rebounding and shooting close to the basket because of their seat height, maneuverability, power to close the basket, and ability to control their trunks for grasping the rebound. Additionally, Molik et al. (6) analyzed the quality of each player's contribution using statistical data of the game, such as scores, shots, and rebounds, based on their classification in the women's games, which tend to be lower scoring, at the World Championships in 2006. They clarified that this depended on the team's ranking, based on the order assigned in the tournament. However, these studies are based on individual perspectives. Since wheelchair basketball is a team sport, it is also essential to analyze performance from a team perspective. However, only a few studies have focused on teams, such as the study by Gómez et al. (7). The authors focused on the Beijing Paralympics in 2008 and the World Championships in 2010 and reported that the following factors that affected the match outcome: the field goal percentage and free throw rate (i.e., free-throws made/field-goals attempted) in men's games, and the field goal percentage and offensive rebound percentage [i.e., offensive rebounds/(offensive rebounds + opponents' defensive rebounds)] in women's games.

Furthermore, when focusing on the team, each player's performance in the game is exhibited in cooperation with other players by using tactics. Thus, clarifying the tactics of wheelchair basketball via statistical analyses of data is also necessary. Recently, Francis et al. (8) developed a model that predicted the outcome of field goal attempts based on specific action variables included in the categorical predictor variables. They analyzed five variables related to offensive tactics, including Shot Location and PC. “Pre shot” includes an offensive tactic that uses screens, such as “Curl” and “Pick & Roll”. These tactics have also been described as “vital to any type of offense” in running basketball (9). Moreover, because the wheelchair is considered a part of the player (10), has a constant width, and cannot move laterally without changing the wheels' direction, players cannot pass through narrow spaces as in running basketball. Therefore, in wheelchair basketball, “a player can neither jump nor move laterally,” screen-play is essential for the offense to break through the defense and shoot with a higher success rate (11). However, the abovementioned previous studies have not conducted a detailed analysis of screen-play.

In summary, while wheelchair basketball has been emphasized as a competitive sport, studies focusing on its competitiveness remain limited. Therefore, this study aimed to clarify the impact of screen-play on scoring and game results and assess the role of each PC in wheelchair basketball.

## 2 Materials and methods

### 2.1 Participants

This cross-sectional study targets multiple teams participating in a single international competition, the Tokyo 2020 Paralympics. We analyzed 22 games, where the top 8 of the 12 men's teams participated in wheelchair basketball; each team consisted of 12 players.

The need for written informed consent was waived by the Institutional Review Board of Ibaraki Prefectural University of Health Sciences, since this research has a retrospective nature, did not include any identifying data, and only used the data of the games that are open to the public. As an alternative to written informed consent, we announced an information disclosure document regarding ethical considerations on the Ibaraki Prefectural University of Health Sciences Hospital website. The Institutional Review Board of Ibaraki Prefectural University of Health Sciences approved the above method. This study was conducted in accordance with the Declaration of Helsinki and was approved by the Institutional Review Board of Ibaraki Prefectural University of Health Sciences (No. e390).

### 2.2 Study procedures

We recorded all 3,841 possessions in 22 games and considered 2,567 offensive sequences that resulted in field goal attempts for analysis. All data generated and analyzed during this study were obtained from the footage of those games on the Paralympics YouTube channel (https://www.youtube.com/@paralympics).

#### 2.2.1 Categorization of screen-play

Information regarding the following 13 categories was recorded and screen plays that led to field goal attempts in each game were analyzed: (Ⅰ) the success of shots (made/missed), (Ⅱ) presence of a screen (with/without), (Ⅲ) location of shots on screen plays (shot location), (Ⅳ) location of the screen used immediately before shots (screen location), (Ⅴ) location of the pass issued immediately before a shot on screen plays (pass location), (Ⅵ) type of screen, (Ⅶ) type of screen-play, (Ⅷ) movement of on-the-ball screen plays, (Ⅸ) movement of off-the-ball screen plays, (X) PC of the shooter on screen plays, (Ⅺ) PC of the user, (Ⅻ) PC of the screener, and (XIII) PC of the passer who passed the ball to the shooter on screen plays.

**Figure 1:**
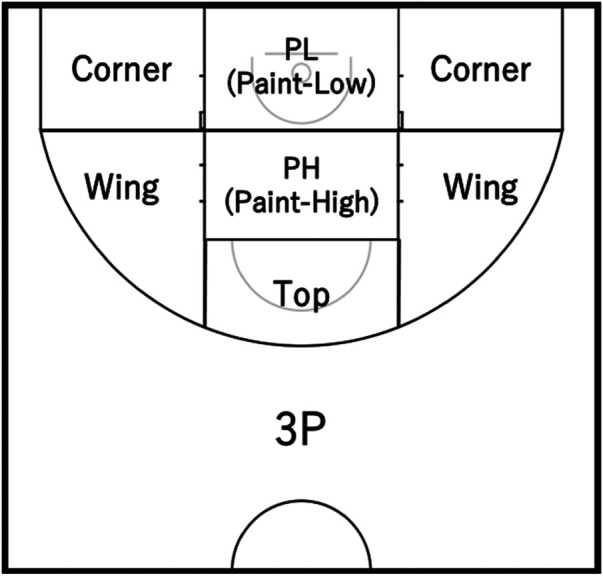
Shot/screen/pass locations.

Regarding (Ⅲ), (Ⅳ), and (Ⅴ), we divided each location into the following six areas based on the figure used by Francis et al. (8) (Figure 1): paint-low (PL), paint-high (PH), Top, Corner, Wing, 3-point field goal area (3P). Compared to the figure of Francis et al., our figure has the following three modifications: we divided the restricted area into two sections, considering that basketball tactics distinguish between the low-post and high-post (9); we also divided the 2-point field goal area into two sections, considering that basketball tactics distinguish between the corner and wing (9); as the base point for dividing those areas, we used the center line side edge of the neutral zone, which is on a line extending from the outer edge of the free-throw line toward the end-line (10).

Regarding (Ⅵ), we categorized the type of screen into the following two types: i) on-the-ball screens, where the user or screener held the ball; ii) off-the-ball screens, where neither the user nor the screener held the ball.

**Figure 2:**
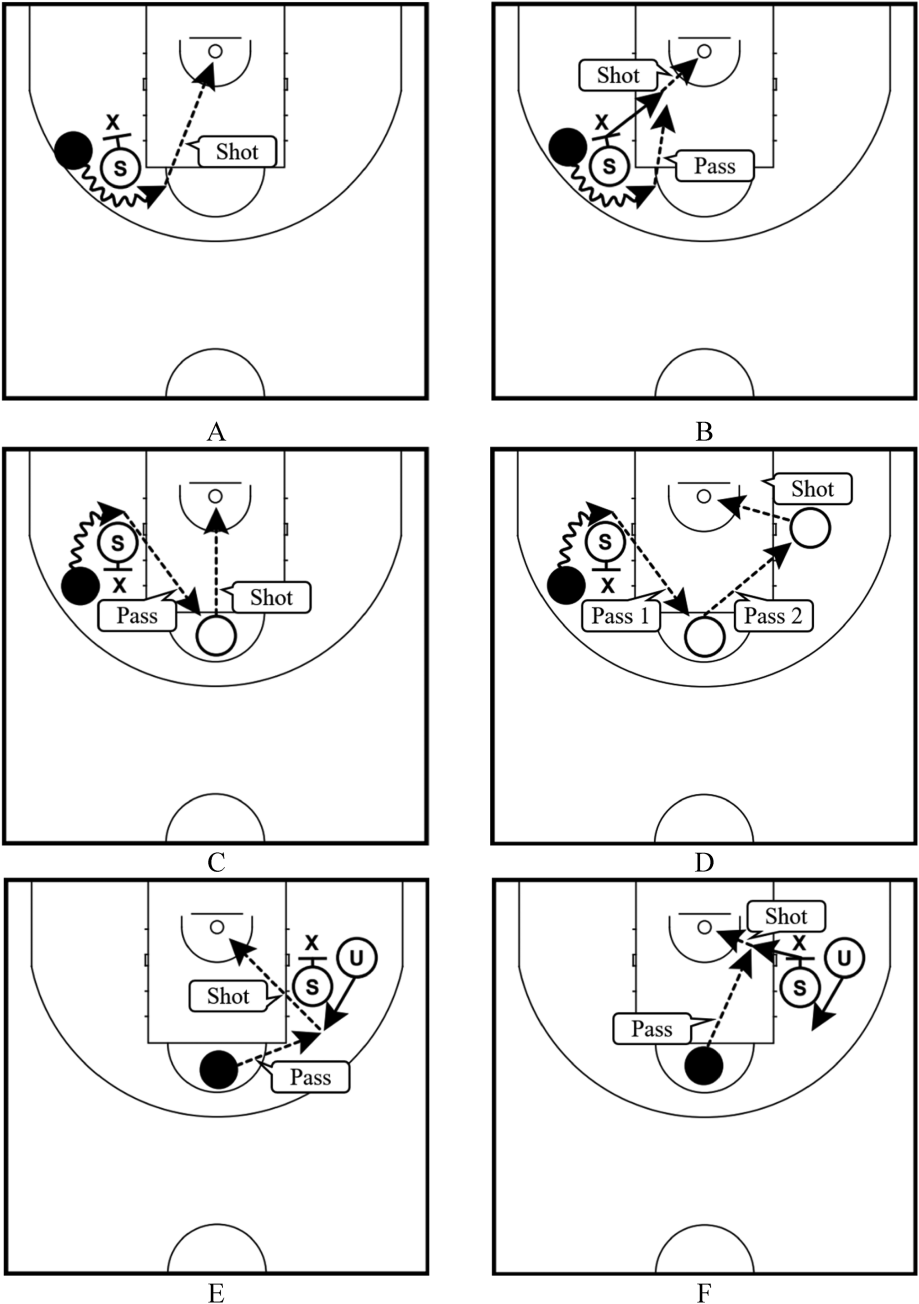
Types of screen-play; **(A)** the plays where the user shot using the on-the-ball screen (ON-U), **(B)** plays where the screener of the on-the-ball screen shot after receiving a pass from the user (ON-S), **(C)** plays where another player shot after receiving a pass from the user of the on-the-ball screen (ON-A), **(D)** plays that led to a shot through two or more extra passes after the user used the on-the-ball screen (ON-E), **(E)** plays where the user shot using the off-the-ball screen (OF-U), and **(F)** plays where the screener of the off-the-ball screen shot (OF-S).

Regarding (Ⅶ), we categorized the type of screen-play into the following six types based on the difference in the shooter (whether the shooter was the user, the screener, or another player of the screen play) and the process leading to the shot along with the type of screen mentioned above (9) (Figure 2): the plays where the user shot using the on-the-ball screen (ON-U); plays where the screener of the on-the-ball screen shot after receiving a pass from the user (ON-S); plays where another player shot after receiving a pass from the user of the on-the-ball screen (ON-A); plays that led to a shot through two or more extra passes after the user used the on-the-ball screen (ON-E); plays where the user shot using the off-the-ball screen (OF-U); and plays where the screener of the off-the-ball screen shot (OF-S). In wheelchair basketball, a player may progress with a live ball on the court in any direction unless the number of pushes while holding the ball exceeds 2 (10). Therefore, we categorized ON-U when it was confirmed that the user pushed the big wheel without dribbling after holding the ball by using the off-the-ball screen and OF-U when both dribbling and pushing the big wheel were not confirmed.

**Figure 3:**
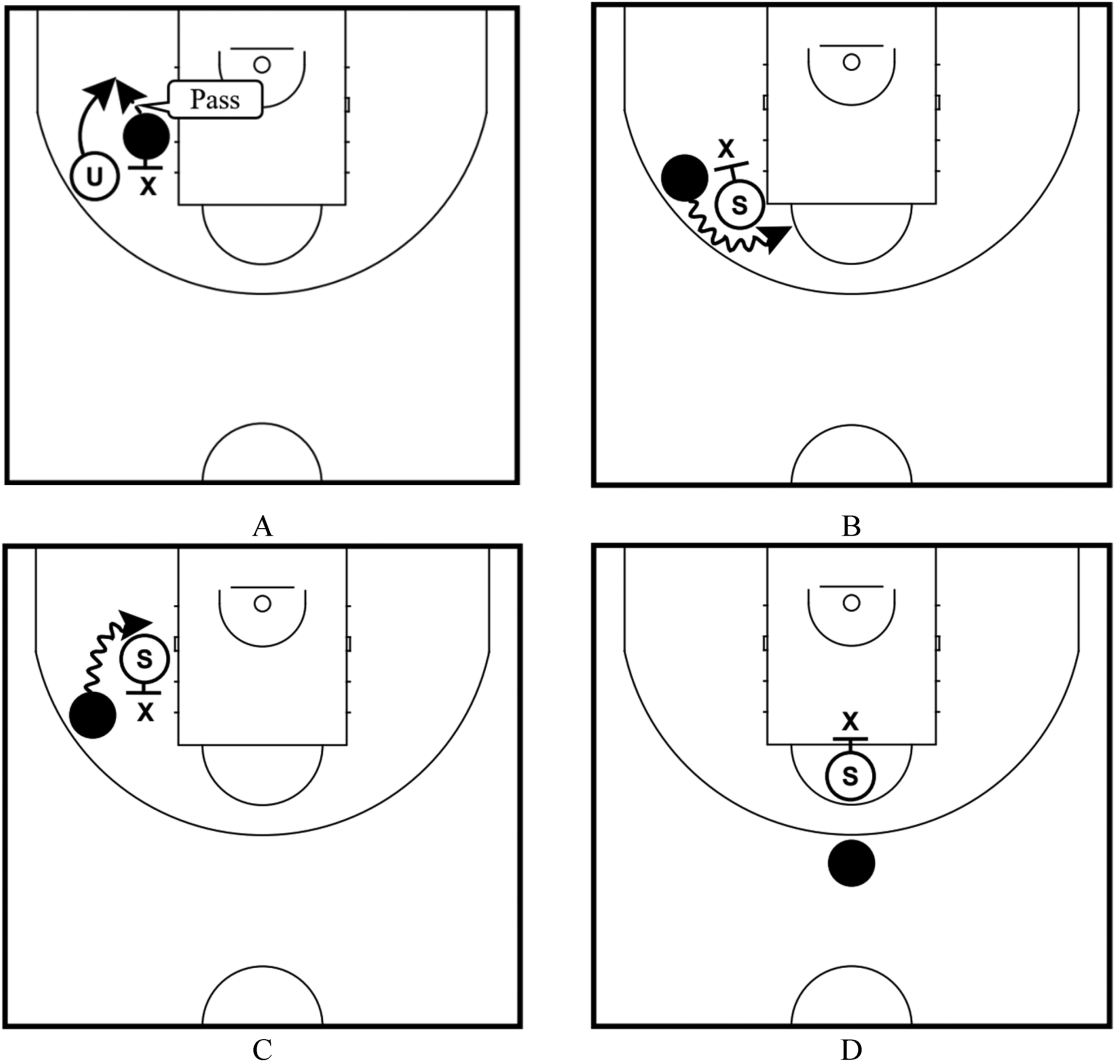
Movements of on-the-ball screen plays; **(A)** the plays where the screener held a ball (Around), **(B)** plays where the user moved toward the center-line side against the screener (Center-line), **(C)** plays where the user moved toward the end-line side against the screener (End-line), and **(D)** plays where the screener was on the center-line side of the defense who protected the user holding a ball in a Top or 3P on Top extension (ON-Down).

Regarding (Ⅷ), we categorized the movement of on-the-ball screen plays into four variables based on the difference in movements of the user after using the on-the-ball screen (9) and whether the screener held the ball or not (12) (Figure 3).

**Figure 4:**
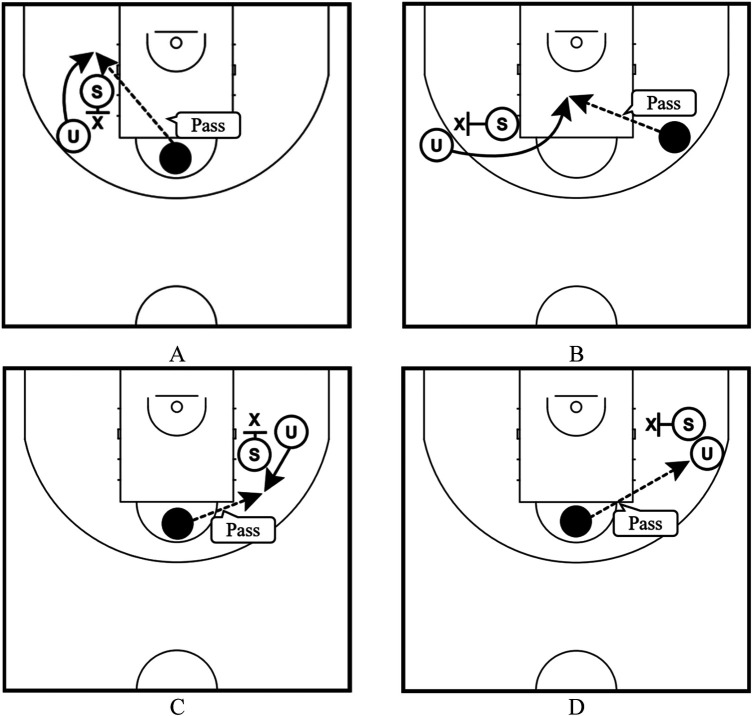
Movements of off-the-ball screen plays; **(A)** the plays where the screener was on the end-line side of the defense who protected the user (Back), **(B)** the plays where the screener was on the middle-line (the imaginary line connecting baskets running through the center of the court) side of the defense who protected the user (Cross), **(C)** the plays where the screener was on the center-line side of the defense who protected the user (Down), and **(D)** the plays where the screener was on the side-line side of the defense who protected the user (Flare).

Regarding (Ⅸ), we categorized the movement of off-the-ball screen plays into four variables according to the location of the screener relative to the defense, considering the screen's angle (9) (Figure 4).

Regarding (X), (Ⅺ), (Ⅻ), and (XIII), referring to the study by Francis et al. (8), we set a PC of 1.0 and 1.5, 2.0–3.0, and 3.5–4.5 as low-point (Low), middle-point (Middle), and high-point (High) category players, respectively, based on the PC recognized by the classifiers.

#### 2.2.2 Data extraction

A single observer extracted data from the videos according to the abovementioned categories. The observer, who had more than 10 years of experience as a basketball coach and a master's degree in physical education, received suggestions on wheelchair basketball tactics and confirmation of screen-play categorization validity in this study from a former national team coach in wheelchair basketball.

The conditions set for data extraction are described in the following paragraphs. These criteria were set to guarantee the reproducibility of the results and control the conditions for checking screen plays.

Firstly, in wheelchair basketball, screen plays both in the frontcourt and backcourt are effective tactics because players cannot move laterally (13, 14). However, the effectiveness of screen plays in the frontcourt and backcourt may differ. In running basketball, screen plays are mainly described assuming they will occur primarily in the frontcourt (9, 12, 15, 16). In wheelchair basketball, there is an offensive tactic called a “back pick” that uses a screen in the backcourt (13, 17), but similar to running basketball, there is a tendency to mainly describe screen plays in the frontcourt (8, 14). This is because screen plays in the frontcourt are thought to be more directly linked to scoring than in the backcourt, since the defense has less space and time to deal with screen plays in the frontcourt. Therefore, we only analyzed screen plays occurring in the frontcourt.

Secondly, this study aimed to assess the impact of screen-play, which corresponded to 2-on-2 among offensive tactics. Therefore, we excluded the following five types of plays from the screen plays to avoid including plays that do not fit the 2-on-2 screen-play criteria and to increase reproducibility regarding data extraction: (i) plays where the user who held the ball used the screen and stopped dribbling and then dribbled again to shoot, (ii) plays where the user with the ball turned his back to the basket immediately after using a screen, and (iii) plays where the user with the ball faked immediately after using a screen and then dribbled and passed. Furthermore, we excluded (iv) plays with <14 s remaining on the shot clock at the start of offense and (v) plays that led to a direct shot from a throw-in pass at the side or end-line. In running basketball, it has been reported that players are likely to choose certain plays when the duration of the on-the-ball screen play is short (18). Thus, even in wheelchair basketball, situations (iv) and (v) could increase the possibility of using only certain types of screen-play or none at all.

Lastly, regarding the PC of the screener, screen location, and type of screen-play, we analyzed single screen plays (one screener); however, we excluded double screen plays (two screeners) since these occurred less frequently. Additionally, regarding the movement of on-the-ball screen plays, we excluded one play that did not fall into the above four types. Regarding the movement of off-the-ball screen plays, we excluded one play with different movement combinations by a double screen.

### 2.3 Statistical analysis

The extracted data were analyzed using the chi-square test to confirm whether there was a significant difference in the appearance frequency of each categorical variable and the shot-success rate (ratio of successful shots to the number of attempted shots) between the winning and losing teams. Additionally, to analyze the characteristics for the classification, the chi-square test was used to confirm the difference in shot-success rate for the other variables associated with each category of the PC: the PC of the shooter, user, screener, and passer.

All statistical analyses were performed using IBM SPSS Statistics for Windows version 28 (IBM Corp., Armonk, NY, USA), and all significance levels were set at *p* < 0.05. Furthermore, referring to the studies by Agresti (19) and Sharpe (20), in cases where a significant difference was confirmed among three or more groups, we used adjusted standardized residual (ASR) analysis to interpret the data. In this study, ASR > +2 indicated a higher frequency than the compared target, and ASR < −2 indicated a lower frequency than the compared target. In addition, as based on the study by Fritz et al. (21) regarding effect sizes, we used *φ* where the data was analyzed in 2 × 2 contingency tables and used Cramér's V (*φ*_c_) for larger contingency tables than 2 × 2. The formulas for *φ* and *φ*_c_ are as follows, where N is the sample size and k is the number of independent variables in the analysis:

$$\varphi =\sqrt{\frac{\chi^{2}}{N}}$$

$$\varphi_{c}=\sqrt{\frac{\chi^{2}}{N(k-1)}}$$

## 3 Results

Given the extensive and comprehensive analyses conducted in this study, detailed statistical information is provided exclusively for categories where significant differences were observed (Tables 1–7).

**Table 1:** Differences in the appearance frequency of each categorical variable in screen-play.

Screen location		Win	Lose	χ^2^ (df = 5)	*p*	Cramér's V (*φ*_c_)
PL	Frequency	53	55	22.483*	<0.001	0.112
	Percentage	5.8%	6.3%			
	ASR	−0.486	0.486			
PH	Frequency	89	58			
	Percentage	9.7%	6.7%			
	ASR	2.330	−2.330			
Top	Frequency	70	113			
	Percentage	7.6%	13.0%			
	ASR	−3.739	3.739			
Corner	Frequency	260	228			
	Percentage	28.3%	26.2%			
	ASR	1.004	−1.004			
Wing	Frequency	395	349			
	Percentage	43.0%	40.1%			
	ASR	1.250	−1.250			
3P	Frequency	52	68			
	Percentage	5.7%	7.8%			
	ASR	−1.817	1.817			
Pass location		Win	Lose	χ^2^ (df = 5)	*p*	Cramér's V (*φ*_c_)
PL	Frequency	22	12	14.242*	0.014	0.111
	Percentage	3.8%	2.1%			
	ASR	1.710	−1.710			
PH	Frequency	85	68			
	Percentage	14.6%	11.8%			
	ASR	1.407	−1.407			
Top	Frequency	95	112			
	Percentage	16.3%	19.4%			
	ASR	−1.386	1.386			
Corner	Frequency	130	98			
	Percentage	22.3%	17.0%			
	ASR	2.278	−2.278			
Wing	Frequency	159	169			
	Percentage	27.3%	29.3%			
	ASR	−0.763	0.763			
3P	Frequency	91	117			
	Percentage	15.6%	20.3%			
	ASR	−2.073	2.073			

PL, paint-low; PH, paint-high; 3P, 3-point field goal area; ASR, adjusted standardized residual.

* *p* < 0.05.

**Table 2:** Differences in shot-success rate of each categorical variable in screen-play.

Presence of screen			Success	Fail	χ^2^ (df = 1)	*p*	*φ*
With screen	Win	Frequency	413	523	4.469*	0.035	0.050
		Percentage	44.1%	55.9%			
	Lose	Frequency	344	533			
		Percentage	39.2%	60.8%			
Without screen	Win	Frequency	203	172	3.066	0.080	0.064
		Percentage	54.1%	45.9%			
	Lose	Frequency	181	198			
		Percentage	47.8%	52.2%			
Shot location			Success	Fail	χ^2^ (df = 1)	*p*	*φ*
PL	Win	Frequency	142	107	0.046	0.083	0.010
		Percentage	57.0%	43.0%			
	Lose	Frequency	134	105			
		Percentage	56.1%	43.9%			
PH	Win	Frequency	37	37	8.902*	0.003	0.241
		Percentage	50.0%	50.0%			
	Lose	Frequency	21	58			
		Percentage	26.6%	73.4%			
Top	Win	Frequency	35	56	0.162	0.687	−0.031
		Percentage	38.5%	61.5%			
	Lose	Frequency	34	48			
		Percentage	41.5%	58.5%			
Corner	Win	Frequency	90	112	0.026	0.872	0.008
		Percentage	44.6%	55.4%			
	Lose	Frequency	84	108			
		Percentage	43.8%	56.3%			
Wing	Win	Frequency	70	126	1.588	0.208	0.068
		Percentage	35.7%	64.3%			
	Lose	Frequency	43	104			
		Percentage	29.3%	70.7%			
3P	Win	Frequency	39	85	4.275*	0.039	0.128
		Percentage	31.5%	68.5%			
	Lose	Frequency	28	110			
		Percentage	20.3%	79.7%			
Pass location			Success	Fail	χ^2^ (df = 1)	*p*	*φ*
PL	Win	Frequency	11	11	0.000	1.000	0.010
		Percentage	50.0%	50.0%			
	Lose	Frequency	6	6			
		Percentage	50.0%	50.0%			
PH	Win	Frequency	41	44	1.535	0.215	0.100
		Percentage	48.2%	51.8%			
	Lose	Frequency	26	42			
		Percentage	38.2%	61.8%			
Top	Win	Frequency	39	56	2.378	0.123	−0.107
		Percentage	41.1%	58.9%			
	Lose	Frequency	58	54			
		Percentage	51.8%	48.2%			
Corner	Win	Frequency	55	75	0.056	0.813	−0.016
		Percentage	42.3%	57.7%			
	Lose	Frequency	43	55			
		Percentage	43.9%	56.1%			
Wing	Win	Frequency	69	90	0.082	0.774	−0.016
		Percentage	43.4%	56.6%			
	Lose	Frequency	76	93			
		Percentage	45.0%	55.0%			
3P	Win	Frequency	47	44	7.080*	0.008	0.184
		Percentage	51.6%	48.4%			
	Lose	Frequency	39	78			
		Percentage	33.3%	66.7%			
Type of screen-play			Success	Fail	χ^2^ (df = 1)	*p*	*φ*
ON-U	Win	Frequency	154	206	7.209*	0.007	0.104
		Percentage	42.8%	57.2%			
	Lose	Frequency	102	210			
		Percentage	32.7%	67.3%			
ON-S	Win	Frequency	31	38	2.075	0.150	−0.122
		Percentage	44.9%	55.1%			
	Lose	Frequency	40	30			
		Percentage	57.1%	42.9%			
ON-A	Win	Frequency	74	104	0.726	0.394	0.045
		Percentage	41.6%	58.4%			
	Lose	Frequency	65	110			
		Percentage	37.1%	62.9%			
ON-E	Win	Frequency	33	52	1.396	0.237	−0.090
		Percentage	38.8%	61.2%			
	Lose	Frequency	42	46			
		Percentage	47.7%	52.3%			
OF-U	Win	Frequency	108	118	2.312	0.128	0.073
		Percentage	47.8%	52.2%			
	Lose	Frequency	86	126			
		Percentage	40.6%	59.4%			
OF-S	Win	Frequency	13	5	2.880	0.090	0.275
		Percentage	72.2%	27.8%			
	Lose	Frequency	9	11			
		Percentage	45.0%	55.0%			
Movement of off-the-ball screen plays			Success	Fail	χ^2^ (df = 1)	*p*	*φ*
Back	Win	Frequency	29	23	3.801	0.051	0.185
		Percentage	55.8%	44.2%			
	Lose	Frequency	22	37			
		Percentage	37.3%	62.7%			
Cross	Win	Frequency	17	8	0.266	0.606	0.074
		Percentage	68.0%	32.0%			
	Lose	Frequency	14	9			
		Percentage	60.9%	39.1%			
Down	Win	Frequency	27	27	5.302*	0.021	0.220
		Percentage	50.0%	50.0%			
	Lose	Frequency	16	40			
		Percentage	28.6%	71.4%			
Flare	Win	Frequency	47	65	0.297	0.586	−0.038
		Percentage	42.0%	58.0%			
	Lose	Frequency	43	51			
		Percentage	45.7%	54.3%			

PL, paint-low; PH, paint-high; 3P, 3-point field goal area; ON-U, the plays where the user shot using the on-the-ball screen; ON-S, plays where the screener of the on-the-ball screen shot after receiving a pass from the user; ON-A, plays where another player shot after receiving a pass from the user of the on-the-ball screen; ON-E, plays that led to a shot through two or more extra passes after the user used the on-the-ball screen; OF-U, plays where the user shot using the off-the-ball screen; OF-S, plays where the screener of the off-the-ball screen shot; Back, plays where the screener was on the end-line side of the defense who protected the user; Cross, plays where the screener was on the middle-line (the imaginary line connecting baskets running through the center of the court) side of the defense who protected the user; Down, plays where the screener on the center-line side of the defense who protected the user; Flare, plays where the screener on the side-line side of the defense who protected the user.

* *p* < 0.05.

**Table 3:** Differences in the appearance frequency in screen-play depending on the PC.

Screener		Win	Lose	χ^2^ (df = 5)	*p*	Cramér's V (*φ*_c_)
Low	Frequency	237	284	10.546*	0.005	0.077
	Percentage	25.8%	32.6%			
	ASR	−3.174	3.174			
Middle	Frequency	457	404			
	Percentage	49.7%	46.4%			
	ASR	1.416	−1.416			
High	Frequency	225	183			
	Percentage	24.5%	21.0%			
	ASR	1.751	−1.751			

PC, player classification; ASR, adjusted standardized residual.

* *p* < 0.05.

**Table 4:** Differences in shot-success rate for each categorical variable depending on the PC of the shooter.

Shooter	Presence of screen			Success	Fail	χ^2^ (df = 1)	*p*	*φ*
Low	With screen	Win	Frequency	24	36	0.906	0.341	−0.081
			Percentage	40.0%	60.0%			
		Lose	Frequency	38	41			
			Percentage	48.1%	51.9%			
Middle	With screen	Win	Frequency	184	256	0.446	0.504	0.023
			Percentage	41.8%	58.2%			
		Lose	Frequency	157	240			
			Percentage	39.5%	60.5%			
High	With screen	Win	Frequency	205	231	8.323*	0.004	0.100
			Percentage	47.0%	53.0%			
		Lose	Frequency	149	252			
			Percentage	37.2%	62.8%			

Shooter	Shot location			Success	Fail	χ^2^ (df = 1)	*p*	*φ*
Low	PL	Win	Frequency	10	11	0.707	0.400	−0.116
			Percentage	47.6%	52.4%			
		Lose	Frequency	19	13			
			Percentage	59.4%	40.6%			
Middle	PL	Win	Frequency	65	59	0.002	0.968	−0.003
			Percentage	52.4%	47.6%			
		Lose	Frequency	69	62			
			Percentage	52.7%	47.3%			
High	PL	Win	Frequency	67	37	0.285	0.593	0.040
			Percentage	64.4%	35.6%			
		Lose	Frequency	46	30			
			Percentage	60.5%	39.5%			
Low	PH	Win	Frequency	0	1			
			Percentage	0.0%	100.0%			
		Lose	Frequency	0	2			
			Percentage	0.0%	100.0%			
Middle	PH	Win	Frequency	13	15	1.054	0.305	0.124
			Percentage	46.4%	53.6%			
		Lose	Frequency	14	27			
			Percentage	34.1%	65.9%			
High	PH	Win	Frequency	24	21	9.723*	0.002	0.346
			Percentage	53.3%	46.7%			
		Lose	Frequency	7	29			
			Percentage	19.4%	80.6%			
Low	Top	Win	Frequency	8	10	0.000	1.000	0.000
			Percentage	44.4%	55.6%			
		Lose	Frequency	12	15			
			Percentage	44.4%	55.6%			
Middle	Top	Win	Frequency	19	28	0.009	0.926	0.010
			Percentage	40.4%	59.6%			
		Lose	Frequency	13	20			
			Percentage	39.4%	60.6%			
High	Top	Win	Frequency	8	18	0.536	0.464	−0.106
			Percentage	30.8%	69.2%			
		Lose	Frequency	9	13			
			Percentage	40.9%	59.1%			
Low	Corner	Win	Frequency	6	9	0.056	0.812	0.044
			Percentage	40.0%	60.0%			
		Lose	Frequency	5	9			
			Percentage	35.7%	64.3%			
Middle	Corner	Win	Frequency	37	56	0.304	0.581	−0.042
			Percentage	39.8%	60.2%			
		Lose	Frequency	36	46			
			Percentage	43.9%	56.1%			
High	Corner	Win	Frequency	47	47	0.517	0.472	0.052
			Percentage	50.0%	50.0%			
		Lose	Frequency	43	53			
			Percentage	44.8%	55.2%			
Low	Wing	Win	Frequency	0	4		0.429 (**)	−0.577
			Percentage	0.0%	100.0%			
		Lose	Frequency	2	2			
			Percentage	50.0%	50.0%			
Middle	Wing	Win	Frequency	34	59	2.542	0.111	0.130
			Percentage	36.6%	63.4%			
		Lose	Frequency	14	44			
			Percentage	24.1%	75.9%			
High	Wing	Win	Frequency	36	63	0.430	0.512	0.048
			Percentage	36.4%	63.6%			
		Lose	Frequency	27	58			
			Percentage	31.8%	68.2%			
Low	3P	Win	Frequency	0	1			
			Percentage	0.0%	100.0%			
		Lose	Frequency	0	1			
			Percentage	0.0%	100.0%			
Middle	3P	Win	Frequency	16	39	0.893	0.345	0.091
			Percentage	29.1%	70.9%			
		Lose	Frequency	11	41			
			Percentage	21.2%	78.8%			
High	3P	Win	Frequency	23	45	3.902*	0.048	0.159
			Percentage	33.8%	66.2%			
		Lose	Frequency	17	69			
			Percentage	19.8%	80.2%			

Shooter	Screen location			Success	Fail	χ^2^ (df = 1)	*p*	*φ*
Low	PL	Win	Frequency	0	1		1.000 (**)	−0.447
			Percentage	0.0%	100.0%			
		Lose	Frequency	3	2			
			Percentage	60.0%	40.0%			
Middle	PL	Win	Frequency	15	9	0.512	0.474	0.104
			Percentage	62.5%	37.5%			
		Lose	Frequency	12	11			
			Percentage	52.2%	47.8%			
High	PL	Win	Frequency	20	8	2.232	0.135	0.201
			Percentage	71.4%	28.6%			
		Lose	Frequency	14	13			
			Percentage	51.9%	48.1%			
Low	PH	Win	Frequency	1	1		1.000 (**)	0.250
			Percentage	50.0%	50.0%			
		Lose	Frequency	3	1			
			Percentage	75.0%	25.0%			
Middle	PH	Win	Frequency	18	20	0.110	0.740	0.040
			Percentage	47.4%	52.6%			
		Lose	Frequency	13	17			
			Percentage	43.3%	56.7%			
High	PH	Win	Frequency	17	30	0.038	0.846	−0.023
			Percentage	36.2%	63.8%			
		Lose	Frequency	10	16			
			Percentage	38.5%	61.5%			
Low	Top	Win	Frequency	0	2			
			Percentage	0.0%	100.0%			
		Lose	Frequency	0	6			
			Percentage	0.0%	100.0%			
Middle	Top	Win	Frequency	14	20	0.513	0.474	0.081
			Percentage	41.2%	58.8%			
		Lose	Frequency	15	30			
			Percentage	33.3%	66.7%			
High	Top	Win	Frequency	14	20	0.304	0.582	0.056
			Percentage	41.2%	58.8%			
		Lose	Frequency	22	40			
			Percentage	35.5%	64.5%			
Low	Corner	Win	Frequency	15	15	0.025	0.875	0.022
			Percentage	50.0%	50.0%			
		Lose	Frequency	11	12			
			Percentage	47.8%	52.2%			
Middle	Corner	Win	Frequency	49	66	0.158	0.691	0.026
			Percentage	42.6%	57.4%			
		Lose	Frequency	44	66			
			Percentage	40.0%	60.0%			
High	Corner	Win	Frequency	61	54	1.624	0.203	0.088
			Percentage	53.0%	47.0%			
		Lose	Frequency	42	53			
			Percentage	44.2%	55.8%			
Low	Wing	Win	Frequency	4	15	5.602*	0.018	−0.311
			Percentage	21.1%	78.9%			
		Lose	Frequency	21	18			
			Percentage	53.8%	46.2%			
Middle	Wing	Win	Frequency	76	122	0.151	0.698	0.021
			Percentage	38.4%	61.6%			
		Lose	Frequency	56	98			
			Percentage	36.4%	63.6%			
High	Wing	Win	Frequency	75	103	5.169*	0.023	0.124
			Percentage	42.1%	57.9%			
		Lose	Frequency	47	109			
			Percentage	30.1%	69.9%			
Low	3P	Win	Frequency	1	1		1.000	0.000
			Percentage	50.0%	50.0%			
		Lose	Frequency	2	2			
			Percentage	50.0%	50.0%			
Middle	3P	Win	Frequency	11	15	0.224	0.636	−0.062
			Percentage	42.3%	57.7%			
		Lose	Frequency	16	17			
			Percentage	48.5%	51.5%			
High	3P	Win	Frequency	15	9	2.289	0.130	0.204
			Percentage	62.5%	37.5%			
		Lose	Frequency	13	18			
			Percentage	41.9%	58.1%			

Shooter	Pass location			Success	Fail	χ^2^ (df = 1)	*p*	*φ*
Low	PL	Win	Frequency	0	1			
			Percentage	0.0%	100.0%			
		Lose	Frequency	0	1			
			Percentage	0.0%	100.0%			
Middle	PL	Win	Frequency	6	8		0.628 (**)	−0.218
			Percentage	42.9%	57.1%			
		Lose	Frequency	4	2			
			Percentage	66.7%	33.3%			
High	PL	Win	Frequency	5	3		0.592 (**)	0.220
			Percentage	62.5%	37.5%			
		Lose	Frequency	2	3			
			Percentage	40.0%	60.0%			
Low	PH	Win	Frequency	4	6		0.335 (**)	−0.310
			Percentage	40.0%	60.0%			
		Lose	Frequency	5	2			
			Percentage	71.4%	28.6%			
Middle	PH	Win	Frequency	18	24	0.449	0.503	0.073
			Percentage	42.9%	57.1%			
		Lose	Frequency	15	27			
			Percentage	35.7%	64.3%			
High	PH	Win	Frequency	19	14	3.264	0.071	0.251
			Percentage	57.6%	42.4%			
		Lose	Frequency	6	13			
			Percentage	31.6%	68.4%			
Low	Top	Win	Frequency	6	6	0.540	0.462	−0.144
			Percentage	50.0%	50.0%			
		Lose	Frequency	9	5			
			Percentage	64.3%	35.7%			
Middle	Top	Win	Frequency	16	29	5.129*	0.024	−0.229
			Percentage	35.6%	64.4%			
		Lose	Frequency	31	22			
			Percentage	58.5%	41.5%			
High	Top	Win	Frequency	17	21	0.190	0.663	0.048
			Percentage	44.7%	55.3%			
		Lose	Frequency	18	27			
			Percentage	40.0%	60.0%			
Low	Corner	Win	Frequency	9	6	0.157	0.692	−0.069
			Percentage	60.0%	40.0%			
		Lose	Frequency	12	6			
			Percentage	66.7%	33.3%			
Middle	Corner	Win	Frequency	23	43	0.187	0.665	−0.040
			Percentage	34.8%	65.2%			
		Lose	Frequency	19	30			
			Percentage	38.8%	61.2%			
High	Corner	Win	Frequency	23	26	0.522	0.470	0.081
			Percentage	46.9%	53.1%			
		Lose	Frequency	12	19			
			Percentage	38.7%	61.3%			
Low	Wing	Win	Frequency	4	13	1.710	0.191	−0.207
			Percentage	23.5%	76.5%			
		Lose	Frequency	10	13			
			Percentage	43.5%	56.5%			
Middle	Wing	Win	Frequency	39	34	1.431	0.232	0.097
			Percentage	53.4%	46.6%			
		Lose	Frequency	35	45			
			Percentage	43.8%	56.3%			
High	Wing	Win	Frequency	26	43	1.193	0.275	−0.094
			Percentage	37.7%	62.3%			
		Lose	Frequency	31	35			
			Percentage	47.0%	53.0%			
Low	3P	Win	Frequency	1	1		0.295 (**)	0.409
			Percentage	50.0%	50.0%			
		Lose	Frequency	1	10			
			Percentage	9.1%	90.9%			
Middle	3P	Win	Frequency	20	20	1.810	0.179	0.146
			Percentage	50.0%	50.0%			
		Lose	Frequency	16	29			
			Percentage	35.6%	64.4%			
High	3P	Win	Frequency	26	23	3.191	0.074	0.170
			Percentage	53.1%	46.9%			
		Lose	Frequency	22	39			
			Percentage	36.1%	63.9%			

Shooter	Type of screen			Success	Fail	χ^2^ (df = 1)	*p*	*φ*
Low	On-the-ball screen	Win	Frequency	19	29	0.736	0.391	−0.081
			Percentage	39.6%	60.4%			
		Lose	Frequency	31	34			
			Percentage	47.7%	52.3%			
Middle	On-the-ball screen	Win	Frequency	133	196	0.080	0.777	0.011
			Percentage	40.4%	59.6%			
		Lose	Frequency	114	176			
			Percentage	39.3%	60.7%			
High	On-the-ball screen	Win	Frequency	140	175	4.621*	0.032	0.087
			Percentage	44.4%	55.6%			
		Lose	Frequency	104	186			
			Percentage	35.9%	64.1%			
Low	Off-the-ball screen	Win	Frequency	5	7	0.181	0.671	−0.083
			Percentage	41.7%	58.3%			
		Lose	Frequency	7	7			
			Percentage	50.0%	50.0%			
Middle	Off-the-ball screen	Win	Frequency	51	60	0.737	0.391	0.058
			Percentage	45.9%	54.1%			
		Lose	Frequency	43	64			
			Percentage	40.2%	59.8%			
High	Off-the-ball screen	Win	Frequency	65	56	4.032*	0.045	0.132
			Percentage	53.7%	46.3%			
		Lose	Frequency	45	66			
			Percentage	40.5%	59.5%			

Shooter	Type of screen-play			Success	Fail	χ^2^ (df = 1)	*p*	*φ*
Low	ON-U	Win	Frequency	0	4		1.000 (**)	−0.272
			Percentage	0.0%	100.0%			
		Lose	Frequency	1	5			
			Percentage	16.7%	83.3%			
Middle	ON-U	Win	Frequency	63	99	1.468	0.226	0.071
			Percentage	38.9%	61.1%			
		Lose	Frequency	42	89			
			Percentage	32.1%	67.9%			
High	ON-U	Win	Frequency	91	103	6.638*	0.010	0.134
			Percentage	46.9%	53.1%			
		Lose	Frequency	59	116			
			Percentage	33.7%	66.3%			
Low	ON-S	Win	Frequency	7	7	1.146	0.284	−0.186
			Percentage	50.0%	50.0%			
		Lose	Frequency	13	6			
			Percentage	68.4%	31.6%			
Middle	ON-S	Win	Frequency	12	18	2.200	0.138	−0.183
			Percentage	40.0%	60.0%			
		Lose	Frequency	21	15			
			Percentage	58.3%	41.7%			
High	ON-S	Win	Frequency	12	13	0.242	0.622	0.078
			Percentage	48.0%	52.0%			
		Lose	Frequency	6	9			
			Percentage	40.0%	60.0%			
Low	ON-A	Win	Frequency	10	12	0.023	0.879	0.021
			Percentage	45.5%	54.5%			
		Lose	Frequency	13	17			
			Percentage	43.3%	56.7%			
Middle	ON-A	Win	Frequency	44	60	1.108	0.292	0.078
			Percentage	42.3%	57.7%			
		Lose	Frequency	27	51			
			Percentage	34.6%	65.4%			
High	ON-A	Win	Frequency	20	32	0.016	0.898	0.012
			Percentage	38.5%	61.5%			
		Lose	Frequency	25	42			
			Percentage	37.3%	62.7%			
Low	ON-E	Win	Frequency	2	6		0.638 (**)	−0.158
			Percentage	25.0%	75.0%			
		Lose	Frequency	4	6			
			Percentage	40.0%	60.0%			
Middle	ON-E	Win	Frequency	14	19	0.907	0.341	−0.108
			Percentage	42.4%	57.6%			
		Lose	Frequency	24	21			
			Percentage	53.3%	46.7%			
High	ON-E	Win	Frequency	17	27	0.112	0.737	−0.038
			Percentage	38.6%	61.4%			
		Lose	Frequency	14	19			
			Percentage	42.4%	57.6%			
Low	OF-U	Win	Frequency	5	6		0.670 (**)	−0.145
			Percentage	45.5%	54.5%			
		Lose	Frequency	6	4			
			Percentage	60.0%	40.0%			
Middle	OF-U	Win	Frequency	42	56	0.273	0.601	0.037
			Percentage	42.9%	57.1%			
		Lose	Frequency	38	59			
			Percentage	39.2%	60.8%			
High	OF-U	Win	Frequency	61	56	3.278	0.070	0.122
			Percentage	52.1%	47.9%			
		Lose	Frequency	42	63			
			Percentage	40.0%	60.0%			
Low	OF-S	Win	Frequency	0	1		0.295 (**)	−0.250
			Percentage	0.0%	100.0%			
		Lose	Frequency	1	3			
			Percentage	25.0%	75.0%			
Middle	OF-S	Win	Frequency	9	4		0.417 (**)	0.195
			Percentage	69.2%	30.8%			
		Lose	Frequency	5	5			
			Percentage	50.0%	50.0%			
High	OF-S	Win	Frequency	4	0		0.091 (**)	0.535
			Percentage	100.0%	0.0%			
		Lose	Frequency	3	3			
			Percentage	50.0%	50.0%			

Shooter	Movement of on- the-ball screen plays			Success	Fail	χ^2^ (df = 1)	*p*	*φ*
Low	Around	Win	Frequency	1	2		1.000 (**)	−0.333
			Percentage	33.3%	66.7%			
		Lose	Frequency	2	1			
			Percentage	66.7%	33.3%			
Middle	Around	Win	Frequency	3	1		0.569 (**)	0.262
			Percentage	75.0%	25.0%			
		Lose	Frequency	5	6			
			Percentage	45.5%	54.5%			
High	Around	Win	Frequency	4	8		1.000 (**)	0.000
			Percentage	33.3%	66.7%			
		Lose	Frequency	2	4			
			Percentage	33.3%	66.7%			
Low	Center-line	Win	Frequency	10	15	0.394	0.530	−0.084
			Percentage	40.0%	60.0%			
		Lose	Frequency	15	16			
			Percentage	48.4%	51.6%			
Middle	Center-line	Win	Frequency	58	91	0.484	0.486	0.042
			Percentage	38.9%	61.1%			
		Lose	Frequency	45	84			
			Percentage	34.9%	65.1%			
High	Center-line	Win	Frequency	62	63	4.722*	0.030	0.137
			Percentage	49.6%	50.4%			
		Lose	Frequency	45	80			
			Percentage	36.0%	64.0%			
Low	End-line	Win	Frequency	8	10	0.022	0.881	−0.022
			Percentage	44.4%	55.6%			
		Lose	Frequency	14	16			
			Percentage	46.7%	53.3%			
Middle	End-line	Win	Frequency	70	98	0.003	0.959	−0.003
			Percentage	41.7%	58.3%			
		Lose	Frequency	60	83			
			Percentage	42.0%	58.0%			
High	End-line	Win	Frequency	70	91	1.892	0.169	0.078
			Percentage	43.5%	56.5%			
		Lose	Frequency	53	95			
			Percentage	35.8%	64.2%			
Low	ON-Down	Win	Frequency	0	2			
			Percentage	0.0%	100.0%			
		Lose	Frequency	0	1			
			Percentage	0.0%	100.0%			
Middle	ON-Down	Win	Frequency	2	6		0.315 (**)	−0.327
			Percentage	25.0%	75.0%			
		Lose	Frequency	4	3			
			Percentage	57.1%	42.9%			
High	ON-Down	Win	Frequency	3	13		0.391 (**)	−0.197
			Percentage	18.8%	81.3%			
		Lose	Frequency	4	7			
			Percentage	36.4%	63.6%			

Shooter	User			Success	Fail	χ^2^ (df = 1)	*p*	*φ*
Low	Low	Win	Frequency	5	10	0.354	0.552	−0.107
			Percentage	33.3%	66.7%			
		Lose	Frequency	7	9			
			Percentage	43.8%	56.3%			
Low	Middle	Win	Frequency	13	12	0.012	0.914	0.018
			Percentage	52.0%	48.0%			
		Lose	Frequency	7	6			
			Percentage	53.8%	46.2%			
Low	High	Win	Frequency	12	20	0.691	0.406	−0.099
			Percentage	37.5%	62.5%			
		Lose	Frequency	18	20			
			Percentage	47.4%	52.6%			
Middle	Low	Win	Frequency	1	1		1.000 (**)	0.250
			Percentage	50.0%	50.0%			
		Lose	Frequency	1	3			
			Percentage	25.0%	75.0%			
Middle	Middle	Win	Frequency	136	191	1.022	0.312	0.040
			Percentage	41.6%	58.4%			
		Lose	Frequency	116	192			
			Percentage	37.7%	62.3%			
Middle	High	Win	Frequency	47	64	0.434	0.510	−0.047
			Percentage	42.3%	57.7%			
		Lose	Frequency	40	45			
			Percentage	47.1%	52.9%			
High	Low	Win	Frequency	1	1		1.000 (**)	−0.091
			Percentage	50.0%	50.0%			
		Lose	Frequency	3	2			
			Percentage	60.0%	40.0%			
High	Middle	Win	Frequency	30	39	0.598	0.439	0.067
			Percentage	43.5%	56.5%			
		Lose	Frequency	24	41			
			Percentage	36.9%	63.1%			
High	High	Win	Frequency	174	191	8.304*	0.004	0.109
			Percentage	47.7%	52.3%			
		Lose	Frequency	122	209			
			Percentage	36.9%	63.1%			
Shooter	Screener			Success	Fail	χ^2^ (df = 1)	*p*	*φ*
Low	Low	Win	Frequency	7	11	0.450	0.502	−0.092
			Percentage	38.9%	61.1%			
		Lose	Frequency	17	18			
			Percentage	48.6%	51.4%			
Low	Middle	Win	Frequency	9	15	2.407	0.121	−0.217
			Percentage	37.5%	62.5%			
		Lose	Frequency	16	11			
			Percentage	59.3%	40.7%			
Low	High	Win	Frequency	7	9	0.732	0.392	0.149
			Percentage	43.8%	56.3%			
		Lose	Frequency	5	12			
			Percentage	29.4%	70.6%			
Middle	Low	Win	Frequency	46	51	0.718	0.397	0.059
			Percentage	47.4%	52.6%			
		Lose	Frequency	44	62			
			Percentage	41.5%	58.5%			
Middle	Middle	Win	Frequency	89	140	0.014	0.905	−0.006
			Percentage	38.9%	61.1%			
		Lose	Frequency	82	126			
			Percentage	39.4%	60.6%			
Middle	High	Win	Frequency	48	61	0.941	0.332	0.070
			Percentage	44.0%	56.0%			
		Lose	Frequency	30	51			
			Percentage	37.0%	63.0%			
High	Low	Win	Frequency	60	62	1.981	0.159	0.086
			Percentage	49.2%	50.8%			
		Lose	Frequency	58	85			
			Percentage	40.6%	59.4%			
High	Middle	Win	Frequency	97	107	5.503*	0.019	0.121
			Percentage	47.5%	52.5%			
		Lose	Frequency	60	109			
			Percentage	35.5%	64.5%			
High	High	Win	Frequency	45	55	1.796	0.180	0.099
			Percentage	45.0%	55.0%			
		Lose	Frequency	30	55			
			Percentage	35.3%	64.7%			

PC, player classification; Low, low-point classification; Middle, middle-point classification; High, high-point classification; PL, paint-low; PH, paint-high; 3P, 3-point field goal area; ON-U, the plays where the user shot using the on-the-ball screen; ON-S, plays where the screener of the on-the-ball screen shot after receiving a pass from the user; ON-A, plays where another player shot after receiving a pass from the user of the on-the-ball screen; ON-E, plays that led to a shot through two or more extra passes after the user used the on-the-ball screen; OF-U, plays where the user shot using the off-the-ball screen; OF-S, plays where the screener of the off-the-ball screen shot; Around, plays where the screener held a ball; Center-line, plays where the user moved toward the center-line side against the screener; End-line, plays where the user moved toward the end-line side against the screener; ON-Down, plays where the screener was on the center-line side of the defense who protected the user holding a ball in a Top or 3P on Top extension.

* *p* < 0.05.

** We adopted *p*-value by Fisher's method.

**Table 5:** Differences in shot-success rate for each categorical variable depending on the PC of the user.

User	Shot location			Success	Fail	χ^2^ (df = 1)	*p*	*φ*
Low	PL	Win	Frequency	0	1		1.000 (**)	−0.447
			Percentage	0.0%	100.0%			
		Lose	Frequency	3	2			
			Percentage	60.0%	40.0%			
Middle	PL	Win	Frequency	55	43	0.024	0.876	0.011
			Percentage	56.1%	43.9%			
		Lose	Frequency	60	49			
			Percentage	55.0%	45.0%			
High	PL	Win	Frequency	87	63	0.040	0.841	0.012
			Percentage	58.0%	42.0%			
		Lose	Frequency	71	54			
			Percentage	56.8%	43.2%			
Low	PH	Win	Frequency	0	0			
			Percentage	0.0%	0.0%			
		Lose	Frequency	0	2			
			Percentage	0.0%	100.0%			
Middle	PH	Win	Frequency	13	14	2.270	0.132	0.184
			Percentage	48.1%	51.9%			
		Lose	Frequency	12	28			
			Percentage	30.0%	70.0%			
High	PH	Win	Frequency	24	23	6.206*	0.013	0.272
			Percentage	51.1%	48.9%			
		Lose	Frequency	9	28			
			Percentage	24.3%	75.7%			
Low	Top	Win	Frequency	0	1		1.000 (**)	−0.408
			Percentage	0.0%	100.0%			
		Lose	Frequency	2	2			
			Percentage	50.0%	50.0%			
Middle	Top	Win	Frequency	14	23	0.003	0.955	−0.006
			Percentage	37.8%	62.2%			
		Lose	Frequency	15	24			
			Percentage	38.5%	61.5%			
High	Top	Win	Frequency	21	32	0.146	0.703	−0.040
			Percentage	39.6%	60.4%			
		Lose	Frequency	17	22			
			Percentage	43.6%	56.4%			
Low	Corner	Win	Frequency	5	5		1.000 (**)	0.101
			Percentage	50.0%	50.0%			
		Lose	Frequency	4	6			
			Percentage	40.0%	60.0%			
Middle	Corner	Win	Frequency	40	53	0.012	0.912	−0.008
			Percentage	43.0%	57.0%			
		Lose	Frequency	39	50			
			Percentage	43.8%	56.2%			
High	Corner	Win	Frequency	45	54	0.036	0.849	0.014
			Percentage	45.5%	54.5%			
		Lose	Frequency	41	52			
			Percentage	44.1%	55.9%			
Low	Wing	Win	Frequency	2	4		0.524 (**)	−0.316
			Percentage	33.3%	66.7%			
		Lose	Frequency	2	1			
			Percentage	66.7%	33.3%			
Middle	Wing	Win	Frequency	37	63	3.838	0.050	0.150
			Percentage	37.0%	63.0%			
		Lose	Frequency	16	54			
			Percentage	22.9%	77.1%			
High	Wing	Win	Frequency	31	59	0.008	0.929	0.007
			Percentage	34.4%	65.6%			
		Lose	Frequency	25	49			
			Percentage	33.8%	66.2%			
Low	3P	Win	Frequency	0	1			
			Percentage	0.0%	100.0%			
		Lose	Frequency	0	1			
			Percentage	0.0%	100.0%			
Middle	3P	Win	Frequency	14	40	0.275	0.600	0.051
			Percentage	25.9%	74.1%			
		Lose	Frequency	11	40			
			Percentage	21.6%	78.4%			
High	3P	Win	Frequency	25	44	5.253*	0.022	0.184
			Percentage	36.2%	63.8%			
		Lose	Frequency	17	69			
			Percentage	19.8%	80.2%			

User	Pass location			Success	Fail	χ^2^ (df = 1)	*p*	*φ*
Low	PL	Win	Frequency	0	0			
			Percentage	0.0%	0.0%			
		Lose	Frequency	0	1			
			Percentage	0.0%	100.0%			
Middle	PL	Win	Frequency	6	4		1.000 (**)	0.045
			Percentage	60.0%	40.0%			
		Lose	Frequency	5	4			
			Percentage	55.6%	44.4%			
High	PL	Win	Frequency	5	7		1.000 (**)	−0.059
			Percentage	41.7%	58.3%			
		Lose	Frequency	1	1			
			Percentage	50.0%	50.0%			
Low	PH	Win	Frequency	0	2		1.000 (**)	−0.577
			Percentage	0.0%	100.0%			
		Lose	Frequency	1	1			
			Percentage	50.0%	50.0%			
Middle	PH	Win	Frequency	12	18	1.200	0.273	0.141
			Percentage	40.0%	60.0%			
		Lose	Frequency	8	22			
			Percentage	26.7%	73.3%			
High	PH	Win	Frequency	29	24	0.482	0.487	0.074
			Percentage	54.7%	45.3%			
		Lose	Frequency	17	19			
			Percentage	47.2%	52.8%			
Low	Top	Win	Frequency	5	4		1.000 (**)	−0.016
			Percentage	55.6%	44.4%			
		Lose	Frequency	4	3			
			Percentage	57.1%	42.9%			
Middle	Top	Win	Frequency	20	27	2.263	0.132	−0.151
			Percentage	42.6%	57.4%			
		Lose	Frequency	30	22			
			Percentage	57.7%	42.3%			
High	Top	Win	Frequency	14	25	0.816	0.366	−0.094
			Percentage	35.9%	64.1%			
		Lose	Frequency	24	29			
			Percentage	45.3%	54.7%			
Low	Corner	Win	Frequency	0	1	2.000	0.157	−1.000
			Percentage	0.0%	100.0%			
		Lose	Frequency	1	0			
			Percentage	100.0%	0.0%			
Middle	Corner	Win	Frequency	24	31	0.044	0.833	−0.020
			Percentage	43.6%	56.4%			
		Lose	Frequency	26	31			
			Percentage	45.6%	54.4%			
High	Corner	Win	Frequency	31	43	0.038	0.845	0.018
			Percentage	41.9%	58.1%			
		Lose	Frequency	16	24			
			Percentage	40.0%	60.0%			
Low	Wing	Win	Frequency	0	0			
			Percentage	0.0%	0.0%			
		Lose	Frequency	3	1			
			Percentage	75.0%	25.0%			
Middle	Wing	Win	Frequency	32	39	0.522	0.470	0.059
			Percentage	45.1%	54.9%			
		Lose	Frequency	31	48			
			Percentage	39.2%	60.8%			
High	Wing	Win	Frequency	37	51	0.809	0.368	−0.068
			Percentage	42.0%	58.0%			
		Lose	Frequency	42	44			
			Percentage	48.8%	51.2%			
Low	3P	Win	Frequency	1	0		0.333 (**)	0.632
			Percentage	100.0%	0.0%			
		Lose	Frequency	1	4			
			Percentage	20.0%	80.0%			
Middle	3P	Win	Frequency	17	18	1.962	0.161	0.154
			Percentage	48.6%	51.4%			
		Lose	Frequency	16	32			
			Percentage	33.3%	66.7%			
High	3P	Win	Frequency	29	26	4.068*	0.044	0.185
			Percentage	52.7%	47.3%			
		Lose	Frequency	22	42			
			Percentage	34.4%	65.6%			
User	Type of screen			Success	Fail	χ^2^ (df = 1)	*p*	*φ*
Low	On-the-ball screen	Win	Frequency	1	6		1.000 (**)	−0.127
			Percentage	14.3%	85.7%			
		Lose	Frequency	3	9			
			Percentage	25.0%	75.0%			
Middle	On-the-ball screen	Win	Frequency	122	178	0.577	0.448	0.031
			Percentage	40.7%	59.3%			
		Lose	Frequency	111	184			
			Percentage	37.6%	62.4%			
High	On-the-ball screen	Win	Frequency	169	216	1.156	0.282	0.040
			Percentage	43.9%	56.1%			
		Lose	Frequency	135	203			
			Percentage	39.9%	60.1%			
Low	Off-the-ball screen	Win	Frequency	6	6	0.337	0.561	−0.116
			Percentage	50.0%	50.0%			
		Lose	Frequency	8	5			
			Percentage	61.5%	38.5%			
Middle	Off-the-ball screen	Win	Frequency	51	58	0.777	0.378	0.061
			Percentage	46.8%	53.2%			
		Lose	Frequency	42	61			
			Percentage	40.8%	59.2%			
High	Off-the-ball screen	Win	Frequency	64	59	4.218*	0.040	0.133
			Percentage	52.0%	48.0%			
		Lose	Frequency	45	71			
			Percentage	38.8%	61.2%			

User	Type of screen-play			Success	Fail	χ^2^ (df = 1)	*p*	*φ*
Low	ON-U	Win	Frequency	0	4		1.000 (**)	−0.272
			Percentage	0.0%	100.0%			
		Lose	Frequency	1	5			
			Percentage	16.7%	83.3%			
Middle	ON-U	Win	Frequency	63	99	1.468	0.226	0.071
			Percentage	38.9%	61.1%			
		Lose	Frequency	42	89			
			Percentage	32.1%	67.9%			
High	ON-U	Win	Frequency	91	103	6.638*	0.010	0.134
			Percentage	46.9%	53.1%			
		Lose	Frequency	59	116			
			Percentage	33.7%	66.3%			
Low	ON-S	Win	Frequency	1	1		1.000 (**)	0.577
			Percentage	50.0%	50.0%			
		Lose	Frequency	0	2			
			Percentage	0.0%	100.0%			
Middle	ON-S	Win	Frequency	12	20	3.065	0.080	−0.219
			Percentage	37.5%	62.5%			
		Lose	Frequency	19	13			
			Percentage	59.4%	40.6%			
High	ON-S	Win	Frequency	18	17	0.342	0.559	−0.069
			Percentage	51.4%	48.6%			
		Lose	Frequency	21	15			
			Percentage	58.3%	41.7%			
Low	ON-A	Win	Frequency	0	1		1.000 (**)	−0.333
			Percentage	0.0%	100.0%			
		Lose	Frequency	1	2			
			Percentage	33.3%	66.7%			
Middle	ON-A	Win	Frequency	29	32	2.802	0.094	0.140
			Percentage	47.5%	52.5%			
		Lose	Frequency	28	55			
			Percentage	33.7%	66.3%			
High	ON-A	Win	Frequency	45	71	0.058	0.810	−0.017
			Percentage	38.8%	61.2%			
		Lose	Frequency	36	53			
			Percentage	40.4%	59.6%			
Low	ON-E	Win	Frequency	0	0			
			Percentage	0.0%	0.0%			
		Lose	Frequency	1	0			
			Percentage	100.0%	0.0%			
Middle	ON-E	Win	Frequency	18	27	0.230	0.631	−0.049
			Percentage	40.0%	60.0%			
		Lose	Frequency	22	27			
			Percentage	44.9%	55.1%			
High	ON-E	Win	Frequency	15	25	1.238	0.266	−0.126
			Percentage	37.5%	62.5%			
		Lose	Frequency	19	19			
			Percentage	50.0%	50.0%			
Low	OF-U	Win	Frequency	5	6		0.670 (**)	−0.145
			Percentage	45.5%	54.5%			
		Lose	Frequency	6	4			
			Percentage	60.0%	40.0%			
Middle	OF-U	Win	Frequency	42	56	0.273	0.601	0.037
			Percentage	42.9%	57.1%			
		Lose	Frequency	38	59			
			Percentage	39.2%	60.8%			
High	OF-U	Win	Frequency	61	56	3.278	0.070	0.122
			Percentage	52.1%	47.9%			
		Lose	Frequency	42	63			
			Percentage	40.0%	60.0%			
Low	OF-S	Win	Frequency	1	0		1.000 (**)	0.333
			Percentage	100.0%	0.0%			
		Lose	Frequency	2	1			
			Percentage	66.7%	33.3%			
Middle	OF-S	Win	Frequency	9	2		0.584 (**)	0.171
			Percentage	81.8%	18.2%			
		Lose	Frequency	4	2			
			Percentage	66.7%	33.3%			
High	OF-S	Win	Frequency	3	3		0.600 (**)	0.227
			Percentage	50.0%	50.0%			
		Lose	Frequency	3	8			
			Percentage	27.3%	72.7%			

User	Movement of off-the- ball screen plays			Success	Fail	χ^2^ (df = 1)	*p*	*φ*
Low	Back	Win	Frequency	1	0			
			Percentage	100.0%	0.0%			
		Lose	Frequency	2	0			
			Percentage	100.0%	0.0%			
Middle	Back	Win	Frequency	14	11	1.481	0.224	0.167
			Percentage	56.0%	44.0%			
		Lose	Frequency	11	17			
			Percentage	39.3%	60.7%			
High	Back	Win	Frequency	14	12	2.932	0.087	0.231
			Percentage	53.8%	46.2%			
		Lose	Frequency	9	20			
			Percentage	31.0%	69.0%			
Low	Cross	Win	Frequency	1	0			
			Percentage	100.0%	0.0%			
		Lose	Frequency	1	0			
			Percentage	100.0%	0.0%			
Middle	Cross	Win	Frequency	5	3		1.000 (**)	−0.135
			Percentage	62.5%	37.5%			
		Lose	Frequency	6	2			
			Percentage	75.0%	25.0%			
High	Cross	Win	Frequency	11	5	1.094	0.296	0.191
			Percentage	68.8%	31.3%			
		Lose	Frequency	7	7			
			Percentage	50.0%	50.0%			
Low	Down	Win	Frequency	0	1			
			Percentage	0.0%	100.0%			
		Lose	Frequency	0	0			
			Percentage	0.0%	0.0%			
Middle	Down	Win	Frequency	12	11	4.057*	0.044	0.291
			Percentage	52.2%	47.8%			
		Lose	Frequency	6	19			
			Percentage	24.0%	76.0%			
High	Down	Win	Frequency	15	15	1.984	0.159	0.180
			Percentage	50.0%	50.0%			
		Lose	Frequency	10	21			
			Percentage	32.3%	67.7%			
Low	Flare	Win	Frequency	4	5		1.000 (**)	−0.056
			Percentage	44.4%	55.6%			
		Lose	Frequency	5	5			
			Percentage	50.0%	50.0%			
Middle	Flare	Win	Frequency	19	33	0.730	0.393	−0.088
			Percentage	36.5%	63.5%			
		Lose	Frequency	19	23			
			Percentage	45.2%	54.8%			
High	Flare	Win	Frequency	24	27	0.031	0.861	0.018
			Percentage	47.1%	52.9%			
		Lose	Frequency	19	23			
			Percentage	45.2%	54.8%			

PC, player classification; Low, low-point classification; Middle, middle-point classification; High, high-point classification; PL, paint-low; PH, paint-high; 3P, 3-point field goal area; ON-U, the plays where the user shot using the on-the-ball screen; ON-S, plays where the screener of the on-the-ball screen shot after receiving a pass from the user; ON-A, plays where another player shot after receiving a pass from the user of the on-the-ball screen; ON-E, plays that led to a shot through two or more extra passes after the user used the on-the-ball screen; OF-U, plays where the user shot using the off-the-ball screen; OF-S, plays where the screener of the off-the-ball screen shot; Back, plays where the screener was on the end-line side of the defense who protected the user; Cross, plays where the screener was on the middle-line (the imaginary line connecting baskets running through the center of the court) side of the defense who protected the user; Down, plays where the screener on the center-line side of the defense who protected the user; Flare, plays where the screener on the side-line side of the defense who protected the user.

* *p* < 0.05.

** We adopted *p*-value by Fisher's method.

**Table 6:** Differences in shot-success rate for each categorical variable depending on the PC of the screener.

Screener	Shot location			Success	Fail	χ^2^ (df = 1)	*p*	*φ*
Low	PL	Win	Frequency	45	37	0.427	0.514	−0.050
			Percentage	54.9%	45.1%			
		Lose	Frequency	55	37			
			Percentage	59.8%	40.2%			
Middle	PL	Win	Frequency	67	48	0.282	0.595	0.036
			Percentage	58.3%	41.7%			
		Lose	Frequency	58	48			
			Percentage	54.7%	45.3%			
High	PL	Win	Frequency	30	22	0.388	0.533	0.065
			Percentage	57.7%	42.3%			
		Lose	Frequency	21	20			
			Percentage	51.2%	48.8%			
Low	PH	Win	Frequency	11	9	9.586*	0.002	0.462
			Percentage	55.0%	45.0%			
		Lose	Frequency	3	22			
			Percentage	12.0%	88.0%			
Middle	PH	Win	Frequency	14	24	0.019	0.891	0.016
			Percentage	36.8%	63.2%			
		Lose	Frequency	12	22			
			Percentage	35.3%	64.7%			
High	PH	Win	Frequency	12	4	7.200*	0.007	0.447
			Percentage	75.0%	25.0%			
		Lose	Frequency	6	14			
			Percentage	30.0%	70.0%			
Low	Top	Win	Frequency	4	10	1.106	0.293	−0.189
			Percentage	28.6%	71.4%			
		Lose	Frequency	8	9			
			Percentage	47.1%	52.9%			
Middle	Top	Win	Frequency	22	30	0.096	0.757	−0.032
			Percentage	42.3%	57.7%			
		Lose	Frequency	20	24			
			Percentage	45.5%	54.5%			
High	Top	Win	Frequency	9	16	0.287	0.592	0.079
			Percentage	36.0%	64.0%			
		Lose	Frequency	6	15			
			Percentage	28.6%	71.4%			
Low	Corner	Win	Frequency	29	32	0.012	0.911	0.010
			Percentage	47.5%	52.5%			
		Lose	Frequency	34	39			
			Percentage	46.6%	53.4%			
Middle	Corner	Win	Frequency	31	56	2.206	0.137	−0.115
			Percentage	35.6%	64.4%			
		Lose	Frequency	38	43			
			Percentage	46.9%	53.1%			
High	Corner	Win	Frequency	28	22	4.322*	0.038	0.224
			Percentage	56.0%	44.0%			
		Lose	Frequency	12	24			
			Percentage	33.3%	66.7%			
Low	Wing	Win	Frequency	19	24	1.792	0.181	0.144
			Percentage	44.2%	55.8%			
		Lose	Frequency	13	30			
			Percentage	30.2%	69.8%			
Middle	Wing	Win	Frequency	35	63	1.518	0.218	0.095
			Percentage	35.7%	64.3%			
		Lose	Frequency	19	52			
			Percentage	26.8%	73.2%			
High	Wing	Win	Frequency	15	39	0.415	0.520	−0.069
			Percentage	27.8%	72.2%			
		Lose	Frequency	11	21			
			Percentage	34.4%	65.6%			
Low	3P	Win	Frequency	5	12		0.472 (**)	0.135
			Percentage	29.4%	70.6%			
		Lose	Frequency	6	28			
			Percentage	17.6%	82.4%			
Middle	3P	Win	Frequency	26	41	8.686*	0.003	0.254
			Percentage	38.8%	61.2%			
		Lose	Frequency	11	57			
			Percentage	16.2%	83.8%			
High	3P	Win	Frequency	6	22	0.279	0.597	−0.068
			Percentage	21.4%	78.6%			
		Lose	Frequency	9	24			
			Percentage	27.3%	72.7%			

Screener	Pass location			Success	Fail	χ^2^ (df = 1)	*p*	*φ*
Low	PL	Win	Frequency	3	4		1.000 (**)	−0.169
			Percentage	42.9%	57.1%			
		Lose	Frequency	3	2			
			Percentage	60.0%	40.0%			
Middle	PL	Win	Frequency	6	5		1.000 (**)	−0.051
			Percentage	54.5%	45.5%			
		Lose	Frequency	3	2			
			Percentage	60.0%	40.0%			
High	PL	Win	Frequency	2	2		0.467 (**)	0.500
			Percentage	50.0%	50.0%			
		Lose	Frequency	0	2			
			Percentage	0.0%	100.0%			
Low	PH	Win	Frequency	14	13	0.017	0.897	0.018
			Percentage	51.9%	48.1%			
		Lose	Frequency	11	11			
			Percentage	50.0%	50.0%			
Middle	PH	Win	Frequency	21	21	2.957	0.086	0.199
			Percentage	50.0%	50.0%			
		Lose	Frequency	10	23			
			Percentage	30.3%	69.7%			
High	PH	Win	Frequency	6	10		1.000 (**)	−0.010
			Percentage	37.5%	62.5%			
		Lose	Frequency	5	8			
			Percentage	38.5%	61.5%			
Low	Top	Win	Frequency	14	12	0.161	0.688	0.047
			Percentage	53.8%	46.2%			
		Lose	Frequency	23	24			
			Percentage	48.9%	51.1%			
Middle	Top	Win	Frequency	13	29	5.365*	0.021	−0.238
			Percentage	31.0%	69.0%			
		Lose	Frequency	29	24			
			Percentage	54.7%	45.3%			
High	Top	Win	Frequency	12	15	0.103	0.748	−0.051
			Percentage	44.4%	55.6%			
		Lose	Frequency	6	6			
			Percentage	50.0%	50.0%			
Low	Corner	Win	Frequency	18	27	0.049	0.825	0.025
			Percentage	40.0%	60.0%			
		Lose	Frequency	12	20			
			Percentage	37.5%	62.5%			
Middle	Corner	Win	Frequency	21	31	0.415	0.520	−0.065
			Percentage	40.4%	59.6%			
		Lose	Frequency	22	25			
			Percentage	46.8%	53.2%			
High	Corner	Win	Frequency	16	17	0.006	0.938	0.011
			Percentage	48.5%	51.5%			
		Lose	Frequency	9	10			
			Percentage	47.4%	52.6%			
Low	Wing	Win	Frequency	21	26	0.141	0.707	−0.036
			Percentage	44.7%	55.3%			
		Lose	Frequency	29	31			
			Percentage	48.3%	51.7%			
Middle	Wing	Win	Frequency	24	44	1.220	0.269	−0.093
			Percentage	35.3%	64.7%			
		Lose	Frequency	32	40			
			Percentage	44.4%	55.6%			
High	Wing	Win	Frequency	22	19	1.104	0.293	0.120
			Percentage	53.7%	46.3%			
		Lose	Frequency	15	21			
			Percentage	41.7%	58.3%			
Low	3P	Win	Frequency	11	13	0.118	0.731	0.043
			Percentage	45.8%	54.2%			
		Lose	Frequency	17	24			
			Percentage	41.5%	58.5%			
Middle	3P	Win	Frequency	28	18	6.712*	0.010	0.269
			Percentage	60.9%	39.1%			
		Lose	Frequency	16	31			
			Percentage	34.0%	66.0%			
High	3P	Win	Frequency	6	12		0.493 (**)	0.141
			Percentage	33.3%	66.7%			
		Lose	Frequency	6	23			
			Percentage	20.7%	79.3%			

Screener	Type of screen- play			Success	Fail	χ^2^ (df = 1)	*p*	*φ*
Low	ON-U	Win	Frequency	32	29	6.398*	0.011	0.215
			Percentage	52.5%	47.5%			
		Lose	Frequency	24	53			
			Percentage	31.2%	68.8%			
Middle	ON-U	Win	Frequency	83	119	3.350	0.067	0.098
			Percentage	41.1%	58.9%			
		Lose	Frequency	47	102			
			Percentage	31.5%	68.5%			
High	ON-U	Win	Frequency	38	48	1.220	0.269	0.085
			Percentage	44.2%	55.8%			
		Lose	Frequency	29	52			
			Percentage	35.8%	64.2%			
Low	ON-S	Win	Frequency	7	7	1.146	0.284	−0.186
			Percentage	50.0%	50.0%			
		Lose	Frequency	13	6			
			Percentage	68.4%	31.6%			
Middle	ON-S	Win	Frequency	12	18	2.200	0.138	−0.183
			Percentage	40.0%	60.0%			
		Lose	Frequency	21	15			
			Percentage	58.3%	41.7%			
High	ON-S	Win	Frequency	12	13	0.242	0.622	0.078
			Percentage	48.0%	52.0%			
		Lose	Frequency	6	9			
			Percentage	40.0%	60.0%			
Low	ON-A	Win	Frequency	13	26	0.434	0.510	−0.068
			Percentage	33.3%	66.7%			
		Lose	Frequency	22	33			
			Percentage	40.0%	60.0%			
Middle	ON-A	Win	Frequency	44	53	1.247	0.264	0.083
			Percentage	45.4%	54.6%			
		Lose	Frequency	32	54			
			Percentage	37.2%	62.8%			
High	ON-A	Win	Frequency	17	25	0.533	0.465	0.084
			Percentage	40.5%	59.5%			
		Lose	Frequency	11	23			
			Percentage	32.4%	67.6%			
Low	ON-E	Win	Frequency	4	8		0.729 (**)	−0.106
			Percentage	33.3%	66.7%			
		Lose	Frequency	13	16			
			Percentage	44.8%	55.2%			
Middle	ON-E	Win	Frequency	18	27	2.229	0.135	−0.161
			Percentage	40.0%	60.0%			
		Lose	Frequency	23	18			
			Percentage	56.1%	43.9%			
High	ON-E	Win	Frequency	11	17	0.167	0.683	0.060
			Percentage	39.3%	60.7%			
		Lose	Frequency	6	12			
			Percentage	33.3%	66.7%			
Low	OF-U	Win	Frequency	57	53	0.710	0.400	0.058
			Percentage	51.8%	48.2%			
		Lose	Frequency	46	54			
			Percentage	46.0%	54.0%			
Middle	OF-U	Win	Frequency	29	41	0.373	0.541	0.050
			Percentage	41.4%	58.6%			
		Lose	Frequency	30	52			
			Percentage	36.6%	63.4%			
High	OF-U	Win	Frequency	18	22	0.771	0.380	0.106
			Percentage	45.0%	55.0%			
		Lose	Frequency	10	19			
			Percentage	34.5%	65.5%			
Low	OF-S	Win	Frequency	0	1		1.000 (**)	−0.250
			Percentage	0.0%	100.0%			
		Lose	Frequency	1	3			
			Percentage	25.0%	75.0%			
Middle	OF-S	Win	Frequency	9	4		0.417 (**)	0.195
			Percentage	69.2%	30.8%			
		Lose	Frequency	5	5			
			Percentage	50.0%	50.0%			
High	OF-S	Win	Frequency	4	0		0.200 (**)	0.535
			Percentage	100.0%	0.0%			
		Lose	Frequency	3	3			
			Percentage	50.0%	50.0%			

PC, player classification; Low, low-point classification; Middle, middle-point classification; High, high-point classification; PL, paint-low; PH, paint-high; 3P, 3-point field goal area; ON-U, the plays where the user shot using the on-the-ball screen; ON-S, plays where the screener of the on-the-ball screen shot after receiving a pass from the user; ON-A, plays where another player shot after receiving a pass from the user of the on-the-ball screen; ON-E, plays that led to a shot through two or more extra passes after the user used the on-the-ball screen; OF-U, plays where the user shot using the off-the-ball screen; OF-S, plays where the screener of the off-the-ball screen shot.

* *p* < 0.05.

** We adopted *p*-value by Fisher's method.

**Table 7:** Differences in shot-success rate for each categorical variable depending on the PC of the passer.

Passer	Shot location			Success	Fail	χ^2^ (df = 1)	*p*	*φ*
Low	PL	Win	Frequency	2	5		0.622 (**)	−0.214
			Percentage	28.6%	71.4%			
		Lose	Frequency	5	5			
			Percentage	50.0%	50.0%			
Middle	PL	Win	Frequency	48	34	0.463	0.496	−0.053
			Percentage	58.5%	41.5%			
		Lose	Frequency	51	29			
			Percentage	63.7%	36.3%			
High	PL	Win	Frequency	59	49	0.001	0.972	−0.002
			Percentage	54.6%	45.4%			
		Lose	Frequency	62	51			
			Percentage	54.9%	45.1%			
Low	PH	Win	Frequency	2	3			
			Percentage	40.0%	60.0%			
		Lose	Frequency	2	3			
			Percentage	40.0%	60.0%			
Middle	PH	Win	Frequency	12	10	1.773	0.183	0.188
			Percentage	54.5%	45.5%			
		Lose	Frequency	10	18			
			Percentage	35.7%	64.3%			
High	PH	Win	Frequency	12	11	3.725	0.054	0.291
			Percentage	52.2%	47.8%			
		Lose	Frequency	5	16			
			Percentage	23.8%	76.2%			
Low	Top	Win	Frequency	0	0			
			Percentage	0.0%	0.0%			
		Lose	Frequency	1	0			
			Percentage	100.0%	0.0%			
Middle	Top	Win	Frequency	10	17	0.145	0.704	−0.050
			Percentage	37.0%	63.0%			
		Lose	Frequency	13	18			
			Percentage	41.9%	58.1%			
High	Top	Win	Frequency	21	30	0.038	0.846	−0.021
			Percentage	41.2%	58.8%			
		Lose	Frequency	16	21			
			Percentage	43.2%	56.8%			
Low	Corner	Win	Frequency	5	3	0.281	0.596	0.125
			Percentage	62.5%	37.5%			
		Lose	Frequency	5	5			
			Percentage	50.0%	50.0%			
Middle	Corner	Win	Frequency	24	31	0.435	0.510	−0.064
			Percentage	43.6%	56.4%			
		Lose	Frequency	26	26			
			Percentage	50.0%	50.0%			
High	Corner	Win	Frequency	19	33	0.820	0.365	−0.083
			Percentage	36.5%	63.5%			
		Lose	Frequency	30	37			
			Percentage	44.8%	55.2%			
Low	Wing	Wi	Frequency	1	6		1.000 (**)	−0.033
			Percentage	14.3%	85.7%			
		Lose	Frequency	1	5			
			Percentage	16.7%	83.3%			
Middle	Wing	Win	Frequency	13	32	0.060	0.806	−0.027
			Percentage	28.9%	71.1%			
		Lose	Frequency	11	24			
			Percentage	31.4%	68.6%			
High	Wing	Win	Frequency	19	23	5.696*	0.017	0.277
			Percentage	45.2%	54.8%			
		Lose	Frequency	6	26			
			Percentage	18.8%	81.3%			
Low	3P	Win	Frequency	1	2			
			Percentage	33.3%	66.7%			
		Lose	Frequency	1	2			
			Percentage	33.3%	66.7%			
Middle	3P	Win	Frequency	14	19	9.074*	0.003	0.410
			Percentage	42.4%	57.6%			
		Lose	Frequency	1	20			
			Percentage	4.8%	95.2%			
High	3P	Win	Frequency	3	17		0.646 (**)	0.105
			Percentage	15.0%	85.0%			
		Lose	Frequency	2	22			
			Percentage	8.3%	91.7%			

Passer	Pass location			Success	Fail	χ^2^ (df = 1)	*p*	*φ*
Low	PL	Win	Frequency	0	2		1.000 (**)	−0.316
			Percentage	0.0%	100.0%			
		Lose	Frequency	1	3			
			Percentage	25.0%	75.0%			
Middle	PL	Win	Frequency	6	3		1.000 (**)	−0.051
			Percentage	66.7%	33.3%			
		Lose	Frequency	5	2			
			Percentage	71.4%	28.6%			
High	PL	Win	Frequency	5	6		1.000 (**)	0.255
			Percentage	45.5%	54.5%			
		Lose	Frequency	0	1			
			Percentage	0.0%	100.0%			
Low	PH	Win	Frequency	0	1		1.000 (**)	−0.408
			Percentage	0.0%	100.0%			
		Lose	Frequency	2	2			
			Percentage	50.0%	50.0%			
Middle	PH	Win	Frequency	17	19	0.040	0.842	−0.027
			Percentage	47.2%	52.8%			
		Lose	Frequency	10	10			
			Percentage	50.0%	50.0%			
High	PH	Win	Frequency	24	24	3.130	0.077	0.184
			Percentage	50.0%	50.0%			
		Lose	Frequency	14	30			
			Percentage	31.8%	68.2%			
Low	Top	Win	Frequency	5	7	0.001	0.981	−0.004
			Percentage	41.7%	58.3%			
		Lose	Frequency	8	11			
			Percentage	42.1%	57.9%			
Middle	Top	Win	Frequency	15	26	2.095	0.148	−0.159
			Percentage	36.6%	63.4%			
		Lose	Frequency	22	20			
			Percentage	52.4%	47.6%			
High	Top	Win	Frequency	19	23	0.860	0.354	−0.096
			Percentage	45.2%	54.8%			
		Lose	Frequency	28	23			
			Percentage	54.9%	45.1%			
Low	Corner	Win	Frequency	1	3		1.000 (**)	−0.091
			Percentage	25.0%	75.0%			
		Lose	Frequency	1	2			
			Percentage	33.3%	66.7%			
Middle	Corner	Win	Frequency	23	29	0.008	0.929	−0.009
			Percentage	44.2%	55.8%			
		Lose	Frequency	23	28			
			Percentage	45.1%	54.9%			
High	Corner	Win	Frequency	31	43	0.019	0.891	−0.013
			Percentage	41.9%	58.1%			
		Lose	Frequency	19	25			
			Percentage	43.2%	56.8%			
Low	Wing	Win	Frequency	1	0			
			Percentage	100.0%	0.0%			
		Lose	Frequency	2	0			
			Percentage	100.0%	0.0%			
Middle	Wing	Win	Frequency	32	42	0.007	0.934	−0.007
			Percentage	43.2%	56.8%			
		Lose	Frequency	29	37			
			Percentage	43.9%	56.1%			
High	Wing	Win	Frequency	36	48	0.054	0.817	−0.017
			Percentage	42.9%	57.1%			
		Lose	Frequency	45	56			
			Percentage	44.6%	55.4%			
Low	3P	Win	Frequency	1	1		1.000 (**)	0.167
			Percentage	50.0%	50.0%			
		Lose	Frequency	1	2			
			Percentage	33.3%	66.7%			
Middle	3P	Win	Frequency	28	24	2.953	0.086	0.162
			Percentage	53.8%	46.2%			
		Lose	Frequency	23	38			
			Percentage	37.7%	62.3%			
High	3P	Win	Frequency	18	19	3.884*	0.049	0.208
			Percentage	48.6%	51.4%			
		Lose	Frequency	15	38			
			Percentage	28.3%	71.7%			
Passer	Type of screen			Success	Fail	χ^2^ (df = 1)	*p*	*φ*
Low	On-the-ball screen	Win	Frequency	0	6		0.066 (**)	−0.408
			Percentage	0.0%	100.0%			
		Lose	Frequency	8	10			
			Percentage	44.4%	55.6%			
Middle	On-the-ball screen	Win	Frequency	64	82	0.048	0.826	−0.013
			Percentage	43.8%	56.2%			
		Lose	Frequency	69	84			
			Percentage	45.1%	54.9%			
High	On-the-ball screen	Win	Frequency	77	109	0.235	0.628	−0.026
			Percentage	41.4%	58.6%			
		Lose	Frequency	76	97			
			Percentage	43.9%	56.1%			
Low	Off-the-ball screen	Win	Frequency	8	8	0.259	0.611	0.089
			Percentage	50.0%	50.0%			
		Lose	Frequency	7	10			
			Percentage	41.2%	58.8%			
Middle	Off-the-ball screen	Win	Frequency	57	61	0.138	0.711	0.025
			Percentage	48.3%	51.7%			
		Lose	Frequency	43	51			
			Percentage	45.7%	54.3%			
High	Off-the-ball screen	Win	Frequency	56	54	4.407*	0.036	0.138
			Percentage	50.9%	49.1%			
		Lose	Frequency	45	76			
			Percentage	37.2%	62.8%			

Passer	Movement of off-the- ball screen plays			Success	Fail	χ^2^ (df = 1)	*p*	*φ*
Low	Back	Win	Frequency	3	1		1.000 (**)	0.258
			Percentage	75.0%	25.0%			
		Lose	Frequency	2	2			
			Percentage	50.0%	50.0%			
Middle	Back	Win	Frequency	16	10	4.464*	0.035	0.296
			Percentage	61.5%	38.5%			
		Lose	Frequency	8	17			
			Percentage	32.0%	68.0%			
High	Back	Win	Frequency	10	12	0.155	0.694	0.055
			Percentage	45.5%	54.5%			
		Lose	Frequency	12	18			
			Percentage	40.0%	60.0%			
Low	Cross	Win	Frequency	1	1		0.400 (**)	0.612
			Percentage	50.0%	50.0%			
		Lose	Frequency	0	3			
			Percentage	0.0%	100.0%			
Middle	Cross	Win	Frequency	7	4		0.338 (**)	−0.268
			Percentage	63.6%	36.4%			
		Lose	Frequency	7	1			
			Percentage	87.5%	12.5%			
High	Cross	Win	Frequency	9	3		0.667 (**)	0.177
			Percentage	75.0%	25.0%			
		Lose	Frequency	7	5			
			Percentage	58.3%	41.7%			
Low	Down	Win	Frequency	0	2		0.400 (**)	−0.707
			Percentage	0.0%	100.0%			
		Lose	Frequency	3	1			
			Percentage	75.0%	25.0%			
Middle	Down	Win	Frequency	15	12	3.495	0.062	0.270
			Percentage	55.6%	44.4%			
		Lose	Frequency	6	15			
			Percentage	28.6%	71.4%			
High	Down	Win	Frequency	12	13	3.989*	0.046	0.267
			Percentage	48.0%	52.0%			
		Lose	Frequency	7	24			
			Percentage	22.6%	77.4%			
Low	Flare	Win	Frequency	4	4		0.627 (**)	0.167
			Percentage	50.0%	50.0%			
		Lose	Frequency	2	4			
			Percentage	33.3%	66.7%			
Middle	Flare	Win	Frequency	18	35	4.116*	0.042	−0.210
			Percentage	34.0%	66.0%			
		Lose	Frequency	22	18			
			Percentage	55.0%	45.0%			
High	Flare	Win	Frequency	25	26	0.892	0.345	0.095
			Percentage	49.0%	51.0%			
		Lose	Frequency	19	29			
			Percentage	39.6%	60.4%			

PC, player classification; Low, low-point classification; Middle, middle-point classification; High, high-point classification; PL, paint-low; PH, paint-high; 3P, 3-point field goal area; Back, plays where the screener was on the end-line side of the defense who protected the user; Cross, plays where the screener was on the middle-line (the imaginary line connecting baskets running through the center of the court) side of the defense who protected the user; Down, plays where the screener on the center-line side of the defense who protected the user; Flare, plays where the screener on the side-line side of the defense who protected the user.

* *p* < 0.05.

** We adopted *p*-value by Fisher's method.

### 3.1 Differences in the appearance frequency and shot-success rate of each categorical variable in screen-play between winning and losing teams

Except for categorical variables related to PC, a comparison of the appearance frequency of the winning and losing teams confirmed a significant difference for the following two categories (Table 1): (Ⅳ) the screen location [χ^2^ (5) = 22.483, *p* < 0.001] and (V) pass location [χ^2^ (5) = 14.242, *p* = 0.014]. No significant differences were observed for other categories.

Regarding screen location, the appearance frequency in the PH was higher than expected in the winning team (ASR = 2.330), and lower than expected in the losing team (ASR = −2.330), while the appearance frequency in the Top was higher than expected in the losing team (ASR = 3.739), and lower than expected in the winning team (ASR = −3.739). Regarding pass location, the appearance frequency in the Corner was higher than expected in the winning team (ASR = 2.278), and lower than expected in the losing team (ASR = −2.278), while the appearance frequency in the 3P was higher than expected in the losing team (ASR = 2.073), and lower than expected in the winning team (ASR = −2.073).

Next, a significant difference was found in the shot-success rates of the winning and losing teams for five categories (Table 2): (Ⅱ) presence of a screen, (Ⅲ) shot location, (Ⅴ) pass location, (Ⅶ) type of screen-play, and (Ⅸ) movement of off-the-ball screen plays. No significant differences were observed for other categories.

Specifically, regarding the presence of a screen, the winning team (44.1%) had a significantly higher shot-success rate than the losing team (39.2%) (χ^2^ = 4.469, *p* = 0.035). Regarding shot location, in the plays where shots were delivered from the PH and 3P, the winning team (50.0% and 31.5%, respectively) had a significantly higher shot-success rate than the losing team (26.6% and 20.3%, respectively) (χ^2^ = 8.902, *p* = 0.003; χ^2^ = 4.275, *p* = 0.039, respectively). Regarding pass location, in the plays where the shooter received a pass from 3P, the winning team (51.6%) had a significantly higher shot-success rate than the losing team (33.3%) (χ^2^ = 7.080, *p* = 0.008). Regarding type of screen-play, for ON-U, the winning team (42.8%) had a significantly higher shot-success rate than the losing team (32.7%) (χ^2^ = 7.209, *p* = 0.007). Regarding movement of off-the-ball screen plays, in a Down movement of off-the-ball screen plays, the winning team (50.0%) had a significantly higher shot-success rate than the losing team (28.6%) (χ^2^ = 5.302, *p* = 0.021).

### 3.2 Differences in the appearance frequency and shot-success rate for each categorical variable between winning and losing teams, depending on the PC

#### 3.2.1 Differences in the appearance frequency

Regarding the PC, a significant difference was found between the appearance frequency of the winning and losing teams for one category (Table 3): (Ⅻ) the PC of the screener [χ^2^ (2) = 10.546, *p* = 0.005]. No significant differences were observed for other categories.

The appearance frequency in the Low screener was higher than expected in the losing team (ASR = 3.174), and lower than expected in the winning team (ASR = −3.174).

#### 3.2.2 PC of the shooter

Regarding the PC of the shooter on screen plays, a significant difference was confirmed in the shot-success rate between the winning and losing teams for nine categories (Table 4): (Ⅱ) presence of a screen, (Ⅲ) shot location, (Ⅳ) screen location, (Ⅴ) pass location, (Ⅵ) type of screen, (Ⅶ) type of screen-play, (Ⅷ) movement of on-the-ball screen plays, (Ⅺ) PC of the user, and (Ⅻ) PC of the screener. No significant differences were observed for other categories.

Regarding presence of a screen, when High players attempted shots on screen plays, the winning team (47.0%) had a significantly higher shot-success rate than the losing team (37.2%) (χ^2^ = 8.323, *p* = 0.004). Regarding shot location, when High players shot at the PH and 3P, the winning team (53.3% and 33.8%, respectively) had a significantly higher shot-success rate than the losing team (19.4% and 19.8%, respectively) (χ^2^ = 9.723, *p* = 0.002 and χ^2^ = 3.902, *p* = 0.048, respectively). Regarding screen location, when High players shot using a screen set on the Wing, the winning team (42.1%) had a significantly higher shot-success rate than the losing team (30.1%) (χ^2^ = 5.169, *p* = 0.023). However, when Low players attempted a shot using a screen set on the Wing, the winning team (21.1%) had a significantly lower shot-success rate than the losing team (53.8%) (χ^2^ = 5.602, *p* = 0.018). Regarding pass location, when Middle players received a pass from the Top and attempted a shot, the winning team (35.6%) had a significantly lower shot-success rate than the losing team (58.5%) (χ^2^ = 5.129, *p* = 0.024). Regarding type of screen, when High players shot using the on-the-ball screen and off-the-ball screen, the winning team (44.4% and 53.7%, respectively) had a significantly higher shot-success rate than the losing team (35.9% and 40.5%, respectively) (χ^2^ = 4.621, *p* = 0.032 and χ^2^ = 4.032, *p* = 0.045, respectively). Regarding type of screen-play, when High players selected ON-U, in the plays where the user attempted the shot as the shooter, the winning team (46.9%) had a significantly higher shot-success rate than the losing team (33.7%) (χ^2^ = 6.638, *p* = 0.010). Regarding movement of on-the-ball screen plays, in plays where High players made a shot after the user used the movement toward the center-line side, the winning team (49.6%) had a significantly higher shot-success rate than the losing team (36.0%) (χ^2^ = 4.722, *p* = 0.030). Regarding PC of the user, in plays where both the shooter and user were High players, the winning team (47.7%) had a significantly higher shot-success rate than the losing team (36.9%) (χ^2^ = 8.304, *p* = 0.004). Regarding PC of the screener, in plays where the shooter was a High player and the screener was a Middle player, the winning team (47.5%) had a significantly higher shot-success rate than the losing team (35.5%) (χ^2^ = 5.503, *p* = 0.019).

#### 3.2.3 PC of the user

Regarding the PC of the user on screen plays, we confirmed a significant difference in the shot-success rate between the winning and losing teams for five categories (Table 5): (Ⅲ) shot location, (V) pass location, (Ⅵ) type of screen, (Ⅶ) type of screen-play, (Ⅸ) movement of off-the-ball screen plays. No significant differences were observed for other categories. Since ON-U is a play where the user attempts the shot as the shooter, the result of analyzing ON-U according to (Ⅶ) type of screen-play on this section is similar to that based on the PC of the shooter.

Regarding shot location, in the plays where the user was a High player and the shooter shot at the PH and 3P, the winning team (51.1% and 36.2%, respectively) had a significantly higher shot-success rate than the losing team (24.3% and 19.8%, respectively) (χ^2^ = 6.206, *p* = 0.013 and χ^2^ = 5.253, *p* = 0.022, respectively). Regarding pass location, in plays where the user was a High player and the shooter received a pass from the 3P immediately before the shot, the winning team (52.7%) had a significantly higher shot-success rate than the losing team (34.4%) (χ^2^ = 4.068, *p* = 0.044). Regarding type of screen, in plays where the shooter shot after a High user used the off-the-ball screen, the winning team (52.0%) had a significantly higher shot-success rate than the losing team (38.8%) (χ^2^ = 4.218, *p* = 0.040). Regarding movement of off-the-ball screen plays, in plays where the shooter shot after a Middle user used a Down movement, the winning team (52.2%) had a significantly higher shot-success rate than the losing team (24.0%) (χ^2^ = 4.057, *p* = 0.044).

#### 3.2.4 PC of the screener

We confirmed a significant difference in the shot-success rates of the winning and losing teams according to the PC of the screener for three categories (Table 6): (Ⅲ) shot location, (Ⅴ) pass location, (Ⅶ) type of screen-play. No significant differences were observed for other categories.

Regarding shot location, in the plays where the screener was a Low player and the shooter shot at the PH, the winning team (55.0%) had a significantly higher shot-success rate than the losing team (12.0%) (χ^2^ = 9.586, *p* = 0.002). In plays involving a Middle screener and where the shooter shot at the 3P, the winning team (38.8%) had a significantly higher shot-success rate than the losing team (16.2%) (χ^2^ = 8.686, *p* = 0.003). In plays where the screener was a High player and the shooter shot at the PH and Corner, the winning team (75.0% and 56.0%, respectively) had a significantly higher shot-success rate than the losing team (30.0% and 33.3%, respectively) (χ^2^ = 7.200, *p* = 0.007 and χ^2^ = 4.322, *p* = 0.038, respectively). Regarding pass location, in plays involving a Middle screener and where the shooter shot after receiving a pass from the 3P, the winning team (60.9%) had a significantly higher shot-success rate than the losing team (34.0%) (χ^2^ = 6.712, *p* = 0.010). However, in plays involving a Middle screener and where the shooter shot after receiving a pass from the Top, the winning team (31.0%) had a significantly lower shot-success rate than the losing team (54.7%) (χ^2^ = 5.365, *p* = 0.021). Regarding type of screen-play, in plays involving a Low screener and where the shooter shot by ON-U, the winning team (52.5%) had a significantly higher shot-success rate than the losing team (31.2%) (χ^2^ = 6.398, *p* = 0.011).

#### 3.2.5 PC of the passer

Regarding the PC of the passer on screen plays, we confirmed a significant difference in the shot-success rates of the winning and losing teams for four categories (Table 7): (Ⅲ) shot location, (Ⅴ) pass location, (Ⅵ) type of screen, (Ⅸ) movement of off-the-ball screen plays. No significant differences were observed for other categories.

Regarding shot location, in the plays where the passer was a Middle player and the shooter shot at the 3P, the winning team (42.4%) had a significantly higher shot-success rate than the losing team (4.8%) (χ^2^ = 9.074, *p* = 0.003). When the passer was a High player and the shooter shot at the Wing, the winning team (45.2%) had a significantly higher shot-success rate than the losing team (18.8%) (χ^2^ = 5.696, *p* = 0.017). Regarding pass location, in the plays where the passer was a High player and the shooter shot after receiving a pass from the 3P, the winning team (48.6%) had a significantly higher shot-success rate than the losing team (28.3%) (χ^2^ = 3.884, *p* = 0.049). Regarding type of screen, when the passer was a High player and the shooter shot after the user used the off-the-ball screen, the winning team (50.9%) had a significantly higher shot-success rate than the losing team (37.2%) (χ^2^ = 4.407, *p* = 0.036). Regarding movement of off-the-ball screen plays, when the passer was a Middle player and the shooter shot after the user used a Back movement, the shot-success rate was significantly higher in the winning team (61.5%) than in the losing team (32.0%) (χ^2^ = 4.464, *p* = 0.035). However, when the passer was a Middle player and the shooter shot after the user used a Flare movement, the winning team (34.0%) had a significantly lower shot-success rate than the losing team (55.0%) (χ^2^ = 4.116, *p* = 0.042). In the plays where the passer was a High player and the shooter shot after the user used a Down movement, the shot-success rate of the winning team (48.0%) was significantly higher than that of the losing team (22.6%) (χ^2^ = 3.989, *p* = 0.046).

## 4 Discussion

### 4.1 Differences in play style between the winning and losing teams in screen-play

The collective impact of different screen-play types creates distinct play styles between the winning and losing teams. The winning teams strategically positioned their screens in the central area of the court (relatively close to the basket) for offense, as they appeared significantly more frequently at the PH area than at the Top in terms of screen location. Additionally, for offensive passing, the winning teams predominantly utilized the side-line position (relatively close to the end-line) of the court for offense, as they appeared significantly more frequently in the Corner location than in the 3P in terms of pass location. Research of on-the-ball screen plays focusing on a running basketball team that finished runners-up in the World Championship men's games in 2006 indicates that the team tended to set a screen in the central location of the court, not the side-line side (22). However, the team tended to set their screens more in “the high court area”, a location far from the basket. Given that the winning teams set more screens at the PH, closer to the basket, there may be differences in the effective locations for setting screens between wheelchair basketball and running basketball. Furthermore, a previous study has reported that setting a screen on the side-line side is more effective in enabling users to progress toward the end-line side (12). The critical point of view common to both is using screen plays to create free space (16, 23). Therefore, although significant differences were not confirmed regarding screen plays on the side-line side (Corner, Wing), the winning team may have set screens in appropriate locations and used the free space effectively created by screen plays, as recommended in running basketball.

Considering the result of presence of a screen, the winning team used screen-play effectively to score. Regarding the shot location, the winning team made shots at a higher success rate than the losing team, not only at a location relatively close to the basket, such as PH, but also at a location far from the basket, such as 3P. Given that there is a higher tendency for more shots to be taken in the paint than in other locations in wheelchair basketball (24), it is necessary to consider the practical use of screen plays for shooting at a location close to the basket to make shots with a high success rate. However, it is also necessary to consider the practical use of screen plays for shooting at a location far from the basket due to the difference in the points obtained per shot (i.e., 2-point vs. 3-point shots). Specifically, when considering the practical use of screen plays as an offensive tactic, if there are few defenders around the basket and it is possible to penetrate there, aiming for the shot relatively close to the basket is effective in terms of shot-success rate. On the other hand, if there are many defenders around the basket and a higher shot-success rate cannot be expected, aiming for the shot at 3P is effective in terms of scoring efficiency. In recent trends in running basketball, the number of 3-point shots and shots in the paint has been rising, while the number of midrange 2-point shots has been decreasing (15, 25). The number of shots at 3P is not large in wheelchair basketball screen plays; in this study, the proportion of these shots out of the total number of shots was 13.2% (124/936) and 15.7% (138/877) for the winning and losing teams, respectively. However, increasing the number of shots at 3P may be effective, as in running basketball. This is because having the ball player farther from the basket can lure the defense away from the basket. Luring the defense may allow the ball player to easily get past the defense if the distance to the player is short and the time lag is very short (23). Moreover, there is no “double dribble” violation in wheelchair basketball, which is different from running basketball (10, 17). Therefore, for attacking near the basket in wheelchair basketball, it may also be effective to create situations where players can shoot at a 3P, luring the defense further away from the basket. Regarding the screen location, considering above results (difference between the PH and Top), in wheelchair basketball, a location relatively close to the basket is an important space that should be used not only as a location to shoot but also as a location to set a screen. Additionally, regarding the pass location, the above difference in the shot-success rate at the 3P likely affected the finding in plays where a pass was issued from the 3P to the shooter. We believe this finding might have been a direct consequence of defenses focusing on players with good shooting ability, which could create a greater possibility for other players to shoot. However, further research may be needed to verify this notion. Considering these points, when using screen plays practically in wheelchair basketball, it is essential to use the following locations to shoot, set screens, and pass: one closer to the basket in the paint and one farther from the basket at 3P.

Regarding the type of screen-play, we found that the winning team showed a higher ON-U shot accuracy. Studies on screen-play in running basketball have verified the effectiveness of a tactic, where the ball handler was the user, known as Pick & Roll (12, 16, 22, 23, 26, 27). In this study, approximately 96.4% (612/635) of screen plays using the on-the-ball screen targeted for this analysis corresponded to the Pick & Roll tactic. Some of these studies have reported that users' shots employing the Pick & Roll maneuver are effective (12, 23). However, some of these studies have also reported that the plays where the user passes after using the screen are efficient (16, 22, 27). Although the number was smaller than that of ON-U (winning team: 154/360, 42.8%; losing team: 102/312, 32.7%), the result of this study indicated that ON-S had a higher shot-success rate for both winning and losing teams (31/69, 44.9% and 40/70, 57.1%, respectively). Therefore, improving the accuracy of the Pick & Roll maneuver is crucial in wheelchair basketball, as in running basketball. Specifically, it is essential in screen-play to suppose various situations in which the user has difficulty shooting and practice repeatedly so that players can make the more appropriate choice (e.g., who to pass to). The number of plays that led to a successful shot using the on-the-ball screen (541) was significantly higher than that of those that led to a shot using the off-the-ball screen (216) (χ^2^ = 139.531, *p* < 0.001). However, since the shot-success rate in a Down movement was significantly higher for the winning team than the losing team, the winning team effectively used the off-the-ball screen plays. Furthermore, just as the off-the-ball screen should be used for purposes other than shooting in running basketball (26), it may also be necessary to consider using it for purposes other than shooting in wheelchair basketball; hence, the practical use of the off-the-ball screen should not be overlooked.

### 4.2 Relationship between screen-play and the PC

This study's result shows the losing team more typically assigned Low players to screeners than the winning team. Additionally, no significant difference was observed in the frequency of Low and High screeners (237/919, 25.8% and 225/919, 24.5%, respectively) in the winning team (χ^2^ = 0.312, *p* = 0.577), while a significant difference was found in this frequency (284/871, 32.6% and 183/871, 21.0%, respectively) in the losing team (χ^2^ = 21.844, *p* < 0.001). In the traditional wheelchair basketball offense, “the low pointers” are encouraged to work to set the screen to get “the high pointers” free for high-percentage shots (14). Although it is possible that “the low pointers” and “the high pointers” do not entirely match our division of Low and High, the results from the present study show that the winning team adopted a different strategy from this encouragement. Therefore, it is necessary to consider the balance with other players rather than simply fixing Low players in the role of screeners. Considering the result of differences in the appearance frequency regarding the screener depending on the PC, a practical solution may be to assign approximately 50% of screener roles to Middle players, and to balance the remainder between Low and High players. In wheelchair basketball, to represent the roles of each player with different PCs, Low, Middle, and High players are frequently categorized as guards, forwards, and centers, respectively, corresponding to running basketball positions (5, 24). In running basketball screen-play, it is more effective for the center to play the role of screener (12). Therefore, this result shows the difference in player roles in wheelchair and running basketball.

Regarding differences in the shot-success rate according to the PC of the shooter, High players primarily contributed to the winning team's successful plays. This shows a tendency similar to that reported in previous studies in wheelchair basketball (8, 24, 28). Additionally, the various results that showed a significant difference only in High players between the winning and losing teams indicate High players' contributions to screen-play. High players' contributions as shooters can be confirmed by the following results: (Ⅱ) presence of a screen (in plays with screen), (Ⅲ) shot location (in plays where shot at PH and 3P), (Ⅳ) screen location (in plays where screens set at Wing), (Ⅵ) type of screen (in plays with the on-the-ball and off-the-ball screen plays), (Ⅶ) type of screen-play (in plays where used ON-U), (Ⅷ) movement of on-the-ball screen plays (in plays where used Center-line movement), (Ⅺ) PC of the user (in plays where the users were High), and (Ⅻ) PC of the screener (in plays where the screeners were Middle). The results described in the previous section were directly influenced by whether the shooter was a High player: the winning team had a higher shot-success rate than the losing team when shooting at the PH, which is relatively close to the basket, and at 3P, which is far from the basket. However, regarding the results of screen and pass locations, when the shooter was a Low or Middle player, the shot-success rate of the losing team was higher than that of the winning team. This result is highly likely related to the findings of Gil et al. (29), who reported that differences in strength of the trunk muscles and pelvic stability due to classification affected the distance when throwing or passing a ball. Furthermore, the higher PC players tended to have a higher sitting body height (29). Therefore, their ball-release position was higher than that of lower PC players, and the ball could reach the ring even with a lower release velocity (30, 31). Thus, creating a situation where a High player can shoot is more important for ensuring a high success rate. In addition, considering the result of the type of screen, the High shooters of the winning team could use the on-the-ball screen and off-the-ball screen practically without distinction for screen plays. However, we found a significant difference in the type of screen-play only for ON-U. Thus, when the shooter is a High player, it is possible that the practical use of the on-the-ball screen, which occurs more frequently, has a more substantial effect on the outcome than that of the off-the-ball screen. Moreover, considering that setting screens to facilitate users' movement toward the central location of the court in running basketball is effective (12), the High player's Center-line movement of on-the-ball screen plays may have been more effective.

For the results of PH and 3P in shot location and the off-the-ball screen in the type of screen regarding the PC of the user, the High players of the winning team recorded a higher shot-success rate, and these results are almost identical to those regarding the shooter. Moreover, 52.0% (360/692) of the on-the-ball screen plays were ON-U, and 92.6% (226/244) of the off-the-ball screen plays were OF-U in the winning team. Since both categories refer to plays in which the user takes a shot, the user may have contributed to the winning team's success, mainly as the shooter. However, considering the result according to 3P in the pass location of the PC of the passer, it may be effective for the user to become the passer instead of the shooter. In other words, not only did the High players of the winning team make high-percentage 3-point shots, but they also made passes at 3P that led to high-percentage shots. On the other hand, in addition to the High players, other classification players also contributed as users to the team's success in the winning team. The winning team had a higher shot-success rate in a Down movement of off-the-ball screen plays than the losing team, and this result is related to the contributions of the Middle players as users. Therefore, since Middle players contribute significantly as users, it is impractical to use only High players as users.

Considering the results of PH in shot location and ON-U in the type of screen-play regarding the PC of the screener, the Low players contributed to the winning team's successful plays executing their roles as screeners. In addition, the winning team's Middle players contributed as screeners in plays where shots and passes were made at 3P, and High players contributed as screeners in plays where shots were made at PH and Corner. The result of the Low screener in the winning team may indicate the Low players' contribution in the plays mentioned so far where shots were made at PH (as shown in Table 2). Similarly, in the winning team, Middle screeners may have contributed in plays where shots were made at 3P, and High screeners may have contributed in plays where shots were made at PH. Therefore, although at different frequencies, Low, Middle, and High players contributed as screeners. Furthermore, the High players of the winning team contributed as screeners in plays where shots were made at the Corner, a priority location in wheelchair basketball for shooting a field goal in the study of Francis et al. (8). Additionally, as mentioned above, the winning team utilized the Corner as the pass location more than the losing team. Thus, our findings might indicate that the winning team follows the theory for using screen-play. In addition, in the winning team, the Middle screeners' contributions in plays where the shooter received a pass from the 3P may be related to the results of the pass location of High passers described in the previous section (as shown in Table 7). Low players in the winning team contributed as screeners to ON-U, whose importance we have previously described. In other words, High player's successful performance in screen-play as a shooter is only possible thanks to the contributions of Middle and Low players. Therefore, when practicing screen plays in wheelchair basketball, it is vital to consider the actions of Middle and Low players in the screener's role, creating a situation where the shooter can shoot with a high success rate. However, considering the result of the screener in pass location, a difference in the tendency to make practical use of screen plays between the winning and losing teams needs to be considered, even if the screener was a Middle player.

Regarding the difference in the shot-success rate from the perspective of the PC of the passer, we found that Middle and High players contributed to the winning team's success. The winning team succeeded in plays where the shooter received a pass from a Middle player and shot at 3P. Thus, Middle players contributed not only as screeners but also as passers. Additionally, in plays where the shooter shot after receiving a pass from 3P, the winning team had a higher shot-success rate when the passer was a High player. Therefore, plays might be constructed more effectively by considering the results of the PC of the shooter regarding the shot location and the PC of the user regarding the pass location, and the results of the PC of the passer additionally. For instance, if the screen user with good shooting ability holds the ball far from the basket, the defense is forced to respond to prevent the user from shooting. By creating such a situation where other players can receive the pass and shoot with a high success rate and where the user can also contribute as a passer, the offense is highly likely to construct the play effectively. Since a play with an off-the-ball screen always involves a pass, the results of type of screen and movement of off-the-ball screen plays must be considered. In the winning team's off-the-ball screen plays, a High passer contributed to the successful plays, especially those with a Down movement. On the other hand, in the plays where a Middle player was the passer, the fact that the winning team had a higher shot-success rate on the Back than on the Flare movement of off-the-ball screen plays compared to losing team indicates a difference in the tendency to make practical use of screen plays between the winning and losing teams. Moreover, these results may be related to the results of the PC of the screener regarding the pass location. Therefore, the passer should understand the type of movement that is appropriate for the screen and the location from which to pass.

Thus, this study's results clearly showed that High players make a direct and significant contribution to scoring, similar to those of previous studies (8, 24, 28). However, it is also clear that Low and Middle players in the winning team played roles as screeners and passers and contributed to the success of screen plays. Therefore, as in the report of Hindawi et al. (32), since players in highly competitive teams may have a high level of thinking and understanding of offensive tactics, it is necessary to understand the tendency in screen plays regardless of the PC. Furthermore, it is necessary to consider that the sum of the PC of the five players playing on the court in wheelchair basketball is limited to 14.0. Therefore, it is essential to pay more attention to the contribution of each of the players with different PCs in each process leading up to the shot in screen-play as offensive tactics in wheelchair basketball.

### 4.3 Practical application

**Figure 5:**
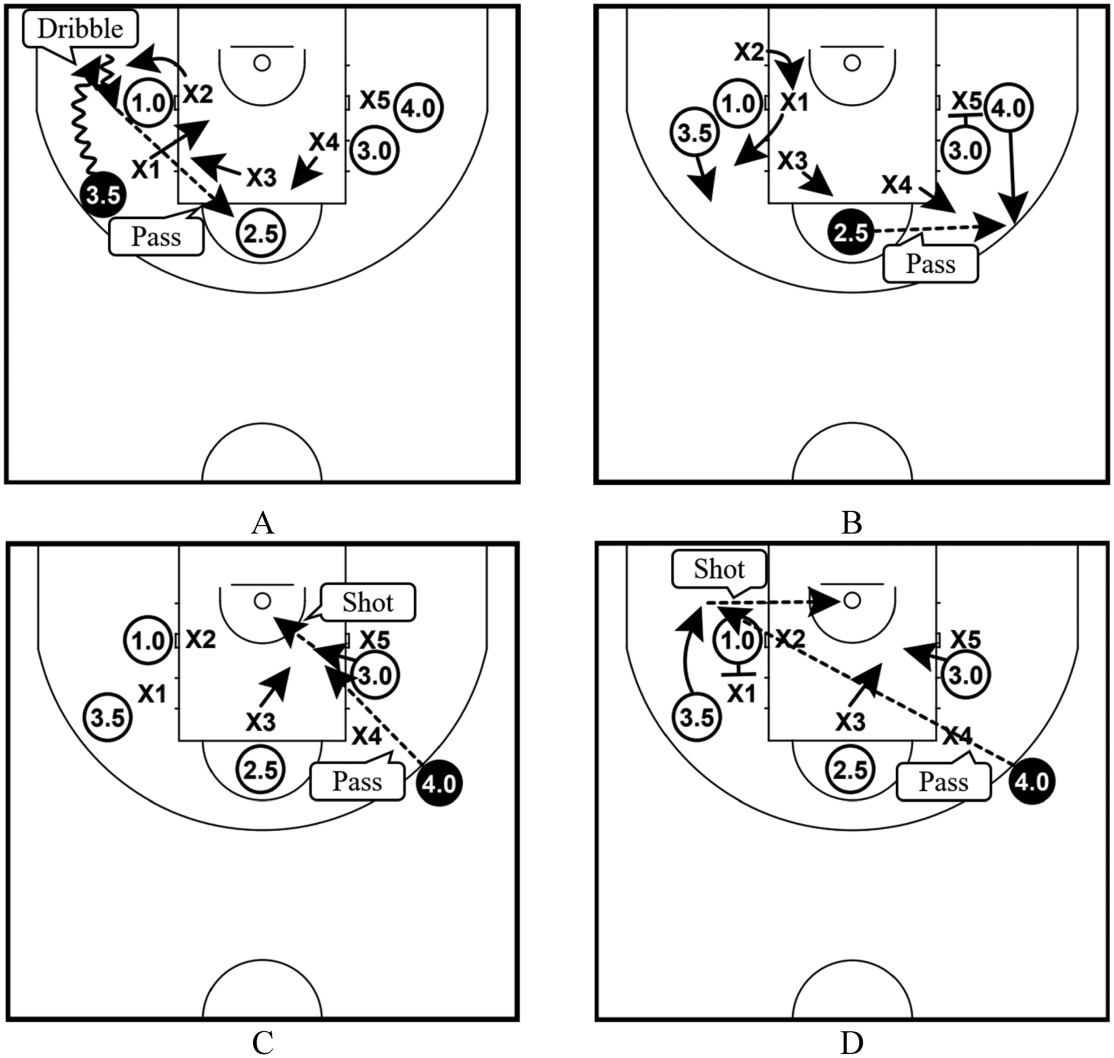
Example of practical application; **(A)** A high user uses the screen of a Low screener in the center-line movement. Against the play, a defense tries to prevent a shot of ON-U. The High user makes a pass to the teammate at the Top; **(B)** A defense tries to prevent a shot at the Top. The Middle player receiving the pass on Top makes a pass to the High player, who moves to the 3P using the Down movement of off-the-ball screen plays; **(C)** the High player receiving the pass at 3P makes a pass to the Middle screener, who dives close to the basket; **(D)** the High player receiving the pass at 3P makes a pass to the High user, who uses the off-the-ball screen from a Back movement.

Based on the above considerations, the following plays may hold practical value (Figure 5): when a High dribbles toward the Corner, and uses the screen of a Low screener in the Center-line movement of on-the-ball screen plays, the defense may attempt to prevent a shot of ON-U, allowing this High user to make a pass to a teammate at the Top (Figure 5A). If a defense attempts to prevent a shot at the Top further, the player receiving the pass can make another pass to the High player, who moves to the 3P using the Down movement of off-the-ball screen plays (Figure 5B). If the High player receiving a pass is expected to have a high shot-success rate at 3P, this play would be more effective. This is because the High player holding a ball may have several options to make a more effective pass besides shooting from the 3P at this time (Figures 5C,D). Thus, effective options would change depending on how a defense reacts to the movement of screen plays.

### 4.4 Limitations

Our study had some limitations. First, a single observer extracted data in this study. However, we did not evaluate intra-rater reliability, which is important to ensure consistency during video analysis (33). Therefore, the actual reliability of video analysis in this study is unknown, which could have affected the quality of data extraction. Second, although we recorded 3,841 possessions, and the results of this study show a tendency similar to that reported in previous studies, the method of recording by a single observer may have increased the possibility of data recording errors in addition to the observer bias. Third, since this study primarily focused on the success rate of shots about screen-play, other critical aspects of wheelchair basketball gameplay, such as turnovers, assists, or defensive actions, may have been overlooked. Furthermore, since the results obtained in this study are limited to the men's games in the Tokyo Paralympics, some variables may have been affected by other competitions and categories. Doi et al. revealed differences between men's and women's teams regarding how offensive rebounds, number of successful field goals, steals, and turnovers affect the team's total scores (28, 34). Therefore, the limited focus on elite men's games may restrict the broader relevance and applicability of the study findings. Additionally, despite the findings that passing to a screener, who moves close to the basket, is effective in running basketball (16, 22, 27), the results regarding the type of screen-play in this study could have shown limited evidence except for the direct effects of the specific on-the-ball screen play (ON-U). Thus, there may be a discrepancy in recognizing the teaching practice. In the future, it will be necessary to conduct surveys and construct new analytical frameworks to overcome these issues.

## 5 Conclusion

In wheelchair basketball offenses, it may be effective to consider the following points in the scenario lead-up to a shot: Regarding the shot, screen, and pass, it could be necessary to use screen plays practically in two different spaces (in the paint and at the 3P). Moreover, it appears vital to improve the on-the-ball screen plays' accuracy, particularly ON-U, which is equivalent to a Pick & Roll maneuver that is also effective in running basketball. Furthermore, using both the on-the-ball screen and off-the-ball screen seems to be a factor in winning the game. In wheelchair basketball screen plays, it may be practical to allocate approximately 50% of the screener roles to Middle players and the rest to Low and High players, at approximately 25% each. Regarding the PC, to win the game, High players should play the roles of shooters and users; Low, Middle, and High players should act as screeners; and Middle and High players should play the roles of passers to contribute to the success of plays. Players expected to have a high shot-success rate may be able to contribute to screen plays more effectively as passers by understanding effective movement and passing options of screen plays. In wheelchair basketball screen-play, further research on the contributions of each player based on different PCs is essential.

## Data Availability

The original contributions presented in the study are included in the article/Supplementary Material, further inquiries can be directed to the corresponding author.
