# Supplementary material for: Relationship between screen-play scenarios' effectiveness and player classification in elite wheelchair basketball based on match results of Tokyo 2020 Paralympic Games
Source: Front Sports Act Living. 2024 Sep 13;6:1418130. doi: 10.3389/fspor.2024.1418130 (PMC11443225; doi:10.3389/fspor.2024.1418130)
Supplement: Supplementary file 1 [file Table1.docx]

Supplementary Material

Relationship between screen-play scenarios’ effectiveness and player classification in elite wheelchair basketball based on match results of Tokyo 2020 Paralympic Games

Taku Yasuda*, Kaori Tachibana, Hirotaka Mutsuzaki

*** Correspondence:** Taku Yasuda: yasudata@ipu.ac.jp

TABLE S1 Frequency distributions in presence of a screen.

|  | **With Screen** | **Without screen** | **Total** |
| --- | --- | --- | --- |
| **Win** | 936 | 375 | 1311 |
| **Lose** | 877 | 379 | 1256 |
| **Total** | 1813 | 754 | 2567 |

TABLE S2 Frequency distributions in shot location.

|  | **PL** | **PH** | **Top** | **Corner** | **Wing** | **3P** | **Total** |
| --- | --- | --- | --- | --- | --- | --- | --- |
| **Win** | 249 | 74 | 91 | 202 | 196 | 124 | 936 |
| **Lose** | 239 | 79 | 82 | 192 | 147 | 138 | 877 |
| **Total** | 488 | 153 | 173 | 394 | 343 | 262 | 1813 |

PL, paint-low; PH, paint-high; 3P, 3-point field goal area.

TABLE S3 Frequency distributions in screen location.

|  | **PL** | **PH** | **Top** | **Corner** | **Wing** | **3P** | **Total** |
| --- | --- | --- | --- | --- | --- | --- | --- |
| **Win** | 53 | 89 | 70 | 260 | 395 | 52 | 919 |
| **Lose** | 55 | 58 | 113 | 228 | 349 | 68 | 871 |
| **Total** | 108 | 147 | 183 | 488 | 744 | 120 | 1790 |

PL, paint-low; PH, paint-high; 3P, 3-point field goal area.

TABLE S4 Frequency distributions in pass location.

|  | **PL** | **PH** | **Top** | **Corner** | **Wing** | **3P** | **Total** |
| --- | --- | --- | --- | --- | --- | --- | --- |
| **Win** | 22 | 85 | 95 | 130 | 159 | 91 | 582 |
| **Lose** | 12 | 68 | 112 | 98 | 169 | 117 | 576 |
| **Total** | 34 | 153 | 207 | 228 | 328 | 208 | 1158 |

PL, paint-low; PH, paint-high; 3P, 3-point field goal area.

TABLE S5 Frequency distributions in type of screen.

|  | **On-the-ball screen** | **Off-the-ball screen** | **Total** |
| --- | --- | --- | --- |
| **Win** | 692 | 244 | 936 |
| **Lose** | 645 | 232 | 877 |
| **Total** | 1337 | 476 | 1813 |

TABLE S6 Frequency distributions in type of screen-play.

|  | **ON-U** | **ON-S** | **ON-A** | **ON-E** | **OF-U** | **OF-S** | **Total** |
| --- | --- | --- | --- | --- | --- | --- | --- |
| **Win** | 360 | 69 | 178 | 85 | 226 | 18 | 936 |
| **Lose** | 312 | 70 | 175 | 88 | 212 | 20 | 877 |
| **Total** | 672 | 139 | 353 | 173 | 438 | 38 | 1813 |

ON-U, the plays where the user shot using the on-the-ball screen; ON-S, plays where the screener of the on-the-ball screen shot after receiving a pass from the user; ON-A, plays where another player shot after receiving a pass from the user of the on-the-ball screen; ON-E, plays that led to a shot through two or more extra passes after the user used the on-the-ball screen; OF-U, plays where the user shot using the off-the-ball screen; OF-S, plays where the screener of the off-the-ball screen shot.

TABLE S7 Frequency distributions in movement of on-the-ball screen plays.

|  | **Around** | **Centre-line** | **End-line** | **ON-Down** | **Total** |
| --- | --- | --- | --- | --- | --- |
| **Win** | 19 | 299 | 347 | 26 | 691 |
| **Lose** | 20 | 285 | 321 | 19 | 645 |
| **Total** | 39 | 584 | 668 | 45 | 1336 |

Around, plays where the screener held a ball; Center-line, plays where the user moved toward the center-line side against the screener; End-line, plays where the user moved toward the end-line side against the screener; ON-Down, plays where the screener was on the center-line side of the defense who protected the user holding a ball in a Top or 3P on Top extension.

TABLE S8 Frequency distributions in movement of off-the-ball screen plays.

|  | **Back** | **Cross** | **Down** | **Flare** | **Total** |
| --- | --- | --- | --- | --- | --- |
| **Win** | 52 | 25 | 54 | 112 | 243 |
| **Lose** | 59 | 23 | 56 | 94 | 232 |
| **Total** | 111 | 48 | 110 | 206 | 475 |

Back, plays where the screener was on the end-line side of the defense who protected the user; Cross, plays where the screener was on the middle-line (the imaginary line connecting goals running through the center of the court) side of the defense who protected the user; Down, plays where the screener on the center-line side of the defense who protected the user; Flare, plays where the screener on the side-line side of the defense who protected the user.

TABLE S9 Difference in the appearance frequency in presence of a screen.

| **Presence of Screen** |  | **Win** | **Lose** | **χ^2^(df=1)** | ***p*** | ***φ*** |
| --- | --- | --- | --- | --- | --- | --- |
| **With Screen** | Frequency | 936 | 877 | 0.763 | 0.382 | 0.017 |
|  | Percentage | 71.4% | 69.8% |  |  |  |
|  | ASR | 0.874 | -0.874 |  |  |  |
| **Without screen** | Frequency | 375 | 379 |  |  |  |
|  | Percentage | 28.6% | 30.2% |  |  |  |
|  | ASR | -0.874 | 0.874 |  |  |  |

ASR, adjusted standardized residual.

TABLE S10 Differences in the appearance frequency in shot location.

| **Shot Location** |  | **Win** | **Lose** | **χ^2^(df=5)** | ***p*** | **Cramér’s V (*φ*_c_)** |
| --- | --- | --- | --- | --- | --- | --- |
| **PL** | Frequency | 249 | 239 | 6.926 | 0.226 | 0.062 |
|  | Percentage | 26.6% | 27.3% |  |  |  |
|  | ASR | -0.312 | 0.312 |  |  |  |
| **PH** | Frequency | 74 | 79 |  |  |  |
|  | Percentage | 7.9% | 9.0% |  |  |  |
|  | ASR | -0.844 | 0.844 |  |  |  |
| **Top** | Frequency | 91 | 82 |  |  |  |
|  | Percentage | 9.7% | 9.4% |  |  |  |
|  | ASR | 0.270 | -0.270 |  |  |  |
| **Corner** | Frequency | 202 | 192 |  |  |  |
|  | Percentage | 21.6% | 21.9% |  |  |  |
|  | ASR | -0.161 | 0.161 |  |  |  |
| **Wing** | Frequency | 196 | 147 |  |  |  |
|  | Percentage | 20.9% | 16.8% |  |  |  |
|  | ASR | 2.270 | -2.270 |  |  |  |
| **3P** | Frequency | 124 | 138 |  |  |  |
|  | Percentage | 13.2% | 15.7% |  |  |  |
|  | ASR | -1.505 | 1.505 |  |  |  |

PL, paint-low; PH, paint-high; 3P, 3-point field goal area; ASR, adjusted standardized residual.

TABLE S11 Differences in the appearance frequency in screen location.

| **Screen Location** |  | **Win** | **Lose** | **χ^2^(df=5)** | ***p*** | **Cramér’s V (*φ*_c_)** |
| --- | --- | --- | --- | --- | --- | --- |
| **PL** | Frequency | 53 | 55 | 22.483* | < 0.001 | 0.112 |
|  | Percentage | 5.8% | 6.3% |  |  |  |
|  | ASR | -0.486 | 0.486 |  |  |  |
| **PH** | Frequency | 89 | 58 |  |  |  |
|  | Percentage | 9.7% | 6.7% |  |  |  |
|  | ASR | 2.330 | -2.330 |  |  |  |
| **Top** | Frequency | 70 | 113 |  |  |  |
|  | Percentage | 7.6% | 13.0% |  |  |  |
|  | ASR | -3.739 | 3.739 |  |  |  |
| **Corner** | Frequency | 260 | 228 |  |  |  |
|  | Percentage | 28.3% | 26.2% |  |  |  |
|  | ASR | 1.004 | -1.004 |  |  |  |
| **Wing** | Frequency | 395 | 349 |  |  |  |
|  | Percentage | 43.0% | 40.1% |  |  |  |
|  | ASR | 1.250 | -1.250 |  |  |  |
| **3P** | Frequency | 52 | 68 |  |  |  |
|  | Percentage | 5.7% | 7.8% |  |  |  |
|  | ASR | -1.817 | 1.817 |  |  |  |

PL, paint-low; PH, paint-high; 3P, 3-point field goal area; ASR, adjusted standardized residual.

**p* < 0.05.

TABLE S12 Differences in the appearance frequency in pass location.

| **Pass Location** |  | **Win** | **Lose** | **χ^2^(df=5)** | ***p*** | **Cramér’s V (*φ*_c_)** |
| --- | --- | --- | --- | --- | --- | --- |
| **PL** | Frequency | 22 | 12 | 14.242* | 0.014 | 0.111 |
|  | Percentage | 3.8% | 2.1% |  |  |  |
|  | ASR | 1.710 | -1.710 |  |  |  |
| **PH** | Frequency | 85 | 68 |  |  |  |
|  | Percentage | 14.6% | 11.8% |  |  |  |
|  | ASR | 1.407 | -1.407 |  |  |  |
| **Top** | Frequency | 95 | 112 |  |  |  |
|  | Percentage | 16.3% | 19.4% |  |  |  |
|  | ASR | -1.386 | 1.386 |  |  |  |
| **Corner** | Frequency | 130 | 98 |  |  |  |
|  | Percentage | 22.3% | 17.0% |  |  |  |
|  | ASR | 2.278 | -2.278 |  |  |  |
| **Wing** | Frequency | 159 | 169 |  |  |  |
|  | Percentage | 27.3% | 29.3% |  |  |  |
|  | ASR | -0.763 | 0.763 |  |  |  |
| **3P** | Frequency | 91 | 117 |  |  |  |
|  | Percentage | 15.6% | 20.3% |  |  |  |
|  | ASR | -2.073 | 2.073 |  |  |  |

PL, paint-low; PH, paint-high; 3P, 3-point field goal area; ASR, adjusted standardized residual.

**p* < 0.05.

TABLE S13 Difference in the appearance frequency in type of screen.

| **Type of Screen** |  | **Win** | **Lose** | **χ^2^(df=1)** | ***p*** | ***φ*** |
| --- | --- | --- | --- | --- | --- | --- |
| **On-the-ball screen** | Frequency | 692 | 645 | 0.035 | 0.852 | 0.004 |
|  | Percentage | 73.9% | 73.5% |  |  |  |
|  | ASR | 0.186 | -0.186 |  |  |  |
| **Off-the-ball screen** | Frequency | 244 | 232 |  |  |  |
|  | Percentage | 26.1% | 26.5% |  |  |  |
|  | ASR | -0.186 | 0.186 |  |  |  |

ASR, adjusted standardized residual.

TABLE S14 Differences in the appearance frequency in type of screen-play.

| **Type of Screen-play** |  | **Win** | **Lose** | **χ^2^(df=5)** | ***p*** | **Cramér’s V (*φ*_c_)** |
| --- | --- | --- | --- | --- | --- | --- |
| **ON-U** | Frequency | 360 | 312 | 2.148 | 0.828 | 0.034 |
|  | Percentage | 38.5% | 35.6% |  |  |  |
|  | ASR | 1.271 | -1.271 |  |  |  |
| **ON-S** | Frequency | 69 | 70 |  |  |  |
|  | Percentage | 7.4% | 8.0% |  |  |  |
|  | ASR | -0.488 | 0.488 |  |  |  |
| **ON-A** | Frequency | 178 | 175 |  |  |  |
|  | Percentage | 19.0% | 20.0% |  |  |  |
|  | ASR | -0.504 | 0.504 |  |  |  |
| **ON-E** | Frequency | 85 | 88 |  |  |  |
|  | Percentage | 9.1% | 10.0% |  |  |  |
|  | ASR | -0.690 | 0.690 |  |  |  |
| **OF-U** | Frequency | 226 | 212 |  |  |  |
|  | Percentage | 24.1% | 24.2% |  |  |  |
|  | ASR | -0.014 | 0.014 |  |  |  |
| **OF-S** | Frequency | 18 | 20 |  |  |  |
|  | Percentage | 1.9% | 2.3% |  |  |  |
|  | ASR | -0.531 | 0.531 |  |  |  |

ON-U, the plays where the user shot using the on-the-ball screen; ON-S, plays where the screener of the on-the-ball screen shot after receiving a pass from the user; ON-A, plays where another player shot after receiving a pass from the user of the on-the-ball screen; ON-E, plays that led to a shot through two or more extra passes after the user used the on-the-ball screen; OF-U, plays where the user shot using the off-the-ball screen; OF-S, plays where the screener of the off-the-ball screen shot; ASR, adjusted standardized residual.

TABLE S15 Differences in the appearance frequency in movement of on-the-ball screen plays.

| **Movement of on-the-ball screen plays** |  | **Win** | **Lose** | **χ^2^(df=3)** | ***p*** | **Cramér’s V (*φ*_c_)** |
| --- | --- | --- | --- | --- | --- | --- |
| **Around** | Frequency | 19 | 20 | 0.879 | 0.830 | 0.026 |
|  | Percentage | 2.7% | 3.1% |  |  |  |
|  | ASR | -0.381 | 0.381 |  |  |  |
| **Centre-line** | Frequency | 299 | 285 |  |  |  |
|  | Percentage | 43.3% | 44.2% |  |  |  |
|  | ASR | -0.337 | 0.337 |  |  |  |
| **End-line** | Frequency | 347 | 321 |  |  |  |
|  | Percentage | 50.2% | 49.8% |  |  |  |
|  | ASR | 0.164 | -0.164 |  |  |  |
| **ON-Down** | Frequency | 26 | 19 |  |  |  |
|  | Percentage | 3.8% | 2.9% |  |  |  |
|  | ASR | 0.827 | -0.827 |  |  |  |

Around, plays where the screener held a ball; Center-line, plays where the user moved toward the center-line side against the screener; End-line, plays where the user moved toward the end-line side against the screener; ON-Down, plays where the screener was on the center-line side of the defense who protected the user holding a ball in a Top or 3P on Top extension; ASR, adjusted standardized residual.

TABLE S16 Differences in the appearance frequency in movement of off-the-ball screen plays.

| **Movement of off-the-ball screen plays** |  | **Win** | **Lose** | **χ^2^(df=3)** | ***p*** | **Cramér’s V (*φ*_c_)** |
| --- | --- | --- | --- | --- | --- | --- |
| **Back** | Frequency | 52 | 59 | 1.880 | 0.598 | 0.063 |
|  | Percentage | 21.4% | 25.4% |  |  |  |
|  | ASR | -1.038 | 1.038 |  |  |  |
| **Cross** | Frequency | 25 | 23 |  |  |  |
|  | Percentage | 10.3% | 9.9% |  |  |  |
|  | ASR | 0.135 | -0.135 |  |  |  |
| **Down** | Frequency | 54 | 56 |  |  |  |
|  | Percentage | 22.2% | 24.1% |  |  |  |
|  | ASR | -0.495 | 0.495 |  |  |  |
| **Flare** | Frequency | 112 | 94 |  |  |  |
|  | Percentage | 46.1% | 40.5% |  |  |  |
|  | ASR | 1.225 | -1.225 |  |  |  |

Back, plays where the screener was on the end-line side of the defense who protected the user; Cross, plays where the screener was on the middle-line (the imaginary line connecting baskets running through the center of the court) side of the defense who protected the user; Down, plays where the screener on the center-line side of the defense who protected the user; Flare, plays where the screener on the side-line side of the defense who protected the user; ASR, adjusted standardized residual.

TABLE S17 Differences in the appearance frequency depending on the PC of the shooter.

| **Shooter** |  | **Win** | **Lose** | **χ^2^(df=2)** | ***p*** | **Cramér’s V (*φ*_c_)** |
| --- | --- | --- | --- | --- | --- | --- |
| **Low** | Frequency | 60 | 79 | 4.354 | 0.113 | 0.049 |
|  | Percentage | 6.4% | 9.0% |  |  |  |
|  | ASR | -2.078 | 2.078 |  |  |  |
| **Middle** | Frequency | 440 | 397 |  |  |  |
|  | Percentage | 47.0% | 45.3% |  |  |  |
|  | ASR | 0.743 | -0.743 |  |  |  |
| **High** | Frequency | 436 | 401 |  |  |  |
|  | Percentage | 46.6% | 45.7% |  |  |  |
|  | ASR | 0.366 | -0.366 |  |  |  |

PC, player classification; Low, low-point classification; Middle, middle-point classification; High, high-point classification; ASR, adjusted standardized residual.

TABLE S18 Differences in the appearance frequency depending on the PC of the user.

| **User** |  | **Win** | **Lose** | **χ^2^(df=2)** | ***p*** | **Cramér’s V (*φ*_c_)** |
| --- | --- | --- | --- | --- | --- | --- |
| **Low** | Frequency | 19 | 25 | 2.081 | 0.353 | 0.034 |
|  | Percentage | 2.0% | 2.9% |  |  |  |
|  | ASR | -1.135 | 1.135 |  |  |  |
| **Middle** | Frequency | 409 | 398 |  |  |  |
|  | Percentage | 43.7% | 45.4% |  |  |  |
|  | ASR | -0.722 | 0.722 |  |  |  |
| **High** | Frequency | 508 | 454 |  |  |  |
|  | Percentage | 54.3% | 51.8% |  |  |  |
|  | ASR | 1.069 | -1.069 |  |  |  |

PC, player classification; Low, low-point classification; Middle, middle-point classification; High, high-point classification; ASR, adjusted standardized residual.

TABLE S19 Differences in the appearance frequency depending on the PC of the screener.

| **Screener** |  | **Win** | **Lose** | **χ^2^(df=2)** | ***p*** | **Cramér’s V (*φ*_c_)** |
| --- | --- | --- | --- | --- | --- | --- |
| **Low** | Frequency | 237 | 284 | 10.546* | 0.005 | 0.077 |
|  | Percentage | 25.8% | 32.6% |  |  |  |
|  | ASR | -3.174 | 3.174 |  |  |  |
| **Middle** | Frequency | 457 | 404 |  |  |  |
|  | Percentage | 49.7% | 46.4% |  |  |  |
|  | ASR | 1.416 | -1.416 |  |  |  |
| **High** | Frequency | 225 | 183 |  |  |  |
|  | Percentage | 24.5% | 21.0% |  |  |  |
|  | ASR | 1.751 | -1.751 |  |  |  |

PC, player classification; Low, low-point classification; Middle, middle-point classification; High, high-point classification; ASR, adjusted standardized residual.

**p* < 0.05.

TABLE S20 Differences in the appearance frequency depending on the PC of the passer.

| **Passer** |  | **Win** | **Lose** | **χ^2^(df=2)** | ***p*** | **Cramér’s V (*φ*_c_)** |
| --- | --- | --- | --- | --- | --- | --- |
| **Low** | Frequency | 22 | 35 | 3.506 | 0.173 | 0.055 |
|  | Percentage | 3.8% | 6.1% |  |  |  |
|  | ASR | -1.806 | 1.806 |  |  |  |
| **Middle** | Frequency | 264 | 247 |  |  |  |
|  | Percentage | 45.4% | 42.9% |  |  |  |
|  | ASR | 0.849 | -0.849 |  |  |  |
| **High** | Frequency | 296 | 294 |  |  |  |
|  | Percentage | 50.9% | 51.0% |  |  |  |
|  | ASR | -0.062 | 0.062 |  |  |  |

PC, player classification; Low, low-point classification; Middle, middle-point classification; High, high-point classification; ASR, adjusted standardized residual.

TABLE S21 Difference in shot-success rate in presence of a screen.

| **Presence of Screen** |  |  | **Success** | **Fail** | **χ^2^(df=1)** | ***p*** | ***φ*** |
| --- | --- | --- | --- | --- | --- | --- | --- |
| **With Screen** | Win | Frequency | 413 | 523 | 4.469* | 0.035 | 0.050 |
|  |  | Percentage | 44.1% | 55.9% |  |  |  |
|  | Lose | Frequency | 344 | 533 |  |  |  |
|  |  | Percentage | 39.2% | 60.8% |  |  |  |
| **Without Screen** | Win | Frequency | 203 | 172 | 3.066 | 0.080 | 0.064 |
|  |  | Percentage | 54.1% | 45.9% |  |  |  |
|  | Lose | Frequency | 181 | 198 |  |  |  |
|  |  | Percentage | 47.8% | 52.2% |  |  |  |

**p* < 0.05.

TABLE S22 Differences in shot-success rate in shot location.

| **Shot Location** |  |  | **Success** | **Fail** | **χ^2^(df=1)** | ***p*** | ***φ*** |
| --- | --- | --- | --- | --- | --- | --- | --- |
| **PL** | Win | Frequency | 142 | 107 | 0.046 | 0.083 | 0.010 |
|  |  | Percentage | 57.0% | 43.0% |  |  |  |
|  | Lose | Frequency | 134 | 105 |  |  |  |
|  |  | Percentage | 56.1% | 43.9% |  |  |  |
| **PH** | Win | Frequency | 37 | 37 | 8.902* | 0.003 | 0.241 |
|  |  | Percentage | 50.0% | 50.0% |  |  |  |
|  | Lose | Frequency | 21 | 58 |  |  |  |
|  |  | Percentage | 26.6% | 73.4% |  |  |  |
| **Top** | Win | Frequency | 35 | 56 | 0.162 | 0.687 | -0.031 |
|  |  | Percentage | 38.5% | 61.5% |  |  |  |
|  | Lose | Frequency | 34 | 48 |  |  |  |
|  |  | Percentage | 41.5% | 58.5% |  |  |  |
| **Corner** | Win | Frequency | 90 | 112 | 0.026 | 0.872 | 0.008 |
|  |  | Percentage | 44.6% | 55.4% |  |  |  |
|  | Lose | Frequency | 84 | 108 |  |  |  |
|  |  | Percentage | 43.8% | 56.3% |  |  |  |
| **Wing** | Win | Frequency | 70 | 126 | 1.588 | 0.208 | 0.068 |
|  |  | Percentage | 35.7% | 64.3% |  |  |  |
|  | Lose | Frequency | 43 | 104 |  |  |  |
|  |  | Percentage | 29.3% | 70.7% |  |  |  |
| **3P** | Win | Frequency | 39 | 85 | 4.275* | 0.039 | 0.128 |
|  |  | Percentage | 31.5% | 68.5% |  |  |  |
|  | Lose | Frequency | 28 | 110 |  |  |  |
|  |  | Percentage | 20.3% | 79.7% |  |  |  |

PL, paint-low; PH, paint-high; 3P, 3-point field goal area.

**p* < 0.05.

TABLE S23 Differences in shot-success rate in screen location.

| **Screen Location** |  |  | **Success** | **Fail** | **χ^2^(df=1)** | ***p*** | ***φ*** |
| --- | --- | --- | --- | --- | --- | --- | --- |
| **PL** | Win | Frequency | 35 | 18 | 1.981 | 0.159 | 0.135 |
|  |  | Percentage | 66.0% | 34.0% |  |  |  |
|  | Lose | Frequency | 29 | 26 |  |  |  |
|  |  | Percentage | 52.7% | 47.3% |  |  |  |
| **PH** | Win | Frequency | 38 | 51 | 0.025 | 0.874 | 0.013 |
|  |  | Percentage | 42.7% | 57.3% |  |  |  |
|  | Lose | Frequency | 24 | 34 |  |  |  |
|  |  | Percentage | 41.4% | 58.6% |  |  |  |
| **Top** | Win | Frequency | 28 | 42 | 0.994 | 0.319 | 0.074 |
|  |  | Percentage | 40.0% | 60.0% |  |  |  |
|  | Lose | Frequency | 37 | 76 |  |  |  |
|  |  | Percentage | 32.7% | 67.3% |  |  |  |
| **Corner** | Win | Frequency | 125 | 135 | 1.500 | 0.221 | 0.055 |
|  |  | Percentage | 48.1% | 51.9% |  |  |  |
|  | Lose | Frequency | 97 | 131 |  |  |  |
|  |  | Percentage | 42.5% | 57.5% |  |  |  |
| **Wing** | Win | Frequency | 155 | 240 | 1.088 | 0.297 | 0.038 |
|  |  | Percentage | 39.2% | 60.8% |  |  |  |
|  | Lose | Frequency | 124 | 225 |  |  |  |
|  |  | Percentage | 35.5% | 64.5% |  |  |  |
| **3P** | Win | Frequency | 27 | 25 | 0.474 | 0.491 | 0.063 |
|  |  | Percentage | 51.9% | 48.1% |  |  |  |
|  | Lose | Frequency | 31 | 37 |  |  |  |
|  |  | Percentage | 45.6% | 54.4% |  |  |  |

PL, paint-low; PH, paint-high; 3P, 3-point field goal area.

TABLE S24 Differences in shot-success rate in pass location.

| **Pass Location** |  |  | **Success** | **Fail** | **χ^2^(df=1)** | ***p*** | ***φ*** |
| --- | --- | --- | --- | --- | --- | --- | --- |
| **PL** | Win | Frequency | 11 | 11 | 0.000 | 1.000 | 0.010 |
|  |  | Percentage | 50.0% | 50.0% |  |  |  |
|  | Lose | Frequency | 6 | 6 |  |  |  |
|  |  | Percentage | 50.0% | 50.0% |  |  |  |
| **PH** | Win | Frequency | 41 | 44 | 1.535 | 0.215 | 0.100 |
|  |  | Percentage | 48.2% | 51.8% |  |  |  |
|  | Lose | Frequency | 26 | 42 |  |  |  |
|  |  | Percentage | 38.2% | 61.8% |  |  |  |
| **Top** | Win | Frequency | 39 | 56 | 2.378 | 0.123 | -0.107 |
|  |  | Percentage | 41.1% | 58.9% |  |  |  |
|  | Lose | Frequency | 58 | 54 |  |  |  |
|  |  | Percentage | 51.8% | 48.2% |  |  |  |
| **Corner** | Win | Frequency | 55 | 75 | 0.056 | 0.813 | -0.016 |
|  |  | Percentage | 42.3% | 57.7% |  |  |  |
|  | Lose | Frequency | 43 | 55 |  |  |  |
|  |  | Percentage | 43.9% | 56.1% |  |  |  |
| **Wing** | Win | Frequency | 69 | 90 | 0.082 | 0.774 | -0.016 |
|  |  | Percentage | 43.4% | 56.6% |  |  |  |
|  | Lose | Frequency | 76 | 93 |  |  |  |
|  |  | Percentage | 45.0% | 55.0% |  |  |  |
| **3P** | Win | Frequency | 47 | 44 | 7.080* | 0.008 | 0.184 |
|  |  | Percentage | 51.6% | 48.4% |  |  |  |
|  | Lose | Frequency | 39 | 78 |  |  |  |
|  |  | Percentage | 33.3% | 66.7% |  |  |  |

PL, paint-low; PH, paint-high; 3P, 3-point field goal area.

**p* < 0.05.

TABLE S25 Differences in shot-success rate in type of screen.

| **Type of Screen** |  |  | **Success** | **Fail** | **χ^2^(df=1)** | ***p*** | ***φ*** |
| --- | --- | --- | --- | --- | --- | --- | --- |
| **On-the-ball screen** | Win | Frequency | 292 | 400 | 1.788 | 0.181 | 0.037 |
|  |  | Percentage | 42.2% | 57.8% |  |  |  |
|  | Lose | Frequency | 249 | 396 |  |  |  |
|  |  | Percentage | 38.6% | 61.4% |  |  |  |
| **Off-the-ball screen** | Win | Frequency | 121 | 123 | 3.583 | 0.058 | 0.087 |
|  |  | Percentage | 49.6% | 50.4% |  |  |  |
|  | Lose | Frequency | 95 | 137 |  |  |  |
|  |  | Percentage | 40.9% | 59.1% |  |  |  |

TABLE S26 Differences in shot-success rate in type of screen-play.

| **Type of Screen-play** |  |  | **Success** | **Fail** | **χ^2^(df=1)** | ***p*** | ***φ*** |
| --- | --- | --- | --- | --- | --- | --- | --- |
| **ON-U** | Win | Frequency | 154 | 206 | 7.209* | 0.007 | 0.104 |
|  |  | Percentage | 42.8% | 57.2% |  |  |  |
|  | Lose | Frequency | 102 | 210 |  |  |  |
|  |  | Percentage | 32.7% | 67.3% |  |  |  |
| **ON-S** | Win | Frequency | 31 | 38 | 2.075 | 0.150 | -0.122 |
|  |  | Percentage | 44.9% | 55.1% |  |  |  |
|  | Lose | Frequency | 40 | 30 |  |  |  |
|  |  | Percentage | 57.1% | 42.9% |  |  |  |
| **ON-A** | Win | Frequency | 74 | 104 | 0.726 | 0.394 | 0.045 |
|  |  | Percentage | 41.6% | 58.4% |  |  |  |
|  | Lose | Frequency | 65 | 110 |  |  |  |
|  |  | Percentage | 37.1% | 62.9% |  |  |  |
| **ON-E** | Win | Frequency | 33 | 52 | 1.396 | 0.237 | -0.090 |
|  |  | Percentage | 38.8% | 61.2% |  |  |  |
|  | Lose | Frequency | 42 | 46 |  |  |  |
|  |  | Percentage | 47.7% | 52.3% |  |  |  |
| **OF-U** | Win | Frequency | 108 | 118 | 2.312 | 0.128 | 0.073 |
|  |  | Percentage | 47.8% | 52.2% |  |  |  |
|  | Lose | Frequency | 86 | 126 |  |  |  |
|  |  | Percentage | 40.6% | 59.4% |  |  |  |
| **OF-S** | Win | Frequency | 13 | 5 | 2.880 | 0.090 | 0.275 |
|  |  | Percentage | 72.2% | 27.8% |  |  |  |
|  | Lose | Frequency | 9 | 11 |  |  |  |
|  |  | Percentage | 45.0% | 55.0% |  |  |  |

ON-U, the plays where the user shot using the on-the-ball screen; ON-S, plays where the screener of the on-the-ball screen shot after receiving a pass from the user; ON-A, plays where another player shot after receiving a pass from the user of the on-the-ball screen; ON-E, plays that led to a shot through two or more extra passes after the user used the on-the-ball screen; OF-U, plays where the user shot using the off-the-ball screen; OF-S, plays where the screener of the off-the-ball screen shot.

**p* < 0.05.

TABLE S27 Differences in shot-success rate in movement of on-the-ball screen plays.

| **Movement of on-the-ball screen plays** |  |  | **Success** | **Fail** | **χ^2^(df=1)** | ***p*** | ***φ*** |
| --- | --- | --- | --- | --- | --- | --- | --- |
| **Around** | Win | Frequency | 8 | 11 | 0.033 | 0.855 | -0.029 |
|  |  | Percentage | 42.1% | 57.9% |  |  |  |
|  | Lose | Frequency | 9 | 11 |  |  |  |
|  |  | Percentage | 45.0% | 55.0% |  |  |  |
| **Center-line** | Win | Frequency | 130 | 169 | 2.672 | 0.102 | 0.068 |
|  |  | Percentage | 43.5% | 56.5% |  |  |  |
|  | Lose | Frequency | 105 | 180 |  |  |  |
|  |  | Percentage | 36.8% | 63.2% |  |  |  |
| **End-line** | Win | Frequency | 148 | 199 | 0.656 | 0.418 | 0.031 |
|  |  | Percentage | 42.7% | 57.3% |  |  |  |
|  | Lose | Frequency | 127 | 194 |  |  |  |
|  |  | Percentage | 39.6% | 60.4% |  |  |  |
| **ON-Down** | Win | Frequency | 5 | 21 | 2.796 | 0.094 | -0.249 |
|  |  | Percentage | 19.2% | 80.8% |  |  |  |
|  | Lose | Frequency | 8 | 11 |  |  |  |
|  |  | Percentage | 42.1% | 57.9% |  |  |  |

Around, plays where the screener held a ball; Center-line, plays where the user moved toward the center-line side against the screener; End-line, plays where the user moved toward the end-line side against the screener; ON-Down, plays where the screener was on the center-line side of the defense who protected the user holding a ball in a Top or 3P on Top extension.

TABLE S28 Differences in shot-success rate in movement of off-the-ball screen plays.

| **Movement of off-the-ball screen plays** |  |  | **Success** | **Fail** | **χ^2^(df=1)** | ***p*** | ***φ*** |
| --- | --- | --- | --- | --- | --- | --- | --- |
| **Back** | Win | Frequency | 29 | 23 | 3.801 | 0.051 | 0.185 |
|  |  | Percentage | 55.8% | 44.2% |  |  |  |
|  | Lose | Frequency | 22 | 37 |  |  |  |
|  |  | Percentage | 37.3% | 62.7% |  |  |  |
| **Cross** | Win | Frequency | 17 | 8 | 0.266 | 0.606 | 0.074 |
|  |  | Percentage | 68.0% | 32.0% |  |  |  |
|  | Lose | Frequency | 14 | 9 |  |  |  |
|  |  | Percentage | 60.9% | 39.1% |  |  |  |
| **Down** | Win | Frequency | 27 | 27 | 5.302* | 0.021 | 0.220 |
|  |  | Percentage | 50.0% | 50.0% |  |  |  |
|  | Lose | Frequency | 16 | 40 |  |  |  |
|  |  | Percentage | 28.6% | 71.4% |  |  |  |
| **Flare** | Win | Frequency | 47 | 65 | 0.297 | 0.586 | -0.038 |
|  |  | Percentage | 42.0% | 58.0% |  |  |  |
|  | Lose | Frequency | 43 | 51 |  |  |  |
|  |  | Percentage | 45.7% | 54.3% |  |  |  |

Back, plays where the screener was on the end-line side of the defense who protected the user; Cross, plays where the screener was on the middle-line (the imaginary line connecting baskets running through the center of the court) side of the defense who protected the user; Down, plays where the screener on the center-line side of the defense who protected the user; Flare, plays where the screener on the side-line side of the defense who protected the user.

**p* < 0.05.

TABLE S29 Differences in shot-success rate depending on the PC of the shooter.

| **Shooter** |  |  | **Success** | **Fail** | **χ^2^(df=1)** | ***p*** | ***φ*** |
| --- | --- | --- | --- | --- | --- | --- | --- |
| **Low** | Win | Frequency | 24 | 36 | 0.906 | 0.341 | -0.081 |
|  |  | Percentage | 40.0% | 60.0% |  |  |  |
|  | Lose | Frequency | 38 | 41 |  |  |  |
|  |  | Percentage | 48.1% | 51.9% |  |  |  |
| **Middle** | Win | Frequency | 184 | 256 | 0.446 | 0.504 | 0.023 |
|  |  | Percentage | 41.8% | 58.2% |  |  |  |
|  | Lose | Frequency | 157 | 240 |  |  |  |
|  |  | Percentage | 39.5% | 60.5% |  |  |  |
| **High** | Win | Frequency | 205 | 231 | 8.323* | 0.004 | 0.100 |
|  |  | Percentage | 47.0% | 53.0% |  |  |  |
|  | Lose | Frequency | 149 | 252 |  |  |  |
|  |  | Percentage | 37.2% | 62.8% |  |  |  |

PC, player classification; Low, low-point classification; Middle, middle-point classification; High, high-point classification.

**p* < 0.05.

TABLE S30 Differences in shot-success rate of the shooter in shot location.

| **Shooter** | **Shot Location** |  |  | **Success** | **Fail** | **χ^2^(df=1)** | ***p*** | ***φ*** |
| --- | --- | --- | --- | --- | --- | --- | --- | --- |
| **Low** | **PL** | Win | Frequency | 10 | 11 | 0.707 | 0.400 | -0.116 |
|  |  |  | Percentage | 47.6% | 52.4% |  |  |  |
|  |  | Lose | Frequency | 19 | 13 |  |  |  |
|  |  |  | Percentage | 59.4% | 40.6% |  |  |  |
| **Middle** | **PL** | Win | Frequency | 65 | 59 | 0.002 | 0.968 | -0.003 |
|  |  |  | Percentage | 52.4% | 47.6% |  |  |  |
|  |  | Lose | Frequency | 69 | 62 |  |  |  |
|  |  |  | Percentage | 52.7% | 47.3% |  |  |  |
| **High** | **PL** | Win | Frequency | 67 | 37 | 0.285 | 0.593 | 0.040 |
|  |  |  | Percentage | 64.4% | 35.6% |  |  |  |
|  |  | Lose | Frequency | 46 | 30 |  |  |  |
|  |  |  | Percentage | 60.5% | 39.5% |  |  |  |
| **Low** | **PH** | Win | Frequency | 0 | 1 |  |  |  |
|  |  |  | Percentage | 0.0% | 100.0% |  |  |  |
|  |  | Lose | Frequency | 0 | 2 |  |  |  |
|  |  |  | Percentage | 0.0% | 100.0% |  |  |  |
| **Middle** | **PH** | Win | Frequency | 13 | 15 | 1.054 | 0.305 | 0.124 |
|  |  |  | Percentage | 46.4% | 53.6% |  |  |  |
|  |  | Lose | Frequency | 14 | 27 |  |  |  |
|  |  |  | Percentage | 34.1% | 65.9% |  |  |  |
| **High** | **PH** | Win | Frequency | 24 | 21 | 9.723* | 0.002 | 0.346 |
|  |  |  | Percentage | 53.3% | 46.7% |  |  |  |
|  |  | Lose | Frequency | 7 | 29 |  |  |  |
|  |  |  | Percentage | 19.4% | 80.6% |  |  |  |
| **Low** | **Top** | Win | Frequency | 8 | 10 | 0.000 | 1.000 | 0.000 |
|  |  |  | Percentage | 44.4% | 55.6% |  |  |  |
|  |  | Lose | Frequency | 12 | 15 |  |  |  |
|  |  |  | Percentage | 44.4% | 55.6% |  |  |  |
| **Middle** | **Top** | Win | Frequency | 19 | 28 | 0.009 | 0.926 | 0.010 |
|  |  |  | Percentage | 40.4% | 59.6% |  |  |  |
|  |  | Lose | Frequency | 13 | 20 |  |  |  |
|  |  |  | Percentage | 39.4% | 60.6% |  |  |  |
| **High** | **Top** | Win | Frequency | 8 | 18 | 0.536 | 0.464 | -0.106 |
|  |  |  | Percentage | 30.8% | 69.2% |  |  |  |
|  |  | Lose | Frequency | 9 | 13 |  |  |  |
|  |  |  | Percentage | 40.9% | 59.1% |  |  |  |
| **Low** | **Corner** | Win | Frequency | 6 | 9 | 0.056 | 0.812 | 0.044 |
|  |  |  | Percentage | 40.0% | 60.0% |  |  |  |
|  |  | Lose | Frequency | 5 | 9 |  |  |  |
|  |  |  | Percentage | 35.7% | 64.3% |  |  |  |
| **Middle** | **Corner** | Win | Frequency | 37 | 56 | 0.304 | 0.581 | -0.042 |
|  |  |  | Percentage | 39.8% | 60.2% |  |  |  |
|  |  | Lose | Frequency | 36 | 46 |  |  |  |
|  |  |  | Percentage | 43.9% | 56.1% |  |  |  |
| **High** | **Corner** | Win | Frequency | 47 | 47 | 0.517 | 0.472 | 0.052 |
|  |  |  | Percentage | 50.0% | 50.0% |  |  |  |
|  |  | Lose | Frequency | 43 | 53 |  |  |  |
|  |  |  | Percentage | 44.8% | 55.2% |  |  |  |
| **Low** | **Wing** | Win | Frequency | 0 | 4 |  | 0.429 (**) | -0.577 |
|  |  |  | Percentage | 0.0% | 100.0% |  |  |  |
|  |  | Lose | Frequency | 2 | 2 |  |  |  |
|  |  |  | Percentage | 50.0% | 50.0% |  |  |  |
| **Middle** | **Wing** | Win | Frequency | 34 | 59 | 2.542 | 0.111 | 0.130 |
|  |  |  | Percentage | 36.6% | 63.4% |  |  |  |
|  |  | Lose | Frequency | 14 | 44 |  |  |  |
|  |  |  | Percentage | 24.1% | 75.9% |  |  |  |
| **High** | **Wing** | Win | Frequency | 36 | 63 | 0.430 | 0.512 | 0.048 |
|  |  |  | Percentage | 36.4% | 63.6% |  |  |  |
|  |  | Lose | Frequency | 27 | 58 |  |  |  |
|  |  |  | Percentage | 31.8% | 68.2% |  |  |  |
| **Low** | **3P** | Win | Frequency | 0 | 1 |  |  |  |
|  |  |  | Percentage | 0.0% | 100.0% |  |  |  |
|  |  | Lose | Frequency | 0 | 1 |  |  |  |
|  |  |  | Percentage | 0.0% | 100.0% |  |  |  |
| **Middle** | **3P** | Win | Frequency | 16 | 39 | 0.893 | 0.345 | 0.091 |
|  |  |  | Percentage | 29.1% | 70.9% |  |  |  |
|  |  | Lose | Frequency | 11 | 41 |  |  |  |
|  |  |  | Percentage | 21.2% | 78.8% |  |  |  |
| **High** | **3P** | Win | Frequency | 23 | 45 | 3.902* | 0.048 | 0.159 |
|  |  |  | Percentage | 33.8% | 66.2% |  |  |  |
|  |  | Lose | Frequency | 17 | 69 |  |  |  |
|  |  |  | Percentage | 19.8% | 80.2% |  |  |  |

Low, low-point classification; Middle, middle-point classification; High, high-point classification; PL, paint-low; PH, paint-high; 3P, 3-point field goal area.

**p* < 0.05.

**We adopted *p*-value by Fisher's method.

TABLE S31 Differences in shot-success rate of the shooter in screen location.

| **Shooter** | **Screen Location** |  |  | **Success** | **Fail** | **χ^2^(df=1)** | ***p*** | ***φ*** |
| --- | --- | --- | --- | --- | --- | --- | --- | --- |
| **Low** | **PL** | Win | Frequency | 0 | 1 |  | 1.000 (**) | -0.447 |
|  |  |  | Percentage | 0.0% | 100.0% |  |  |  |
|  |  | Lose | Frequency | 3 | 2 |  |  |  |
|  |  |  | Percentage | 60.0% | 40.0% |  |  |  |
| **Middle** | **PL** | Win | Frequency | 15 | 9 | 0.512 | 0.474 | 0.104 |
|  |  |  | Percentage | 62.5% | 37.5% |  |  |  |
|  |  | Lose | Frequency | 12 | 11 |  |  |  |
|  |  |  | Percentage | 52.2% | 47.8% |  |  |  |
| **High** | **PL** | Win | Frequency | 20 | 8 | 2.232 | 0.135 | 0.201 |
|  |  |  | Percentage | 71.4% | 28.6% |  |  |  |
|  |  | Lose | Frequency | 14 | 13 |  |  |  |
|  |  |  | Percentage | 51.9% | 48.1% |  |  |  |
| **Low** | **PH** | Win | Frequency | 1 | 1 |  | 1.000 (**) | 0.250 |
|  |  |  | Percentage | 50.0% | 50.0% |  |  |  |
|  |  | Lose | Frequency | 3 | 1 |  |  |  |
|  |  |  | Percentage | 75.0% | 25.0% |  |  |  |
| **Middle** | **PH** | Win | Frequency | 18 | 20 | 0.110 | 0.740 | 0.040 |
|  |  |  | Percentage | 47.4% | 52.6% |  |  |  |
|  |  | Lose | Frequency | 13 | 17 |  |  |  |
|  |  |  | Percentage | 43.3% | 56.7% |  |  |  |
| **High** | **PH** | Win | Frequency | 17 | 30 | 0.038 | 0.846 | -0.023 |
|  |  |  | Percentage | 36.2% | 63.8% |  |  |  |
|  |  | Lose | Frequency | 10 | 16 |  |  |  |
|  |  |  | Percentage | 38.5% | 61.5% |  |  |  |
| **Low** | **Top** | Win | Frequency | 0 | 2 |  |  |  |
|  |  |  | Percentage | 0.0% | 100.0% |  |  |  |
|  |  | Lose | Frequency | 0 | 6 |  |  |  |
|  |  |  | Percentage | 0.0% | 100.0% |  |  |  |
| **Middle** | **Top** | Win | Frequency | 14 | 20 | 0.513 | 0.474 | 0.081 |
|  |  |  | Percentage | 41.2% | 58.8% |  |  |  |
|  |  | Lose | Frequency | 15 | 30 |  |  |  |
|  |  |  | Percentage | 33.3% | 66.7% |  |  |  |
| **High** | **Top** | Win | Frequency | 14 | 20 | 0.304 | 0.582 | 0.056 |
|  |  |  | Percentage | 41.2% | 58.8% |  |  |  |
|  |  | Lose | Frequency | 22 | 40 |  |  |  |
|  |  |  | Percentage | 35.5% | 64.5% |  |  |  |
| **Low** | **Corner** | Win | Frequency | 15 | 15 | 0.025 | 0.875 | 0.022 |
|  |  |  | Percentage | 50.0% | 50.0% |  |  |  |
|  |  | Lose | Frequency | 11 | 12 |  |  |  |
|  |  |  | Percentage | 47.8% | 52.2% |  |  |  |
| **Middle** | **Corner** | Win | Frequency | 49 | 66 | 0.158 | 0.691 | 0.026 |
|  |  |  | Percentage | 42.6% | 57.4% |  |  |  |
|  |  | Lose | Frequency | 44 | 66 |  |  |  |
|  |  |  | Percentage | 40.0% | 60.0% |  |  |  |
| **High** | **Corner** | Win | Frequency | 61 | 54 | 1.624 | 0.203 | 0.088 |
|  |  |  | Percentage | 53.0% | 47.0% |  |  |  |
|  |  | Lose | Frequency | 42 | 53 |  |  |  |
|  |  |  | Percentage | 44.2% | 55.8% |  |  |  |
| **Low** | **Wing** | Win | Frequency | 4 | 15 | 5.602* | 0.018 | -0.311 |
|  |  |  | Percentage | 21.1% | 78.9% |  |  |  |
|  |  | Lose | Frequency | 21 | 18 |  |  |  |
|  |  |  | Percentage | 53.8% | 46.2% |  |  |  |
| **Middle** | **Wing** | Win | Frequency | 76 | 122 | 0.151 | 0.698 | 0.021 |
|  |  |  | Percentage | 38.4% | 61.6% |  |  |  |
|  |  | Lose | Frequency | 56 | 98 |  |  |  |
|  |  |  | Percentage | 36.4% | 63.6% |  |  |  |
| **High** | **Wing** | Win | Frequency | 75 | 103 | 5.169* | 0.023 | 0.124 |
|  |  |  | Percentage | 42.1% | 57.9% |  |  |  |
|  |  | Lose | Frequency | 47 | 109 |  |  |  |
|  |  |  | Percentage | 30.1% | 69.9% |  |  |  |
| **Low** | **3P** | Win | Frequency | 1 | 1 |  | 1.000 | 0.000 |
|  |  |  | Percentage | 50.0% | 50.0% |  |  |  |
|  |  | Lose | Frequency | 2 | 2 |  |  |  |
|  |  |  | Percentage | 50.0% | 50.0% |  |  |  |
| **Middle** | **3P** | Win | Frequency | 11 | 15 | 0.224 | 0.636 | -0.062 |
|  |  |  | Percentage | 42.3% | 57.7% |  |  |  |
|  |  | Lose | Frequency | 16 | 17 |  |  |  |
|  |  |  | Percentage | 48.5% | 51.5% |  |  |  |
| **High** | **3P** | Win | Frequency | 15 | 9 | 2.289 | 0.130 | 0.204 |
|  |  |  | Percentage | 62.5% | 37.5% |  |  |  |
|  |  | Lose | Frequency | 13 | 18 |  |  |  |
|  |  |  | Percentage | 41.9% | 58.1% |  |  |  |

Low, low-point classification; Middle, middle-point classification; High, high-point classification; PL, paint-low; PH, paint-high; 3P, 3-point field goal area.

**p* < 0.05.

**We adopted *p*-value by Fisher's method.

TABLE S32 Differences in shot-success rate of the shooter in pass location.

| **Shooter** | **Pass Location** |  |  | **Success** | **Fail** | **χ^2^(df=1)** | ***p*** | ***φ*** |
| --- | --- | --- | --- | --- | --- | --- | --- | --- |
| **Low** | **PL** | Win | Frequency | 0 | 1 |  |  |  |
|  |  |  | Percentage | 0.0% | 100.0% |  |  |  |
|  |  | Lose | Frequency | 0 | 1 |  |  |  |
|  |  |  | Percentage | 0.0% | 100.0% |  |  |  |
| **Middle** | **PL** | Win | Frequency | 6 | 8 |  | 0.628 (**) | -0.218 |
|  |  |  | Percentage | 42.9% | 57.1% |  |  |  |
|  |  | Lose | Frequency | 4 | 2 |  |  |  |
|  |  |  | Percentage | 66.7% | 33.3% |  |  |  |
| **High** | **PL** | Win | Frequency | 5 | 3 |  | 0.592 (**) | 0.220 |
|  |  |  | Percentage | 62.5% | 37.5% |  |  |  |
|  |  | Lose | Frequency | 2 | 3 |  |  |  |
|  |  |  | Percentage | 40.0% | 60.0% |  |  |  |
| **Low** | **PH** | Win | Frequency | 4 | 6 |  | 0.335 (**) | -0.310 |
|  |  |  | Percentage | 40.0% | 60.0% |  |  |  |
|  |  | Lose | Frequency | 5 | 2 |  |  |  |
|  |  |  | Percentage | 71.4% | 28.6% |  |  |  |
| **Middle** | **PH** | Win | Frequency | 18 | 24 | 0.449 | 0.503 | 0.073 |
|  |  |  | Percentage | 42.9% | 57.1% |  |  |  |
|  |  | Lose | Frequency | 15 | 27 |  |  |  |
|  |  |  | Percentage | 35.7% | 64.3% |  |  |  |
| **High** | **PH** | Win | Frequency | 19 | 14 | 3.264 | 0.071 | 0.251 |
|  |  |  | Percentage | 57.6% | 42.4% |  |  |  |
|  |  | Lose | Frequency | 6 | 13 |  |  |  |
|  |  |  | Percentage | 31.6% | 68.4% |  |  |  |
| **Low** | **Top** | Win | Frequency | 6 | 6 | 0.540 | 0.462 | -0.144 |
|  |  |  | Percentage | 50.0% | 50.0% |  |  |  |
|  |  | Lose | Frequency | 9 | 5 |  |  |  |
|  |  |  | Percentage | 64.3% | 35.7% |  |  |  |
| **Middle** | **Top** | Win | Frequency | 16 | 29 | 5.129* | 0.024 | -0.229 |
|  |  |  | Percentage | 35.6% | 64.4% |  |  |  |
|  |  | Lose | Frequency | 31 | 22 |  |  |  |
|  |  |  | Percentage | 58.5% | 41.5% |  |  |  |
| **High** | **Top** | Win | Frequency | 17 | 21 | 0.190 | 0.663 | 0.048 |
|  |  |  | Percentage | 44.7% | 55.3% |  |  |  |
|  |  | Lose | Frequency | 18 | 27 |  |  |  |
|  |  |  | Percentage | 40.0% | 60.0% |  |  |  |
| **Low** | **Corner** | Win | Frequency | 9 | 6 | 0.157 | 0.692 | -0.069 |
|  |  |  | Percentage | 60.0% | 40.0% |  |  |  |
|  |  | Lose | Frequency | 12 | 6 |  |  |  |
|  |  |  | Percentage | 66.7% | 33.3% |  |  |  |
| **Middle** | **Corner** | Win | Frequency | 23 | 43 | 0.187 | 0.665 | -0.040 |
|  |  |  | Percentage | 34.8% | 65.2% |  |  |  |
|  |  | Lose | Frequency | 19 | 30 |  |  |  |
|  |  |  | Percentage | 38.8% | 61.2% |  |  |  |
| **High** | **Corner** | Win | Frequency | 23 | 26 | 0.522 | 0.470 | 0.081 |
|  |  |  | Percentage | 46.9% | 53.1% |  |  |  |
|  |  | Lose | Frequency | 12 | 19 |  |  |  |
|  |  |  | Percentage | 38.7% | 61.3% |  |  |  |
| **Low** | **Wing** | Win | Frequency | 4 | 13 | 1.710 | 0.191 | -0.207 |
|  |  |  | Percentage | 23.5% | 76.5% |  |  |  |
|  |  | Lose | Frequency | 10 | 13 |  |  |  |
|  |  |  | Percentage | 43.5% | 56.5% |  |  |  |
| **Middle** | **Wing** | Win | Frequency | 39 | 34 | 1.431 | 0.232 | 0.097 |
|  |  |  | Percentage | 53.4% | 46.6% |  |  |  |
|  |  | Lose | Frequency | 35 | 45 |  |  |  |
|  |  |  | Percentage | 43.8% | 56.3% |  |  |  |
| **High** | **Wing** | Win | Frequency | 26 | 43 | 1.193 | 0.275 | -0.094 |
|  |  |  | Percentage | 37.7% | 62.3% |  |  |  |
|  |  | Lose | Frequency | 31 | 35 |  |  |  |
|  |  |  | Percentage | 47.0% | 53.0% |  |  |  |
| **Low** | **3P** | Win | Frequency | 1 | 1 |  | 0.295 (**) | 0.409 |
|  |  |  | Percentage | 50.0% | 50.0% |  |  |  |
|  |  | Lose | Frequency | 1 | 10 |  |  |  |
|  |  |  | Percentage | 9.1% | 90.9% |  |  |  |
| **Middle** | **3P** | Win | Frequency | 20 | 20 | 1.810 | 0.179 | 0.146 |
|  |  |  | Percentage | 50.0% | 50.0% |  |  |  |
|  |  | Lose | Frequency | 16 | 29 |  |  |  |
|  |  |  | Percentage | 35.6% | 64.4% |  |  |  |
| **High** | **3P** | Win | Frequency | 26 | 23 | 3.191 | 0.074 | 0.170 |
|  |  |  | Percentage | 53.1% | 46.9% |  |  |  |
|  |  | Lose | Frequency | 22 | 39 |  |  |  |
|  |  |  | Percentage | 36.1% | 63.9% |  |  |  |

Low, low-point classification; Middle, middle-point classification; High, high-point classification; PL, paint-low; PH, paint-high; 3P, 3-point field goal area.

**p* < 0.05.

**We adopted *p*-value by Fisher's method.

TABLE S33 Differences in shot-success rate of the shooter in type of screen.

| **Shooter** | **Type of Screen** |  |  | **Success** | **Fail** | **χ^2^(df=1)** | ***p*** | ***φ*** |
| --- | --- | --- | --- | --- | --- | --- | --- | --- |
| **Low** | **On-the-ball screen** | Win | Frequency | 19 | 29 | 0.736 | 0.391 | -0.081 |
|  |  |  | Percentage | 39.6% | 60.4% |  |  |  |
|  |  | Lose | Frequency | 31 | 34 |  |  |  |
|  |  |  | Percentage | 47.7% | 52.3% |  |  |  |
| **Middle** | **On-the-ball screen** | Win | Frequency | 133 | 196 | 0.080 | 0.777 | 0.011 |
|  |  |  | Percentage | 40.4% | 59.6% |  |  |  |
|  |  | Lose | Frequency | 114 | 176 |  |  |  |
|  |  |  | Percentage | 39.3% | 60.7% |  |  |  |
| **High** | **On-the-ball screen** | Win | Frequency | 140 | 175 | 4.621* | 0.032 | 0.087 |
|  |  |  | Percentage | 44.4% | 55.6% |  |  |  |
|  |  | Lose | Frequency | 104 | 186 |  |  |  |
|  |  |  | Percentage | 35.9% | 64.1% |  |  |  |
| **Low** | **Off-the-ball screen** | Win | Frequency | 5 | 7 | 0.181 | 0.671 | -0.083 |
|  |  |  | Percentage | 41.7% | 58.3% |  |  |  |
|  |  | Lose | Frequency | 7 | 7 |  |  |  |
|  |  |  | Percentage | 50.0% | 50.0% |  |  |  |
| **Middle** | **Off-the-ball screen** | Win | Frequency | 51 | 60 | 0.737 | 0.391 | 0.058 |
|  |  |  | Percentage | 45.9% | 54.1% |  |  |  |
|  |  | Lose | Frequency | 43 | 64 |  |  |  |
|  |  |  | Percentage | 40.2% | 59.8% |  |  |  |
| **High** | **Off-the-ball screen** | Win | Frequency | 65 | 56 | 4.032* | 0.045 | 0.132 |
|  |  |  | Percentage | 53.7% | 46.3% |  |  |  |
|  |  | Lose | Frequency | 45 | 66 |  |  |  |
|  |  |  | Percentage | 40.5% | 59.5% |  |  |  |

Low, low-point classification; Middle, middle-point classification; High, high-point classification.

**p* < 0.05.

TABLE S34 Differences in shot-success rate of the shooter in type of screen-play.

| **Shooter** | **Type of Screen-play** |  |  | **Success** | **Fail** | **χ^2^(df=1)** | ***p*** | ***φ*** |
| --- | --- | --- | --- | --- | --- | --- | --- | --- |
| **Low** | **ON-U** | Win | Frequency | 0 | 4 |  | 1.000 (**) | -0.272 |
|  |  |  | Percentage | 0.0% | 100.0% |  |  |  |
|  |  | Lose | Frequency | 1 | 5 |  |  |  |
|  |  |  | Percentage | 16.7% | 83.3% |  |  |  |
| **Middle** | **ON-U** | Win | Frequency | 63 | 99 | 1.468 | 0.226 | 0.071 |
|  |  |  | Percentage | 38.9% | 61.1% |  |  |  |
|  |  | Lose | Frequency | 42 | 89 |  |  |  |
|  |  |  | Percentage | 32.1% | 67.9% |  |  |  |
| **High** | **ON-U** | Win | Frequency | 91 | 103 | 6.638* | 0.010 | 0.134 |
|  |  |  | Percentage | 46.9% | 53.1% |  |  |  |
|  |  | Lose | Frequency | 59 | 116 |  |  |  |
|  |  |  | Percentage | 33.7% | 66.3% |  |  |  |
| **Low** | **ON-S** | Win | Frequency | 7 | 7 | 1.146 | 0.284 | -0.186 |
|  |  |  | Percentage | 50.0% | 50.0% |  |  |  |
|  |  | Lose | Frequency | 13 | 6 |  |  |  |
|  |  |  | Percentage | 68.4% | 31.6% |  |  |  |
| **Middle** | **ON-S** | Win | Frequency | 12 | 18 | 2.200 | 0.138 | -0.183 |
|  |  |  | Percentage | 40.0% | 60.0% |  |  |  |
|  |  | Lose | Frequency | 21 | 15 |  |  |  |
|  |  |  | Percentage | 58.3% | 41.7% |  |  |  |
| **High** | **ON-S** | Win | Frequency | 12 | 13 | 0.242 | 0.622 | 0.078 |
|  |  |  | Percentage | 48.0% | 52.0% |  |  |  |
|  |  | Lose | Frequency | 6 | 9 |  |  |  |
|  |  |  | Percentage | 40.0% | 60.0% |  |  |  |
| **Low** | **ON-A** | Win | Frequency | 10 | 12 | 0.023 | 0.879 | 0.021 |
|  |  |  | Percentage | 45.5% | 54.5% |  |  |  |
|  |  | Lose | Frequency | 13 | 17 |  |  |  |
|  |  |  | Percentage | 43.3% | 56.7% |  |  |  |
| **Middle** | **ON-A** | Win | Frequency | 44 | 60 | 1.108 | 0.292 | 0.078 |
|  |  |  | Percentage | 42.3% | 57.7% |  |  |  |
|  |  | Lose | Frequency | 27 | 51 |  |  |  |
|  |  |  | Percentage | 34.6% | 65.4% |  |  |  |
| **High** | **ON-A** | Win | Frequency | 20 | 32 | 0.016 | 0.898 | 0.012 |
|  |  |  | Percentage | 38.5% | 61.5% |  |  |  |
|  |  | Lose | Frequency | 25 | 42 |  |  |  |
|  |  |  | Percentage | 37.3% | 62.7% |  |  |  |
| **Low** | **ON-E** | Win | Frequency | 2 | 6 |  | 0.638 (**) | -0.158 |
|  |  |  | Percentage | 25.0% | 75.0% |  |  |  |
|  |  | Lose | Frequency | 4 | 6 |  |  |  |
|  |  |  | Percentage | 40.0% | 60.0% |  |  |  |
| **Middle** | **ON-E** | Win | Frequency | 14 | 19 | 0.907 | 0.341 | -0.108 |
|  |  |  | Percentage | 42.4% | 57.6% |  |  |  |
|  |  | Lose | Frequency | 24 | 21 |  |  |  |
|  |  |  | Percentage | 53.3% | 46.7% |  |  |  |
| **High** | **ON-E** | Win | Frequency | 17 | 27 | 0.112 | 0.737 | -0.038 |
|  |  |  | Percentage | 38.6% | 61.4% |  |  |  |
|  |  | Lose | Frequency | 14 | 19 |  |  |  |
|  |  |  | Percentage | 42.4% | 57.6% |  |  |  |
| **Low** | **OF-U** | Win | Frequency | 5 | 6 |  | 0.670 (**) | -0.145 |
|  |  |  | Percentage | 45.5% | 54.5% |  |  |  |
|  |  | Lose | Frequency | 6 | 4 |  |  |  |
|  |  |  | Percentage | 60.0% | 40.0% |  |  |  |
| **Middle** | **OF-U** | Win | Frequency | 42 | 56 | 0.273 | 0.601 | 0.037 |
|  |  |  | Percentage | 42.9% | 57.1% |  |  |  |
|  |  | Lose | Frequency | 38 | 59 |  |  |  |
|  |  |  | Percentage | 39.2% | 60.8% |  |  |  |
| **High** | **OF-U** | Win | Frequency | 61 | 56 | 3.278 | 0.070 | 0.122 |
|  |  |  | Percentage | 52.1% | 47.9% |  |  |  |
|  |  | Lose | Frequency | 42 | 63 |  |  |  |
|  |  |  | Percentage | 40.0% | 60.0% |  |  |  |
| **Low** | **OF-S** | Win | Frequency | 0 | 1 |  | 0.295 (**) | -0.250 |
|  |  |  | Percentage | 0.0% | 100.0% |  |  |  |
|  |  | Lose | Frequency | 1 | 3 |  |  |  |
|  |  |  | Percentage | 25.0% | 75.0% |  |  |  |
| **Middle** | **OF-S** | Win | Frequency | 9 | 4 |  | 0.417 (**) | 0.195 |
|  |  |  | Percentage | 69.2% | 30.8% |  |  |  |
|  |  | Lose | Frequency | 5 | 5 |  |  |  |
|  |  |  | Percentage | 50.0% | 50.0% |  |  |  |
| **High** | **OF-S** | Win | Frequency | 4 | 0 |  | 0.091 (**) | 0.535 |
|  |  |  | Percentage | 100.0% | 0.0% |  |  |  |
|  |  | Lose | Frequency | 3 | 3 |  |  |  |
|  |  |  | Percentage | 50.0% | 50.0% |  |  |  |

Low, low-point classification; Middle, middle-point classification; High, high-point classification; ON-U, the plays where the user shot using the on-the-ball screen; ON-S, plays where the screener of the on-the-ball screen shot after receiving a pass from the user; ON-A, plays where another player shot after receiving a pass from the user of the on-the-ball screen; ON-E, plays that led to a shot through two or more extra passes after the user used the on-the-ball screen; OF-U, plays where the user shot using the off-the-ball screen; OF-S, plays where the screener of the off-the-ball screen shot.

**p* < 0.05.

**We adopted *p*-value by Fisher's method.

TABLE S35 Differences in shot-success rate of the shooter in movement of on-the-ball screen plays.

| **Shooter** | **Movement of on-the-ball screen plays** |  |  | **Success** | **Fail** | **χ^2^(df=1)** | ***p*** | ***φ*** |
| --- | --- | --- | --- | --- | --- | --- | --- | --- |
| **Low** | **Around** | Win | Frequency | 1 | 2 |  | 1.000 (**) | -0.333 |
|  |  |  | Percentage | 33.3% | 66.7% |  |  |  |
|  |  | Lose | Frequency | 2 | 1 |  |  |  |
|  |  |  | Percentage | 66.7% | 33.3% |  |  |  |
| **Middle** | **Around** | Win | Frequency | 3 | 1 |  | 0.569 (**) | 0.262 |
|  |  |  | Percentage | 75.0% | 25.0% |  |  |  |
|  |  | Lose | Frequency | 5 | 6 |  |  |  |
|  |  |  | Percentage | 45.5% | 54.5% |  |  |  |
| **High** | **Around** | Win | Frequency | 4 | 8 |  | 1.000 (**) | 0.000 |
|  |  |  | Percentage | 33.3% | 66.7% |  |  |  |
|  |  | Lose | Frequency | 2 | 4 |  |  |  |
|  |  |  | Percentage | 33.3% | 66.7% |  |  |  |
| **Low** | **Center-line** | Win | Frequency | 10 | 15 | 0.394 | 0.530 | -0.084 |
|  |  |  | Percentage | 40.0% | 60.0% |  |  |  |
|  |  | Lose | Frequency | 15 | 16 |  |  |  |
|  |  |  | Percentage | 48.4% | 51.6% |  |  |  |
| **Middle** | **Center-line** | Win | Frequency | 58 | 91 | 0.484 | 0.486 | 0.042 |
|  |  |  | Percentage | 38.9% | 61.1% |  |  |  |
|  |  | Lose | Frequency | 45 | 84 |  |  |  |
|  |  |  | Percentage | 34.9% | 65.1% |  |  |  |
| **High** | **Center-line** | Win | Frequency | 62 | 63 | 4.722* | 0.030 | 0.137 |
|  |  |  | Percentage | 49.6% | 50.4% |  |  |  |
|  |  | Lose | Frequency | 45 | 80 |  |  |  |
|  |  |  | Percentage | 36.0% | 64.0% |  |  |  |
| **Low** | **End-line** | Win | Frequency | 8 | 10 | 0.022 | 0.881 | -0.022 |
|  |  |  | Percentage | 44.4% | 55.6% |  |  |  |
|  |  | Lose | Frequency | 14 | 16 |  |  |  |
|  |  |  | Percentage | 46.7% | 53.3% |  |  |  |
| **Middle** | **End-line** | Win | Frequency | 70 | 98 | 0.003 | 0.959 | -0.003 |
|  |  |  | Percentage | 41.7% | 58.3% |  |  |  |
|  |  | Lose | Frequency | 60 | 83 |  |  |  |
|  |  |  | Percentage | 42.0% | 58.0% |  |  |  |
| **High** | **End-line** | Win | Frequency | 70 | 91 | 1.892 | 0.169 | 0.078 |
|  |  |  | Percentage | 43.5% | 56.5% |  |  |  |
|  |  | Lose | Frequency | 53 | 95 |  |  |  |
|  |  |  | Percentage | 35.8% | 64.2% |  |  |  |
| **Low** | **ON-Down** | Win | Frequency | 0 | 2 |  |  |  |
|  |  |  | Percentage | 0.0% | 100.0% |  |  |  |
|  |  | Lose | Frequency | 0 | 1 |  |  |  |
|  |  |  | Percentage | 0.0% | 100.0% |  |  |  |
| **Middle** | **ON-Down** | Win | Frequency | 2 | 6 |  | 0.315 (**) | -0.327 |
|  |  |  | Percentage | 25.0% | 75.0% |  |  |  |
|  |  | Lose | Frequency | 4 | 3 |  |  |  |
|  |  |  | Percentage | 57.1% | 42.9% |  |  |  |
| **High** | **ON-Down** | Win | Frequency | 3 | 13 |  | 0.391 (**) | -0.197 |
|  |  |  | Percentage | 18.8% | 81.3% |  |  |  |
|  |  | Lose | Frequency | 4 | 7 |  |  |  |
|  |  |  | Percentage | 36.4% | 63.6% |  |  |  |

Low, low-point classification; Middle, middle-point classification; High, high-point classification; Around, plays where the screener held a ball; Center-line, plays where the user moved toward the center-line side against the screener; End-line, plays where the user moved toward the end-line side against the screener; ON-Down, plays where the screener was on the center-line side of the defense who protected the user holding a ball in a Top or 3P on Top extension.

**p* < 0.05.

**We adopted *p*-value by Fisher's method.

TABLE S36 Differences in shot-success rate of the shooter in movement of off-the-ball screen plays.

| **Shooter** | **Movement of off-the-ball screen plays** |  |  | **Success** | **Fail** | **χ^2^(df=1)** | ***p*** | ***φ*** |
| --- | --- | --- | --- | --- | --- | --- | --- | --- |
| **Low** | **Back** | Win | Frequency | 1 | 0 |  | 1.000 | 0.500 |
|  |  |  | Percentage | 100.0% | 0.0% |  |  |  |
|  |  | Lose | Frequency | 1 | 1 |  |  |  |
|  |  |  | Percentage | 50.0% | 50.0% |  |  |  |
| **Middle** | **Back** | Win | Frequency | 15 | 13 | 1.309 | 0.253 | 0.149 |
|  |  |  | Percentage | 53.6% | 46.4% |  |  |  |
|  |  | Lose | Frequency | 12 | 19 |  |  |  |
|  |  |  | Percentage | 38.7% | 61.3% |  |  |  |
| **High** | **Back** | Win | Frequency | 13 | 10 | 2.367 | 0.124 | 0.220 |
|  |  |  | Percentage | 56.5% | 43.5% |  |  |  |
|  |  | Lose | Frequency | 9 | 17 |  |  |  |
|  |  |  | Percentage | 34.6% | 65.4% |  |  |  |
| **Low** | **Cross** | Win | Frequency | 0 | 0 |  |  |  |
|  |  |  | Percentage | 0.0% | 0.0% |  |  |  |
|  |  | Lose | Frequency | 1 | 2 |  |  |  |
|  |  |  | Percentage | 33.3% | 66.7% |  |  |  |
| **Middle** | **Cross** | Win | Frequency | 5 | 3 |  | 1.000 (**) | -0.044 |
|  |  |  | Percentage | 62.5% | 37.5% |  |  |  |
|  |  | Lose | Frequency | 6 | 3 |  |  |  |
|  |  |  | Percentage | 66.7% | 33.3% |  |  |  |
| **High** | **Cross** | Win | Frequency | 12 | 5 |  | 1.000 (**) | 0.073 |
|  |  |  | Percentage | 70.6% | 29.4% |  |  |  |
|  |  | Lose | Frequency | 7 | 4 |  |  |  |
|  |  |  | Percentage | 63.6% | 36.4% |  |  |  |
| **Low** | **Down** | Win | Frequency | 0 | 2 |  |  |  |
|  |  |  | Percentage | 0.0% | 100.0% |  |  |  |
|  |  | Lose | Frequency | 0 | 0 |  |  |  |
|  |  |  | Percentage | 0.0% | 0.0% |  |  |  |
| **Middle** | **Down** | Win | Frequency | 11 | 11 | 3.426 | 0.064 | 0.270 |
|  |  |  | Percentage | 50.0% | 50.0% |  |  |  |
|  |  | Lose | Frequency | 6 | 19 |  |  |  |
|  |  |  | Percentage | 24.0% | 76.0% |  |  |  |
| **High** | **Down** | Win | Frequency | 16 | 14 | 2.769 | 0.096 | 0.213 |
|  |  |  | Percentage | 53.3% | 46.7% |  |  |  |
|  |  | Lose | Frequency | 10 | 21 |  |  |  |
|  |  |  | Percentage | 32.3% | 67.7% |  |  |  |
| **Low** | **Flare** | Win | Frequency | 4 | 5 |  | 1.000 (**) | -0.111 |
|  |  |  | Percentage | 44.4% | 55.6% |  |  |  |
|  |  | Lose | Frequency | 5 | 4 |  |  |  |
|  |  |  | Percentage | 55.6% | 44.4% |  |  |  |
| **Middle** | **Flare** | Win | Frequency | 19 | 33 | 0.730 | 0.393 | -0.088 |
|  |  |  | Percentage | 36.5% | 63.5% |  |  |  |
|  |  | Lose | Frequency | 19 | 23 |  |  |  |
|  |  |  | Percentage | 45.2% | 54.8% |  |  |  |
| **High** | **Flare** | Win | Frequency | 24 | 27 | 0.078 | 0.781 | 0.029 |
|  |  |  | Percentage | 47.1% | 52.9% |  |  |  |
|  |  | Lose | Frequency | 19 | 24 |  |  |  |
|  |  |  | Percentage | 44.2% | 55.8% |  |  |  |

Low, low-point classification; Middle, middle-point classification; High, high-point classification; Back, plays where the screener was on the end-line side of the defense who protected the user; Cross, plays where the screener was on the middle-line (the imaginary line connecting baskets running through the center of the court) side of the defense who protected the user; Down, plays where the screener on the center-line side of the defense who protected the user; Flare, plays where the screener on the side-line side of the defense who protected the user.

**We adopted *p*-value by Fisher's method.

TABLE S37 Differences in shot-success rate of the shooter × the user.

| **Shooter** | **User** |  |  | **Success** | **Fail** | **χ^2^(df=1)** | ***p*** | ***φ*** |
| --- | --- | --- | --- | --- | --- | --- | --- | --- |
| **Low** | **Low** | Win | Frequency | 5 | 10 | 0.354 | 0.552 | -0.107 |
|  |  |  | Percentage | 33.3% | 66.7% |  |  |  |
|  |  | Lose | Frequency | 7 | 9 |  |  |  |
|  |  |  | Percentage | 43.8% | 56.3% |  |  |  |
| **Low** | **Middle** | Win | Frequency | 13 | 12 | 0.012 | 0.914 | 0.018 |
|  |  |  | Percentage | 52.0% | 48.0% |  |  |  |
|  |  | Lose | Frequency | 7 | 6 |  |  |  |
|  |  |  | Percentage | 53.8% | 46.2% |  |  |  |
| **Low** | **High** | Win | Frequency | 12 | 20 | 0.691 | 0.406 | -0.099 |
|  |  |  | Percentage | 37.5% | 62.5% |  |  |  |
|  |  | Lose | Frequency | 18 | 20 |  |  |  |
|  |  |  | Percentage | 47.4% | 52.6% |  |  |  |
| **Middle** | **Low** | Win | Frequency | 1 | 1 |  | 1.000 (**) | 0.250 |
|  |  |  | Percentage | 50.0% | 50.0% |  |  |  |
|  |  | Lose | Frequency | 1 | 3 |  |  |  |
|  |  |  | Percentage | 25.0% | 75.0% |  |  |  |
| **Middle** | **Middle** | Win | Frequency | 136 | 191 | 1.022 | 0.312 | 0.040 |
|  |  |  | Percentage | 41.6% | 58.4% |  |  |  |
|  |  | Lose | Frequency | 116 | 192 |  |  |  |
|  |  |  | Percentage | 37.7% | 62.3% |  |  |  |
| **Middle** | **High** | Win | Frequency | 47 | 64 | 0.434 | 0.510 | -0.047 |
|  |  |  | Percentage | 42.3% | 57.7% |  |  |  |
|  |  | Lose | Frequency | 40 | 45 |  |  |  |
|  |  |  | Percentage | 47.1% | 52.9% |  |  |  |
| **High** | **Low** | Win | Frequency | 1 | 1 |  | 1.000 (**) | -0.091 |
|  |  |  | Percentage | 50.0% | 50.0% |  |  |  |
|  |  | Lose | Frequency | 3 | 2 |  |  |  |
|  |  |  | Percentage | 60.0% | 40.0% |  |  |  |
| **High** | **Middle** | Win | Frequency | 30 | 39 | 0.598 | 0.439 | 0.067 |
|  |  |  | Percentage | 43.5% | 56.5% |  |  |  |
|  |  | Lose | Frequency | 24 | 41 |  |  |  |
|  |  |  | Percentage | 36.9% | 63.1% |  |  |  |
| **High** | **High** | Win | Frequency | 174 | 191 | 8.304* | 0.004 | 0.109 |
|  |  |  | Percentage | 47.7% | 52.3% |  |  |  |
|  |  | Lose | Frequency | 122 | 209 |  |  |  |
|  |  |  | Percentage | 36.9% | 63.1% |  |  |  |

Low, low-point classification; Middle, middle-point classification; High, high-point classification.

**p* < 0.05.

**We adopted *p*-value by Fisher's method.

TABLE S38 Differences in shot-success rate of the shooter × the screener.

| **Shooter** | **Screener** |  |  | **Success** | **Fail** | **χ^2^(df=1)** | ***p*** | ***φ*** |
| --- | --- | --- | --- | --- | --- | --- | --- | --- |
| **Low** | **Low** | Win | Frequency | 7 | 11 | 0.450 | 0.502 | -0.092 |
|  |  |  | Percentage | 38.9% | 61.1% |  |  |  |
|  |  | Lose | Frequency | 17 | 18 |  |  |  |
|  |  |  | Percentage | 48.6% | 51.4% |  |  |  |
| **Low** | **Middle** | Win | Frequency | 9 | 15 | 2.407 | 0.121 | -0.217 |
|  |  |  | Percentage | 37.5% | 62.5% |  |  |  |
|  |  | Lose | Frequency | 16 | 11 |  |  |  |
|  |  |  | Percentage | 59.3% | 40.7% |  |  |  |
| **Low** | **High** | Win | Frequency | 7 | 9 | 0.732 | 0.392 | 0.149 |
|  |  |  | Percentage | 43.8% | 56.3% |  |  |  |
|  |  | Lose | Frequency | 5 | 12 |  |  |  |
|  |  |  | Percentage | 29.4% | 70.6% |  |  |  |
| **Middle** | **Low** | Win | Frequency | 46 | 51 | 0.718 | 0.397 | 0.059 |
|  |  |  | Percentage | 47.4% | 52.6% |  |  |  |
|  |  | Lose | Frequency | 44 | 62 |  |  |  |
|  |  |  | Percentage | 41.5% | 58.5% |  |  |  |
| **Middle** | **Middle** | Win | Frequency | 89 | 140 | 0.014 | 0.905 | -0.006 |
|  |  |  | Percentage | 38.9% | 61.1% |  |  |  |
|  |  | Lose | Frequency | 82 | 126 |  |  |  |
|  |  |  | Percentage | 39.4% | 60.6% |  |  |  |
| **Middle** | **High** | Win | Frequency | 48 | 61 | 0.941 | 0.332 | 0.070 |
|  |  |  | Percentage | 44.0% | 56.0% |  |  |  |
|  |  | Lose | Frequency | 30 | 51 |  |  |  |
|  |  |  | Percentage | 37.0% | 63.0% |  |  |  |
| **High** | **Low** | Win | Frequency | 60 | 62 | 1.981 | 0.159 | 0.086 |
|  |  |  | Percentage | 49.2% | 50.8% |  |  |  |
|  |  | Lose | Frequency | 58 | 85 |  |  |  |
|  |  |  | Percentage | 40.6% | 59.4% |  |  |  |
| **High** | **Middle** | Win | Frequency | 97 | 107 | 5.503* | 0.019 | 0.121 |
|  |  |  | Percentage | 47.5% | 52.5% |  |  |  |
|  |  | Lose | Frequency | 60 | 109 |  |  |  |
|  |  |  | Percentage | 35.5% | 64.5% |  |  |  |
| **High** | **High** | Win | Frequency | 45 | 55 | 1.796 | 0.180 | 0.099 |
|  |  |  | Percentage | 45.0% | 55.0% |  |  |  |
|  |  | Lose | Frequency | 30 | 55 |  |  |  |
|  |  |  | Percentage | 35.3% | 64.7% |  |  |  |

Low, low-point classification; Middle, middle-point classification; High, high-point classification.

**p* < 0.05.

TABLE S39 Differences in shot-success rate of the shooter × the passer.

| **Shooter** | **Passer** |  |  | **Success** | **Fail** | **χ^2^(df=1)** | ***p*** | ***φ*** |
| --- | --- | --- | --- | --- | --- | --- | --- | --- |
| **Low** | **Low** | Win | Frequency | 0 | 0 |  |  |  |
|  |  |  | Percentage | 0.0% | 0.0% |  |  |  |
|  |  | Lose | Frequency | 0 | 1 |  |  |  |
|  |  |  | Percentage | 0.0% | 100.0% |  |  |  |
| **Low** | **Middle** | Win | Frequency | 8 | 12 | 0.878 | 0.349 | -0.141 |
|  |  |  | Percentage | 40.0% | 60.0% |  |  |  |
|  |  | Lose | Frequency | 13 | 11 |  |  |  |
|  |  |  | Percentage | 54.2% | 45.8% |  |  |  |
| **Low** | **High** | Win | Frequency | 16 | 20 | 0.171 | 0.679 | -0.045 |
|  |  |  | Percentage | 44.4% | 55.6% |  |  |  |
|  |  | Lose | Frequency | 24 | 25 |  |  |  |
|  |  |  | Percentage | 49.0% | 51.0% |  |  |  |
| **Middle** | **Low** | Win | Frequency | 3 | 7 |  | 0.414 (**) | -0.230 |
|  |  |  | Percentage | 30.0% | 70.0% |  |  |  |
|  |  | Lose | Frequency | 8 | 7 |  |  |  |
|  |  |  | Percentage | 53.3% | 46.7% |  |  |  |
| **Middle** | **Middle** | Win | Frequency | 44 | 54 | 0.164 | 0.686 | -0.028 |
|  |  |  | Percentage | 44.9% | 55.1% |  |  |  |
|  |  | Lose | Frequency | 52 | 57 |  |  |  |
|  |  |  | Percentage | 47.7% | 52.3% |  |  |  |
| **Middle** | **High** | Win | Frequency | 75 | 97 | 0.495 | 0.482 | 0.039 |
|  |  |  | Percentage | 43.6% | 56.4% |  |  |  |
|  |  | Lose | Frequency | 60 | 91 |  |  |  |
|  |  |  | Percentage | 39.7% | 60.3% |  |  |  |
| **High** | **Low** | Win | Frequency | 5 | 7 |  | 1.000 (**) | 0.048 |
|  |  |  | Percentage | 41.7% | 58.3% |  |  |  |
|  |  | Lose | Frequency | 7 | 12 |  |  |  |
|  |  |  | Percentage | 36.8% | 63.2% |  |  |  |
| **High** | **Middle** | Win | Frequency | 69 | 77 | 0.943 | 0.332 | 0.060 |
|  |  |  | Percentage | 47.3% | 52.7% |  |  |  |
|  |  | Lose | Frequency | 47 | 67 |  |  |  |
|  |  |  | Percentage | 41.2% | 58.8% |  |  |  |
| **High** | **High** | Win | Frequency | 42 | 46 | 1.295 | 0.255 | 0.084 |
|  |  |  | Percentage | 47.7% | 52.3% |  |  |  |
|  |  | Lose | Frequency | 37 | 57 |  |  |  |
|  |  |  | Percentage | 39.4% | 60.6% |  |  |  |

Low, low-point classification; Middle, middle-point classification; High, high-point classification.

**We adopted *p*-value by Fisher's method.

TABLE S40 Differences in shot-success rate depending on the PC of the user.

| **User** |  |  | **Success** | **Fail** | **χ^2^(df=1)** | ***p*** | ***φ*** |
| --- | --- | --- | --- | --- | --- | --- | --- |
| **Low** | Win | Frequency | 7 | 12 | 0.229 | 0.632 | -0.072 |
|  |  | Percentage | 36.8% | 63.2% |  |  |  |
|  | Lose | Frequency | 11 | 14 |  |  |  |
|  |  | Percentage | 44.0% | 56.0% |  |  |  |
| **Middle** | Win | Frequency | 173 | 236 | 1.246 | 0.264 | 0.039 |
|  |  | Percentage | 42.3% | 57.7% |  |  |  |
|  | Lose | Frequency | 153 | 245 |  |  |  |
|  |  | Percentage | 38.4% | 61.6% |  |  |  |
| **High** | Win | Frequency | 233 | 275 | 3.784 | 0.052 | 0.063 |
|  |  | Percentage | 45.9% | 54.1% |  |  |  |
|  | Lose | Frequency | 180 | 274 |  |  |  |
|  |  | Percentage | 39.6% | 60.4% |  |  |  |

PC, player classification; Low, low-point classification; Middle, middle-point classification; High, high-point classification.

TABLE S41 Differences in shot-success rate of the user in shot location.

| **User** | **Shot Location** |  |  | **Success** | **Fail** | **χ^2^(df=1)** | ***p*** | ***φ*** |
| --- | --- | --- | --- | --- | --- | --- | --- | --- |
| **Low** | **PL** | Win | Frequency | 0 | 1 |  | 1.000 (**) | -0.447 |
|  |  |  | Percentage | 0.0% | 100.0% |  |  |  |
|  |  | Lose | Frequency | 3 | 2 |  |  |  |
|  |  |  | Percentage | 60.0% | 40.0% |  |  |  |
| **Middle** | **PL** | Win | Frequency | 55 | 43 | 0.024 | 0.876 | 0.011 |
|  |  |  | Percentage | 56.1% | 43.9% |  |  |  |
|  |  | Lose | Frequency | 60 | 49 |  |  |  |
|  |  |  | Percentage | 55.0% | 45.0% |  |  |  |
| **High** | **PL** | Win | Frequency | 87 | 63 | 0.040 | 0.841 | 0.012 |
|  |  |  | Percentage | 58.0% | 42.0% |  |  |  |
|  |  | Lose | Frequency | 71 | 54 |  |  |  |
|  |  |  | Percentage | 56.8% | 43.2% |  |  |  |
| **Low** | **PH** | Win | Frequency | 0 | 0 |  |  |  |
|  |  |  | Percentage | 0.0% | 0.0% |  |  |  |
|  |  | Lose | Frequency | 0 | 2 |  |  |  |
|  |  |  | Percentage | 0.0% | 100.0% |  |  |  |
| **Middle** | **PH** | Win | Frequency | 13 | 14 | 2.270 | 0.132 | 0.184 |
|  |  |  | Percentage | 48.1% | 51.9% |  |  |  |
|  |  | Lose | Frequency | 12 | 28 |  |  |  |
|  |  |  | Percentage | 30.0% | 70.0% |  |  |  |
| **High** | **PH** | Win | Frequency | 24 | 23 | 6.206* | 0.013 | 0.272 |
|  |  |  | Percentage | 51.1% | 48.9% |  |  |  |
|  |  | Lose | Frequency | 9 | 28 |  |  |  |
|  |  |  | Percentage | 24.3% | 75.7% |  |  |  |
| **Low** | **Top** | Win | Frequency | 0 | 1 |  | 1.000 (**) | -0.408 |
|  |  |  | Percentage | 0.0% | 100.0% |  |  |  |
|  |  | Lose | Frequency | 2 | 2 |  |  |  |
|  |  |  | Percentage | 50.0% | 50.0% |  |  |  |
| **Middle** | **Top** | Win | Frequency | 14 | 23 | 0.003 | 0.955 | -0.006 |
|  |  |  | Percentage | 37.8% | 62.2% |  |  |  |
|  |  | Lose | Frequency | 15 | 24 |  |  |  |
|  |  |  | Percentage | 38.5% | 61.5% |  |  |  |
| **High** | **Top** | Win | Frequency | 21 | 32 | 0.146 | 0.703 | -0.040 |
|  |  |  | Percentage | 39.6% | 60.4% |  |  |  |
|  |  | Lose | Frequency | 17 | 22 |  |  |  |
|  |  |  | Percentage | 43.6% | 56.4% |  |  |  |
| **Low** | **Corner** | Win | Frequency | 5 | 5 |  | 1.000 (**) | 0.101 |
|  |  |  | Percentage | 50.0% | 50.0% |  |  |  |
|  |  | Lose | Frequency | 4 | 6 |  |  |  |
|  |  |  | Percentage | 40.0% | 60.0% |  |  |  |
| **Middle** | **Corner** | Win | Frequency | 40 | 53 | 0.012 | 0.912 | -0.008 |
|  |  |  | Percentage | 43.0% | 57.0% |  |  |  |
|  |  | Lose | Frequency | 39 | 50 |  |  |  |
|  |  |  | Percentage | 43.8% | 56.2% |  |  |  |
| **High** | **Corner** | Win | Frequency | 45 | 54 | 0.036 | 0.849 | 0.014 |
|  |  |  | Percentage | 45.5% | 54.5% |  |  |  |
|  |  | Lose | Frequency | 41 | 52 |  |  |  |
|  |  |  | Percentage | 44.1% | 55.9% |  |  |  |
| **Low** | **Wing** | Win | Frequency | 2 | 4 |  | 0.524 (**) | -0.316 |
|  |  |  | Percentage | 33.3% | 66.7% |  |  |  |
|  |  | Lose | Frequency | 2 | 1 |  |  |  |
|  |  |  | Percentage | 66.7% | 33.3% |  |  |  |
| **Middle** | **Wing** | Win | Frequency | 37 | 63 | 3.838 | 0.050 | 0.150 |
|  |  |  | Percentage | 37.0% | 63.0% |  |  |  |
|  |  | Lose | Frequency | 16 | 54 |  |  |  |
|  |  |  | Percentage | 22.9% | 77.1% |  |  |  |
| **High** | **Wing** | Win | Frequency | 31 | 59 | 0.008 | 0.929 | 0.007 |
|  |  |  | Percentage | 34.4% | 65.6% |  |  |  |
|  |  | Lose | Frequency | 25 | 49 |  |  |  |
|  |  |  | Percentage | 33.8% | 66.2% |  |  |  |
| **Low** | **3P** | Win | Frequency | 0 | 1 |  |  |  |
|  |  |  | Percentage | 0.0% | 100.0% |  |  |  |
|  |  | Lose | Frequency | 0 | 1 |  |  |  |
|  |  |  | Percentage | 0.0% | 100.0% |  |  |  |
| **Middle** | **3P** | Win | Frequency | 14 | 40 | 0.275 | 0.600 | 0.051 |
|  |  |  | Percentage | 25.9% | 74.1% |  |  |  |
|  |  | Lose | Frequency | 11 | 40 |  |  |  |
|  |  |  | Percentage | 21.6% | 78.4% |  |  |  |
| **High** | **3P** | Win | Frequency | 25 | 44 | 5.253* | 0.022 | 0.184 |
|  |  |  | Percentage | 36.2% | 63.8% |  |  |  |
|  |  | Lose | Frequency | 17 | 69 |  |  |  |
|  |  |  | Percentage | 19.8% | 80.2% |  |  |  |

Low, low-point classification; Middle, middle-point classification; High, high-point classification; PL, paint-low; PH, paint-high; 3P, 3-point field goal area.

**p* < 0.05.

**We adopted *p*-value by Fisher's method.

TABLE S42 Differences in shot-success rate of the user in screen location.

| **User** | **Screen Location** |  |  | **Success** | **Fail** | **χ^2^(df=1)** | ***p*** | ***φ*** |
| --- | --- | --- | --- | --- | --- | --- | --- | --- |
| **Low** | **PL** | Win | Frequency | 0 | 0 |  |  |  |
|  |  |  | Percentage | 0.0% | 0.0% |  |  |  |
|  |  | Lose | Frequency | 2 | 1 |  |  |  |
|  |  |  | Percentage | 66.7% | 33.3% |  |  |  |
| **Middle** | **PL** | Win | Frequency | 14 | 7 | 1.588 | 0.208 | 0.190 |
|  |  |  | Percentage | 66.7% | 33.3% |  |  |  |
|  |  | Lose | Frequency | 11 | 12 |  |  |  |
|  |  |  | Percentage | 47.8% | 52.2% |  |  |  |
| **High** | **PL** | Win | Frequency | 21 | 11 | 0.696 | 0.404 | 0.107 |
|  |  |  | Percentage | 65.6% | 34.4% |  |  |  |
|  |  | Lose | Frequency | 16 | 13 |  |  |  |
|  |  |  | Percentage | 55.2% | 44.8% |  |  |  |
| **Low** | **PH** | Win | Frequency | 0 | 1 |  |  |  |
|  |  |  | Percentage | 0.0% | 100.0% |  |  |  |
|  |  | Lose | Frequency | 0 | 1 |  |  |  |
|  |  |  | Percentage | 0.0% | 100.0% |  |  |  |
| **Middle** | **PH** | Win | Frequency | 17 | 16 | 1.150 | 0.284 | 0.136 |
|  |  |  | Percentage | 51.5% | 48.5% |  |  |  |
|  |  | Lose | Frequency | 11 | 18 |  |  |  |
|  |  |  | Percentage | 37.9% | 62.1% |  |  |  |
| **High** | **PH** | Win | Frequency | 21 | 34 | 0.522 | 0.470 | -0.079 |
|  |  |  | Percentage | 38.2% | 61.8% |  |  |  |
|  |  | Lose | Frequency | 13 | 15 |  |  |  |
|  |  |  | Percentage | 46.4% | 53.6% |  |  |  |
| **Low** | **Top** | Win | Frequency | 0 | 0 |  |  |  |
|  |  |  | Percentage | 0.0% | 0.0% |  |  |  |
|  |  | Lose | Frequency | 0 | 3 |  |  |  |
|  |  |  | Percentage | 0.0% | 100.0% |  |  |  |
| **Middle** | **Top** | Win | Frequency | 12 | 15 | 1.459 | 0.227 | 0.144 |
|  |  |  | Percentage | 44.4% | 55.6% |  |  |  |
|  |  | Lose | Frequency | 13 | 30 |  |  |  |
|  |  |  | Percentage | 30.2% | 69.8% |  |  |  |
| **High** | **Top** | Win | Frequency | 16 | 27 | 0.022 | 0.883 | 0.014 |
|  |  |  | Percentage | 37.2% | 62.8% |  |  |  |
|  |  | Lose | Frequency | 24 | 43 |  |  |  |
|  |  |  | Percentage | 35.8% | 64.2% |  |  |  |
| **Low** | **Corner** | Win | Frequency | 3 | 5 |  | 0.650 (**) | -0.204 |
|  |  |  | Percentage | 37.5% | 62.5% |  |  |  |
|  |  | Lose | Frequency | 7 | 5 |  |  |  |
|  |  |  | Percentage | 58.3% | 41.7% |  |  |  |
| **Middle** | **Corner** | Win | Frequency | 51 | 62 | 0.022 | 0.882 | 0.010 |
|  |  |  | Percentage | 45.1% | 54.9% |  |  |  |
|  |  | Lose | Frequency | 49 | 62 |  |  |  |
|  |  |  | Percentage | 44.1% | 55.9% |  |  |  |
| **High** | **Corner** | Win | Frequency | 71 | 68 | 3.487 | 0.062 | 0.120 |
|  |  |  | Percentage | 51.1% | 48.9% |  |  |  |
|  |  | Lose | Frequency | 41 | 64 |  |  |  |
|  |  |  | Percentage | 39.0% | 61.0% |  |  |  |
| **Low** | **Wing** | Win | Frequency | 3 | 5 |  | 1.000 (**) | -0.102 |
|  |  |  | Percentage | 37.5% | 62.5% |  |  |  |
|  |  | Lose | Frequency | 1 | 1 |  |  |  |
|  |  |  | Percentage | 50.0% | 50.0% |  |  |  |
| **Middle** | **Wing** | Win | Frequency | 68 | 119 | 0.127 | 0.722 | 0.019 |
|  |  |  | Percentage | 36.4% | 63.6% |  |  |  |
|  |  | Lose | Frequency | 57 | 108 |  |  |  |
|  |  |  | Percentage | 34.5% | 65.5% |  |  |  |
| **High** | **Wing** | Win | Frequency | 84 | 116 | 1.315 | 0.252 | 0.059 |
|  |  |  | Percentage | 42.0% | 58.0% |  |  |  |
|  |  | Lose | Frequency | 66 | 116 |  |  |  |
|  |  |  | Percentage | 36.3% | 63.7% |  |  |  |
| **Low** | **3P** | Win | Frequency | 0 | 0 |  |  |  |
|  |  |  | Percentage | 0.0% | 0.0% |  |  |  |
|  |  | Lose | Frequency | 1 | 3 |  |  |  |
|  |  |  | Percentage | 25.0% | 75.0% |  |  |  |
| **Middle** | **3P** | Win | Frequency | 10 | 13 | 0.001 | 0.971 | -0.005 |
|  |  |  | Percentage | 43.5% | 56.5% |  |  |  |
|  |  | Lose | Frequency | 11 | 14 |  |  |  |
|  |  |  | Percentage | 44.0% | 56.0% |  |  |  |
| **High** | **3P** | Win | Frequency | 17 | 12 | 0.655 | 0.418 | 0.098 |
|  |  |  | Percentage | 58.6% | 41.4% |  |  |  |
|  |  | Lose | Frequency | 19 | 20 |  |  |  |
|  |  |  | Percentage | 48.7% | 51.3% |  |  |  |

Low, low-point classification; Middle, middle-point classification; High, high-point classification; PL, paint-low; PH, paint-high; 3P, 3-point field goal area.

**We adopted *p*-value by Fisher's method.

TABLE S43 Differences in shot-success rate of the user in pass location.

| **User** | **Pass Location** |  |  | **Success** | **Fail** | **χ^2^(df=1)** | ***p*** | ***φ*** |
| --- | --- | --- | --- | --- | --- | --- | --- | --- |
| **Low** | **PL** | Win | Frequency | 0 | 0 |  |  |  |
|  |  |  | Percentage | 0.0% | 0.0% |  |  |  |
|  |  | Lose | Frequency | 0 | 1 |  |  |  |
|  |  |  | Percentage | 0.0% | 100.0% |  |  |  |
| **Middle** | **PL** | Win | Frequency | 6 | 4 |  | 1.000 (**) | 0.045 |
|  |  |  | Percentage | 60.0% | 40.0% |  |  |  |
|  |  | Lose | Frequency | 5 | 4 |  |  |  |
|  |  |  | Percentage | 55.6% | 44.4% |  |  |  |
| **High** | **PL** | Win | Frequency | 5 | 7 |  | 1.000 (**) | -0.059 |
|  |  |  | Percentage | 41.7% | 58.3% |  |  |  |
|  |  | Lose | Frequency | 1 | 1 |  |  |  |
|  |  |  | Percentage | 50.0% | 50.0% |  |  |  |
| **Low** | **PH** | Win | Frequency | 0 | 2 |  | 1.000 (**) | -0.577 |
|  |  |  | Percentage | 0.0% | 100.0% |  |  |  |
|  |  | Lose | Frequency | 1 | 1 |  |  |  |
|  |  |  | Percentage | 50.0% | 50.0% |  |  |  |
| **Middle** | **PH** | Win | Frequency | 12 | 18 | 1.200 | 0.273 | 0.141 |
|  |  |  | Percentage | 40.0% | 60.0% |  |  |  |
|  |  | Lose | Frequency | 8 | 22 |  |  |  |
|  |  |  | Percentage | 26.7% | 73.3% |  |  |  |
| **High** | **PH** | Win | Frequency | 29 | 24 | 0.482 | 0.487 | 0.074 |
|  |  |  | Percentage | 54.7% | 45.3% |  |  |  |
|  |  | Lose | Frequency | 17 | 19 |  |  |  |
|  |  |  | Percentage | 47.2% | 52.8% |  |  |  |
| **Low** | **Top** | Win | Frequency | 5 | 4 |  | 1.000 (**) | -0.016 |
|  |  |  | Percentage | 55.6% | 44.4% |  |  |  |
|  |  | Lose | Frequency | 4 | 3 |  |  |  |
|  |  |  | Percentage | 57.1% | 42.9% |  |  |  |
| **Middle** | **Top** | Win | Frequency | 20 | 27 | 2.263 | 0.132 | -0.151 |
|  |  |  | Percentage | 42.6% | 57.4% |  |  |  |
|  |  | Lose | Frequency | 30 | 22 |  |  |  |
|  |  |  | Percentage | 57.7% | 42.3% |  |  |  |
| **High** | **Top** | Win | Frequency | 14 | 25 | 0.816 | 0.366 | -0.094 |
|  |  |  | Percentage | 35.9% | 64.1% |  |  |  |
|  |  | Lose | Frequency | 24 | 29 |  |  |  |
|  |  |  | Percentage | 45.3% | 54.7% |  |  |  |
| **Low** | **Corner** | Win | Frequency | 0 | 1 | 2.000 | 0.157 | -1.000 |
|  |  |  | Percentage | 0.0% | 100.0% |  |  |  |
|  |  | Lose | Frequency | 1 | 0 |  |  |  |
|  |  |  | Percentage | 100.0% | 0.0% |  |  |  |
| **Middle** | **Corner** | Win | Frequency | 24 | 31 | 0.044 | 0.833 | -0.020 |
|  |  |  | Percentage | 43.6% | 56.4% |  |  |  |
|  |  | Lose | Frequency | 26 | 31 |  |  |  |
|  |  |  | Percentage | 45.6% | 54.4% |  |  |  |
| **High** | **Corner** | Win | Frequency | 31 | 43 | 0.038 | 0.845 | 0.018 |
|  |  |  | Percentage | 41.9% | 58.1% |  |  |  |
|  |  | Lose | Frequency | 16 | 24 |  |  |  |
|  |  |  | Percentage | 40.0% | 60.0% |  |  |  |
| **Low** | **Wing** | Win | Frequency | 0 | 0 |  |  |  |
|  |  |  | Percentage | 0.0% | 0.0% |  |  |  |
|  |  | Lose | Frequency | 3 | 1 |  |  |  |
|  |  |  | Percentage | 75.0% | 25.0% |  |  |  |
| **Middle** | **Wing** | Win | Frequency | 32 | 39 | 0.522 | 0.470 | 0.059 |
|  |  |  | Percentage | 45.1% | 54.9% |  |  |  |
|  |  | Lose | Frequency | 31 | 48 |  |  |  |
|  |  |  | Percentage | 39.2% | 60.8% |  |  |  |
| **High** | **Wing** | Win | Frequency | 37 | 51 | 0.809 | 0.368 | -0.068 |
|  |  |  | Percentage | 42.0% | 58.0% |  |  |  |
|  |  | Lose | Frequency | 42 | 44 |  |  |  |
|  |  |  | Percentage | 48.8% | 51.2% |  |  |  |
| **Low** | **3P** | Win | Frequency | 1 | 0 |  | 0.333 (**) | 0.632 |
|  |  |  | Percentage | 100.0% | 0.0% |  |  |  |
|  |  | Lose | Frequency | 1 | 4 |  |  |  |
|  |  |  | Percentage | 20.0% | 80.0% |  |  |  |
| **Middle** | **3P** | Win | Frequency | 17 | 18 | 1.962 | 0.161 | 0.154 |
|  |  |  | Percentage | 48.6% | 51.4% |  |  |  |
|  |  | Lose | Frequency | 16 | 32 |  |  |  |
|  |  |  | Percentage | 33.3% | 66.7% |  |  |  |
| **High** | **3P** | Win | Frequency | 29 | 26 | 4.068* | 0.044 | 0.185 |
|  |  |  | Percentage | 52.7% | 47.3% |  |  |  |
|  |  | Lose | Frequency | 22 | 42 |  |  |  |
|  |  |  | Percentage | 34.4% | 65.6% |  |  |  |

Low, low-point classification; Middle, middle-point classification; High, high-point classification; PL, paint-low; PH, paint-high; 3P, 3-point field goal area.

**p* < 0.05.

**We adopted *p*-value by Fisher's method.

TABLE S44 Differences in shot-success rate of the user in type of screen.

| **User** | **Type of Screen** |  |  | **Success** | **Fail** | **χ^2^(df=1)** | ***p*** | ***φ*** |
| --- | --- | --- | --- | --- | --- | --- | --- | --- |
| **Low** | **On-the-ball screen** | Win | Frequency | 1 | 6 |  | 1.000 (**) | -0.127 |
|  |  |  | Percentage | 14.3% | 85.7% |  |  |  |
|  |  | Lose | Frequency | 3 | 9 |  |  |  |
|  |  |  | Percentage | 25.0% | 75.0% |  |  |  |
| **Middle** | **On-the-ball screen** | Win | Frequency | 122 | 178 | 0.577 | 0.448 | 0.031 |
|  |  |  | Percentage | 40.7% | 59.3% |  |  |  |
|  |  | Lose | Frequency | 111 | 184 |  |  |  |
|  |  |  | Percentage | 37.6% | 62.4% |  |  |  |
| **High** | **On-the-ball screen** | Win | Frequency | 169 | 216 | 1.156 | 0.282 | 0.040 |
|  |  |  | Percentage | 43.9% | 56.1% |  |  |  |
|  |  | Lose | Frequency | 135 | 203 |  |  |  |
|  |  |  | Percentage | 39.9% | 60.1% |  |  |  |
| **Low** | **Off-the-ball screen** | Win | Frequency | 6 | 6 | 0.337 | 0.561 | -0.116 |
|  |  |  | Percentage | 50.0% | 50.0% |  |  |  |
|  |  | Lose | Frequency | 8 | 5 |  |  |  |
|  |  |  | Percentage | 61.5% | 38.5% |  |  |  |
| **Middle** | **Off-the-ball screen** | Win | Frequency | 51 | 58 | 0.777 | 0.378 | 0.061 |
|  |  |  | Percentage | 46.8% | 53.2% |  |  |  |
|  |  | Lose | Frequency | 42 | 61 |  |  |  |
|  |  |  | Percentage | 40.8% | 59.2% |  |  |  |
| **High** | **Off-the-ball screen** | Win | Frequency | 64 | 59 | 4.218* | 0.040 | 0.133 |
|  |  |  | Percentage | 52.0% | 48.0% |  |  |  |
|  |  | Lose | Frequency | 45 | 71 |  |  |  |
|  |  |  | Percentage | 38.8% | 61.2% |  |  |  |

Low, low-point classification; Middle, middle-point classification; High, high-point classification.

**p* < 0.05.

**We adopted *p*-value by Fisher's method.

TABLE S45 Differences in shot-success rate of the user in type of screen-play.

| **User** | **Type of Screen-play** |  |  | **Success** | **Fail** | **χ^2^(df=1)** | ***p*** | ***φ*** |
| --- | --- | --- | --- | --- | --- | --- | --- | --- |
| **Low** | **ON-U** | Win | Frequency | 0 | 4 |  | 1.000 (**) | -0.272 |
|  |  |  | Percentage | 0.0% | 100.0% |  |  |  |
|  |  | Lose | Frequency | 1 | 5 |  |  |  |
|  |  |  | Percentage | 16.7% | 83.3% |  |  |  |
| **Middle** | **ON-U** | Win | Frequency | 63 | 99 | 1.468 | 0.226 | 0.071 |
|  |  |  | Percentage | 38.9% | 61.1% |  |  |  |
|  |  | Lose | Frequency | 42 | 89 |  |  |  |
|  |  |  | Percentage | 32.1% | 67.9% |  |  |  |
| **High** | **ON-U** | Win | Frequency | 91 | 103 | 6.638* | 0.010 | 0.134 |
|  |  |  | Percentage | 46.9% | 53.1% |  |  |  |
|  |  | Lose | Frequency | 59 | 116 |  |  |  |
|  |  |  | Percentage | 33.7% | 66.3% |  |  |  |
| **Low** | **ON-S** | Win | Frequency | 1 | 1 |  | 1.000 (**) | 0.577 |
|  |  |  | Percentage | 50.0% | 50.0% |  |  |  |
|  |  | Lose | Frequency | 0 | 2 |  |  |  |
|  |  |  | Percentage | 0.0% | 100.0% |  |  |  |
| **Middle** | **ON-S** | Win | Frequency | 12 | 20 | 3.065 | 0.080 | -0.219 |
|  |  |  | Percentage | 37.5% | 62.5% |  |  |  |
|  |  | Lose | Frequency | 19 | 13 |  |  |  |
|  |  |  | Percentage | 59.4% | 40.6% |  |  |  |
| **High** | **ON-S** | Win | Frequency | 18 | 17 | 0.342 | 0.559 | -0.069 |
|  |  |  | Percentage | 51.4% | 48.6% |  |  |  |
|  |  | Lose | Frequency | 21 | 15 |  |  |  |
|  |  |  | Percentage | 58.3% | 41.7% |  |  |  |
| **Low** | **ON-A** | Win | Frequency | 0 | 1 |  | 1.000 (**) | -0.333 |
|  |  |  | Percentage | 0.0% | 100.0% |  |  |  |
|  |  | Lose | Frequency | 1 | 2 |  |  |  |
|  |  |  | Percentage | 33.3% | 66.7% |  |  |  |
| **Middle** | **ON-A** | Win | Frequency | 29 | 32 | 2.802 | 0.094 | 0.140 |
|  |  |  | Percentage | 47.5% | 52.5% |  |  |  |
|  |  | Lose | Frequency | 28 | 55 |  |  |  |
|  |  |  | Percentage | 33.7% | 66.3% |  |  |  |
| **High** | **ON-A** | Win | Frequency | 45 | 71 | 0.058 | 0.810 | -0.017 |
|  |  |  | Percentage | 38.8% | 61.2% |  |  |  |
|  |  | Lose | Frequency | 36 | 53 |  |  |  |
|  |  |  | Percentage | 40.4% | 59.6% |  |  |  |
| **Low** | **ON-E** | Win | Frequency | 0 | 0 |  |  |  |
|  |  |  | Percentage | 0.0% | 0.0% |  |  |  |
|  |  | Lose | Frequency | 1 | 0 |  |  |  |
|  |  |  | Percentage | 100.0% | 0.0% |  |  |  |
| **Middle** | **ON-E** | Win | Frequency | 18 | 27 | 0.230 | 0.631 | -0.049 |
|  |  |  | Percentage | 40.0% | 60.0% |  |  |  |
|  |  | Lose | Frequency | 22 | 27 |  |  |  |
|  |  |  | Percentage | 44.9% | 55.1% |  |  |  |
| **High** | **ON-E** | Win | Frequency | 15 | 25 | 1.238 | 0.266 | -0.126 |
|  |  |  | Percentage | 37.5% | 62.5% |  |  |  |
|  |  | Lose | Frequency | 19 | 19 |  |  |  |
|  |  |  | Percentage | 50.0% | 50.0% |  |  |  |
| **Low** | **OF-U** | Win | Frequency | 5 | 6 |  | 0.670 (**) | -0.145 |
|  |  |  | Percentage | 45.5% | 54.5% |  |  |  |
|  |  | Lose | Frequency | 6 | 4 |  |  |  |
|  |  |  | Percentage | 60.0% | 40.0% |  |  |  |
| **Middle** | **OF-U** | Win | Frequency | 42 | 56 | 0.273 | 0.601 | 0.037 |
|  |  |  | Percentage | 42.9% | 57.1% |  |  |  |
|  |  | Lose | Frequency | 38 | 59 |  |  |  |
|  |  |  | Percentage | 39.2% | 60.8% |  |  |  |
| **High** | **OF-U** | Win | Frequency | 61 | 56 | 3.278 | 0.070 | 0.122 |
|  |  |  | Percentage | 52.1% | 47.9% |  |  |  |
|  |  | Lose | Frequency | 42 | 63 |  |  |  |
|  |  |  | Percentage | 40.0% | 60.0% |  |  |  |
| **Low** | **OF-S** | Win | Frequency | 1 | 0 |  | 1.000 (**) | 0.333 |
|  |  |  | Percentage | 100.0% | 0.0% |  |  |  |
|  |  | Lose | Frequency | 2 | 1 |  |  |  |
|  |  |  | Percentage | 66.7% | 33.3% |  |  |  |
| **Middle** | **OF-S** | Win | Frequency | 9 | 2 |  | 0.584 (**) | 0.171 |
|  |  |  | Percentage | 81.8% | 18.2% |  |  |  |
|  |  | Lose | Frequency | 4 | 2 |  |  |  |
|  |  |  | Percentage | 66.7% | 33.3% |  |  |  |
| **High** | **OF-S** | Win | Frequency | 3 | 3 |  | 0.600 (**) | 0.227 |
|  |  |  | Percentage | 50.0% | 50.0% |  |  |  |
|  |  | Lose | Frequency | 3 | 8 |  |  |  |
|  |  |  | Percentage | 27.3% | 72.7% |  |  |  |

Low, low-point classification; Middle, middle-point classification; High, high-point classification; ON-U, the plays where the user shot using the on-the-ball screen; ON-S, plays where the screener of the on-the-ball screen shot after receiving a pass from the user; ON-A, plays where another player shot after receiving a pass from the user of the on-the-ball screen; ON-E, plays that led to a shot through two or more extra passes after the user used the on-the-ball screen; OF-U, plays where the user shot using the off-the-ball screen; OF-S, plays where the screener of the off-the-ball screen shot.

**p* < 0.05.

**We adopted *p*-value by Fisher's method.

TABLE S46 Differences in shot-success rate of the user in movement of on-the-ball screen plays.

| **User** | **Movement of on-the-ball screen plays** |  |  | **Success** | **Fail** | **χ^2^(df=1)** | ***p*** | ***φ*** |
| --- | --- | --- | --- | --- | --- | --- | --- | --- |
| **Low** | **Around** | Win | Frequency | 1 | 1 |  | 1.000 (**) | 0.577 |
|  |  |  | Percentage | 50.0% | 50.0% |  |  |  |
|  |  | Lose | Frequency | 0 | 2 |  |  |  |
|  |  |  | Percentage | 0.0% | 100.0% |  |  |  |
| **Middle** | **Around** | Win | Frequency | 4 | 4 |  | 1.000 (**) | 0.032 |
|  |  |  | Percentage | 50.0% | 50.0% |  |  |  |
|  |  | Lose | Frequency | 7 | 8 |  |  |  |
|  |  |  | Percentage | 46.7% | 53.3% |  |  |  |
| **High** | **Around** | Win | Frequency | 3 | 6 |  | 0.523 (**) | -0.293 |
|  |  |  | Percentage | 33.3% | 66.7% |  |  |  |
|  |  | Lose | Frequency | 2 | 1 |  |  |  |
|  |  |  | Percentage | 66.7% | 33.3% |  |  |  |
| **Low** | **Center-line** | Win | Frequency | 0 | 0 |  |  |  |
|  |  |  | Percentage | 0.0% | 0.0% |  |  |  |
|  |  | Lose | Frequency | 3 | 1 |  |  |  |
|  |  |  | Percentage | 75.0% | 25.0% |  |  |  |
| **Middle** | **Center-line** | Win | Frequency | 50 | 82 | 0.463 | 0.496 | 0.042 |
|  |  |  | Percentage | 37.9% | 62.1% |  |  |  |
|  |  | Lose | Frequency | 44 | 86 |  |  |  |
|  |  |  | Percentage | 33.8% | 66.2% |  |  |  |
| **High** | **Center-line** | Win | Frequency | 80 | 87 | 2.910 | 0.088 | 0.096 |
|  |  |  | Percentage | 47.9% | 52.1% |  |  |  |
|  |  | Lose | Frequency | 58 | 93 |  |  |  |
|  |  |  | Percentage | 38.4% | 61.6% |  |  |  |
| **Low** | **End-line** | Win | Frequency | 0 | 3 |  |  |  |
|  |  |  | Percentage | 0.0% | 100.0% |  |  |  |
|  |  | Lose | Frequency | 0 | 5 |  |  |  |
|  |  |  | Percentage | 0.0% | 100.0% |  |  |  |
| **Middle** | **End-line** | Win | Frequency | 66 | 87 | .416 | .519 | .038 |
|  |  |  | Percentage | 43.1% | 56.9% |  |  |  |
|  |  | Lose | Frequency | 56 | 86 |  |  |  |
|  |  |  | Percentage | 39.4% | 60.6% |  |  |  |
| **High** | **End-line** | Win | Frequency | 82 | 109 | 0.169 | 0.681 | 0.022 |
|  |  |  | Percentage | 42.9% | 57.1% |  |  |  |
|  |  | Lose | Frequency | 71 | 103 |  |  |  |
|  |  |  | Percentage | 40.8% | 59.2% |  |  |  |
| **Low** | **ON-Down** | Win | Frequency | 0 | 2 |  |  |  |
|  |  |  | Percentage | 0.0% | 100.0% |  |  |  |
|  |  | Lose | Frequency | 0 | 1 |  |  |  |
|  |  |  | Percentage | 0.0% | 100.0% |  |  |  |
| **Middle** | **ON-Down** | Win | Frequency | 2 | 5 |  | 0.608 (**) | -0.218 |
|  |  |  | Percentage | 28.6% | 71.4% |  |  |  |
|  |  | Lose | Frequency | 4 | 4 |  |  |  |
|  |  |  | Percentage | 50.0% | 50.0% |  |  |  |
| **High** | **ON-Down** | Win | Frequency | 3 | 14 |  | 0.365 (**) | -0.246 |
|  |  |  | Percentage | 17.6% | 82.4% |  |  |  |
|  |  | Lose | Frequency | 4 | 6 |  |  |  |
|  |  |  | Percentage | 40.0% | 60.0% |  |  |  |

Low, low-point classification; Middle, middle-point classification; High, high-point classification; Around, plays where the screener held a ball; Center-line, plays where the user moved toward the center-line side against the screener; End-line, plays where the user moved toward the end-line side against the screener; ON-Down, plays where the screener was on the center-line side of the defense who protected the user holding a ball in a Top or 3P on Top extension.

**We adopted *p*-value by Fisher's method.

TABLE S47 Differences in shot-success rate of the user in movement of off-the-ball screen plays.

| **User** | **Movement of off-the-ball screen plays** |  |  | **Success** | **Fail** | **χ^2^(df=1)** | ***p*** | ***φ*** |
| --- | --- | --- | --- | --- | --- | --- | --- | --- |
| **Low** | **Back** | Win | Frequency | 1 | 0 |  |  |  |
|  |  |  | Percentage | 100.0% | 0.0% |  |  |  |
|  |  | Lose | Frequency | 2 | 0 |  |  |  |
|  |  |  | Percentage | 100.0% | 0.0% |  |  |  |
| **Middle** | **Back** | Win | Frequency | 14 | 11 | 1.481 | 0.224 | 0.167 |
|  |  |  | Percentage | 56.0% | 44.0% |  |  |  |
|  |  | Lose | Frequency | 11 | 17 |  |  |  |
|  |  |  | Percentage | 39.3% | 60.7% |  |  |  |
| **High** | **Back** | Win | Frequency | 14 | 12 | 2.932 | 0.087 | 0.231 |
|  |  |  | Percentage | 53.8% | 46.2% |  |  |  |
|  |  | Lose | Frequency | 9 | 20 |  |  |  |
|  |  |  | Percentage | 31.0% | 69.0% |  |  |  |
| **Low** | **Cross** | Win | Frequency | 1 | 0 |  |  |  |
|  |  |  | Percentage | 100.0% | 0.0% |  |  |  |
|  |  | Lose | Frequency | 1 | 0 |  |  |  |
|  |  |  | Percentage | 100.0% | 0.0% |  |  |  |
| **Middle** | **Cross** | Win | Frequency | 5 | 3 |  | 1.000 (**) | -0.135 |
|  |  |  | Percentage | 62.5% | 37.5% |  |  |  |
|  |  | Lose | Frequency | 6 | 2 |  |  |  |
|  |  |  | Percentage | 75.0% | 25.0% |  |  |  |
| **High** | **Cross** | Win | Frequency | 11 | 5 | 1.094 | 0.296 | 0.191 |
|  |  |  | Percentage | 68.8% | 31.3% |  |  |  |
|  |  | Lose | Frequency | 7 | 7 |  |  |  |
|  |  |  | Percentage | 50.0% | 50.0% |  |  |  |
| **Low** | **Down** | Win | Frequency | 0 | 1 |  |  |  |
|  |  |  | Percentage | 0.0% | 100.0% |  |  |  |
|  |  | Lose | Frequency | 0 | 0 |  |  |  |
|  |  |  | Percentage | 0.0% | 0.0% |  |  |  |
| **Middle** | **Down** | Win | Frequency | 12 | 11 | 4.057* | 0.044 | 0.291 |
|  |  |  | Percentage | 52.2% | 47.8% |  |  |  |
|  |  | Lose | Frequency | 6 | 19 |  |  |  |
|  |  |  | Percentage | 24.0% | 76.0% |  |  |  |
| **High** | **Down** | Win | Frequency | 15 | 15 | 1.984 | 0.159 | 0.180 |
|  |  |  | Percentage | 50.0% | 50.0% |  |  |  |
|  |  | Lose | Frequency | 10 | 21 |  |  |  |
|  |  |  | Percentage | 32.3% | 67.7% |  |  |  |
| **Low** | **Flare** | Win | Frequency | 4 | 5 |  | 1.000 (**) | -0.056 |
|  |  |  | Percentage | 44.4% | 55.6% |  |  |  |
|  |  | Lose | Frequency | 5 | 5 |  |  |  |
|  |  |  | Percentage | 50.0% | 50.0% |  |  |  |
| **Middle** | **Flare** | Win | Frequency | 19 | 33 | 0.730 | 0.393 | -0.088 |
|  |  |  | Percentage | 36.5% | 63.5% |  |  |  |
|  |  | Lose | Frequency | 19 | 23 |  |  |  |
|  |  |  | Percentage | 45.2% | 54.8% |  |  |  |
| **High** | **Flare** | Win | Frequency | 24 | 27 | 0.031 | 0.861 | 0.018 |
|  |  |  | Percentage | 47.1% | 52.9% |  |  |  |
|  |  | Lose | Frequency | 19 | 23 |  |  |  |
|  |  |  | Percentage | 45.2% | 54.8% |  |  |  |

Low, low-point classification; Middle, middle-point classification; High, high-point classification; Back, plays where the screener was on the end-line side of the defense who protected the user; Cross, plays where the screener was on the middle-line (the imaginary line connecting baskets running through the center of the court) side of the defense who protected the user; Down, plays where the screener on the center-line side of the defense who protected the user; Flare, plays where the screener on the side-line side of the defense who protected the user.

**p* < 0.05.

**We adopted *p*-value by Fisher's method.

TABLE S48 Differences in shot-success rate of the user × the screener.

| **User** | **Screener** |  |  | **Success** | **Fail** | **χ^2^(df=1)** | ***p*** | ***φ*** |
| --- | --- | --- | --- | --- | --- | --- | --- | --- |
| **Low** | **Low** | Win | Frequency | 0 | 0 |  |  |  |
|  |  |  | Percentage | 0.0% | 0.0% |  |  |  |
|  |  | Lose | Frequency | 1 | 1 |  |  |  |
|  |  |  | Percentage | 50.0% | 50.0% |  |  |  |
| **Low** | **Middle** | Win | Frequency | 4 | 6 |  | 0.695 (**) | -0.125 |
|  |  |  | Percentage | 40.0% | 60.0% |  |  |  |
|  |  | Lose | Frequency | 9 | 8 |  |  |  |
|  |  |  | Percentage | 52.9% | 47.1% |  |  |  |
| **Low** | **High** | Win | Frequency | 2 | 5 |  | 1.000 (**) | 0.141 |
|  |  |  | Percentage | 28.6% | 71.4% |  |  |  |
|  |  | Lose | Frequency | 1 | 5 |  |  |  |
|  |  |  | Percentage | 16.7% | 83.3% |  |  |  |
| **Middle** | **Low** | Win | Frequency | 43 | 36 | 3.410 | 0.065 | 0.138 |
|  |  |  | Percentage | 54.4% | 45.6% |  |  |  |
|  |  | Lose | Frequency | 41 | 60 |  |  |  |
|  |  |  | Percentage | 40.6% | 59.4% |  |  |  |
| **Middle** | **Middle** | Win | Frequency | 68 | 119 | 0.121 | 0.728 | -0.018 |
|  |  |  | Percentage | 36.4% | 63.6% |  |  |  |
|  |  | Lose | Frequency | 72 | 117 |  |  |  |
|  |  |  | Percentage | 38.1% | 61.9% |  |  |  |
| **Middle** | **High** | Win | Frequency | 61 | 77 | 1.361 | 0.243 | 0.075 |
|  |  |  | Percentage | 44.2% | 55.8% |  |  |  |
|  |  | Lose | Frequency | 39 | 67 |  |  |  |
|  |  |  | Percentage | 36.8% | 63.2% |  |  |  |
| **High** | **Low** | Win | Frequency | 70 | 88 | 0.107 | 0.744 | 0.018 |
|  |  |  | Percentage | 44.3% | 55.7% |  |  |  |
|  |  | Lose | Frequency | 77 | 104 |  |  |  |
|  |  |  | Percentage | 42.5% | 57.5% |  |  |  |
| **High** | **Middle** | Win | Frequency | 123 | 137 | 3.239 | 0.072 | 0.084 |
|  |  |  | Percentage | 47.3% | 52.7% |  |  |  |
|  |  | Lose | Frequency | 77 | 121 |  |  |  |
|  |  |  | Percentage | 38.9% | 61.1% |  |  |  |
| **High** | **High** | Win | Frequency | 37 | 43 | 1.894 | 0.169 | 0.112 |
|  |  |  | Percentage | 46.3% | 53.8% |  |  |  |
|  |  | Lose | Frequency | 25 | 46 |  |  |  |
|  |  |  | Percentage | 35.2% | 64.8% |  |  |  |

Low, low-point classification; Middle, middle-point classification; High, high-point classification.

**We adopted *p*-value by Fisher's method.

TABLE S49 Differences in shot-success rate of the user × the passer.

| **User** | **Passer** |  |  | **Success** | **Fail** | **χ^2^(df=1)** | ***p*** | ***φ*** |
| --- | --- | --- | --- | --- | --- | --- | --- | --- |
| **Low** | **Low** | Win | Frequency | 0 | 1 |  | 1.000 (**) | -0.250 |
|  |  |  | Percentage | 0.0% | 100.0% |  |  |  |
|  |  | Lose | Frequency | 1 | 3 |  |  |  |
|  |  |  | Percentage | 25.0% | 75.0% |  |  |  |
| **Low** | **Middle** | Win | Frequency | 2 | 4 |  | 0.567 (**) | -0.333 |
|  |  |  | Percentage | 33.3% | 66.7% |  |  |  |
|  |  | Lose | Frequency | 4 | 2 |  |  |  |
|  |  |  | Percentage | 66.7% | 33.3% |  |  |  |
| **Low** | **High** | Win | Frequency | 4 | 2 |  | 0.633 (**) | 0.163 |
|  |  |  | Percentage | 66.7% | 33.3% |  |  |  |
|  |  | Lose | Frequency | 5 | 5 |  |  |  |
|  |  |  | Percentage | 50.0% | 50.0% |  |  |  |
| **Middle** | **Low** | Win | Frequency | 2 | 5 |  | 0.358 (**) | -0.270 |
|  |  |  | Percentage | 28.6% | 71.4% |  |  |  |
|  |  | Lose | Frequency | 5 | 4 |  |  |  |
|  |  |  | Percentage | 55.6% | 44.4% |  |  |  |
| **Middle** | **Middle** | Win | Frequency | 72 | 83 | 0.255 | 0.614 | 0.028 |
|  |  |  | Percentage | 46.5% | 53.5% |  |  |  |
|  |  | Lose | Frequency | 76 | 98 |  |  |  |
|  |  |  | Percentage | 43.7% | 56.3% |  |  |  |
| **Middle** | **High** | Win | Frequency | 37 | 49 | 0.458 | 0.499 | 0.051 |
|  |  |  | Percentage | 43.0% | 57.0% |  |  |  |
|  |  | Lose | Frequency | 35 | 57 |  |  |  |
|  |  |  | Percentage | 38.0% | 62.0% |  |  |  |
| **High** | **Low** | Win | Frequency | 6 | 8 | 0.013 | 0.908 | 0.019 |
|  |  |  | Percentage | 42.9% | 57.1% |  |  |  |
|  |  | Lose | Frequency | 9 | 13 |  |  |  |
|  |  |  | Percentage | 40.9% | 59.1% |  |  |  |
| **High** | **Middle** | Win | Frequency | 47 | 56 | 0.074 | 0.786 | -0.021 |
|  |  |  | Percentage | 45.6% | 54.4% |  |  |  |
|  |  | Lose | Frequency | 32 | 35 |  |  |  |
|  |  |  | Percentage | 47.8% | 52.2% |  |  |  |
| **High** | **High** | Win | Frequency | 92 | 112 | 0.341 | 0.559 | 0.029 |
|  |  |  | Percentage | 45.1% | 54.9% |  |  |  |
|  |  | Lose | Frequency | 81 | 111 |  |  |  |
|  |  |  | Percentage | 42.2% | 57.8% |  |  |  |

Low, low-point classification; Middle, middle-point classification; High, high-point classification.

**We adopted *p*-value by Fisher's method.

TABLE S50 Differences in shot-success rate depending on the PC of the screener.

| **Screener** |  |  | **Success** | **Fail** | **χ^2^(df=1)** | ***p*** | ***φ*** |
| --- | --- | --- | --- | --- | --- | --- | --- |
| **Low** | **Win** | Frequency | 113 | 124 | 1.746 | 0.186 | 0.058 |
|  |  | Percentage | 47.7% | 52.3% |  |  |  |
|  | **Lose** | Frequency | 119 | 165 |  |  |  |
|  |  | Percentage | 41.9% | 58.1% |  |  |  |
| **Middle** | **Win** | Frequency | 195 | 262 | 1.124 | 0.289 | 0.036 |
|  |  | Percentage | 42.7% | 57.3% |  |  |  |
|  | **Lose** | Frequency | 158 | 246 |  |  |  |
|  |  | Percentage | 39.1% | 60.9% |  |  |  |
| **High** | **Win** | Frequency | 100 | 125 | 3.338 | 0.068 | 0.090 |
|  |  | Percentage | 44.4% | 55.6% |  |  |  |
|  | **Lose** | Frequency | 65 | 118 |  |  |  |
|  |  | Percentage | 35.5% | 64.5% |  |  |  |

PC, player classification; Low, low-point classification; Middle, middle-point classification; High, high-point classification.

TABLE S51 Differences in shot-success rate of the screener in shot location.

| **Screener** | **Shot Location** |  |  | **Success** | **Fail** | **χ^2^(df=1)** | ***p*** | ***φ*** |
| --- | --- | --- | --- | --- | --- | --- | --- | --- |
| **Low** | **PL** | Win | Frequency | 45 | 37 | 0.427 | 0.514 | -0.050 |
|  |  |  | Percentage | 54.9% | 45.1% |  |  |  |
|  |  | Lose | Frequency | 55 | 37 |  |  |  |
|  |  |  | Percentage | 59.8% | 40.2% |  |  |  |
| **Middle** | **PL** | Win | Frequency | 67 | 48 | 0.282 | 0.595 | 0.036 |
|  |  |  | Percentage | 58.3% | 41.7% |  |  |  |
|  |  | Lose | Frequency | 58 | 48 |  |  |  |
|  |  |  | Percentage | 54.7% | 45.3% |  |  |  |
| **High** | **PL** | Win | Frequency | 30 | 22 | 0.388 | 0.533 | 0.065 |
|  |  |  | Percentage | 57.7% | 42.3% |  |  |  |
|  |  | Lose | Frequency | 21 | 20 |  |  |  |
|  |  |  | Percentage | 51.2% | 48.8% |  |  |  |
| **Low** | **PH** | Win | Frequency | 11 | 9 | 9.586* | 0.002 | 0.462 |
|  |  |  | Percentage | 55.0% | 45.0% |  |  |  |
|  |  | Lose | Frequency | 3 | 22 |  |  |  |
|  |  |  | Percentage | 12.0% | 88.0% |  |  |  |
| **Middle** | **PH** | Win | Frequency | 14 | 24 | 0.019 | 0.891 | 0.016 |
|  |  |  | Percentage | 36.8% | 63.2% |  |  |  |
|  |  | Lose | Frequency | 12 | 22 |  |  |  |
|  |  |  | Percentage | 35.3% | 64.7% |  |  |  |
| **High** | **PH** | Win | Frequency | 12 | 4 | 7.200* | 0.007 | 0.447 |
|  |  |  | Percentage | 75.0% | 25.0% |  |  |  |
|  |  | Lose | Frequency | 6 | 14 |  |  |  |
|  |  |  | Percentage | 30.0% | 70.0% |  |  |  |
| **Low** | **Top** | Win | Frequency | 4 | 10 | 1.106 | 0.293 | -0.189 |
|  |  |  | Percentage | 28.6% | 71.4% |  |  |  |
|  |  | Lose | Frequency | 8 | 9 |  |  |  |
|  |  |  | Percentage | 47.1% | 52.9% |  |  |  |
| **Middle** | **Top** | Win | Frequency | 22 | 30 | 0.096 | 0.757 | -0.032 |
|  |  |  | Percentage | 42.3% | 57.7% |  |  |  |
|  |  | Lose | Frequency | 20 | 24 |  |  |  |
|  |  |  | Percentage | 45.5% | 54.5% |  |  |  |
| **High** | **Top** | Win | Frequency | 9 | 16 | 0.287 | 0.592 | 0.079 |
|  |  |  | Percentage | 36.0% | 64.0% |  |  |  |
|  |  | Lose | Frequency | 6 | 15 |  |  |  |
|  |  |  | Percentage | 28.6% | 71.4% |  |  |  |
| **Low** | **Corner** | Win | Frequency | 29 | 32 | 0.012 | 0.911 | 0.010 |
|  |  |  | Percentage | 47.5% | 52.5% |  |  |  |
|  |  | Lose | Frequency | 34 | 39 |  |  |  |
|  |  |  | Percentage | 46.6% | 53.4% |  |  |  |
| **Middle** | **Corner** | Win | Frequency | 31 | 56 | 2.206 | 0.137 | -0.115 |
|  |  |  | Percentage | 35.6% | 64.4% |  |  |  |
|  |  | Lose | Frequency | 38 | 43 |  |  |  |
|  |  |  | Percentage | 46.9% | 53.1% |  |  |  |
| **High** | **Corner** | Win | Frequency | 28 | 22 | 4.322* | 0.038 | 0.224 |
|  |  |  | Percentage | 56.0% | 44.0% |  |  |  |
|  |  | Lose | Frequency | 12 | 24 |  |  |  |
|  |  |  | Percentage | 33.3% | 66.7% |  |  |  |
| **Low** | **Wing** | Win | Frequency | 19 | 24 | 1.792 | 0.181 | 0.144 |
|  |  |  | Percentage | 44.2% | 55.8% |  |  |  |
|  |  | Lose | Frequency | 13 | 30 |  |  |  |
|  |  |  | Percentage | 30.2% | 69.8% |  |  |  |
| **Middle** | **Wing** | Win | Frequency | 35 | 63 | 1.518 | 0.218 | 0.095 |
|  |  |  | Percentage | 35.7% | 64.3% |  |  |  |
|  |  | Lose | Frequency | 19 | 52 |  |  |  |
|  |  |  | Percentage | 26.8% | 73.2% |  |  |  |
| **High** | **Wing** | Win | Frequency | 15 | 39 | 0.415 | 0.520 | -0.069 |
|  |  |  | Percentage | 27.8% | 72.2% |  |  |  |
|  |  | Lose | Frequency | 11 | 21 |  |  |  |
|  |  |  | Percentage | 34.4% | 65.6% |  |  |  |
| **Low** | **3P** | Win | Frequency | 5 | 12 |  | 0.472 (**) | 0.135 |
|  |  |  | Percentage | 29.4% | 70.6% |  |  |  |
|  |  | Lose | Frequency | 6 | 28 |  |  |  |
|  |  |  | Percentage | 17.6% | 82.4% |  |  |  |
| **Middle** | **3P** | Win | Frequency | 26 | 41 | 8.686* | 0.003 | 0.254 |
|  |  |  | Percentage | 38.8% | 61.2% |  |  |  |
|  |  | Lose | Frequency | 11 | 57 |  |  |  |
|  |  |  | Percentage | 16.2% | 83.8% |  |  |  |
| **High** | **3P** | Win | Frequency | 6 | 22 | 0.279 | 0.597 | -0.068 |
|  |  |  | Percentage | 21.4% | 78.6% |  |  |  |
|  |  | Lose | Frequency | 9 | 24 |  |  |  |
|  |  |  | Percentage | 27.3% | 72.7% |  |  |  |

Low, low-point classification; Middle, middle-point classification; High, high-point classification; PL, paint-low; PH, paint-high; 3P, 3-point field goal area.

**p* < 0.05.

**We adopted *p*-value by Fisher's method.

TABLE S52 Differences in shot-success rate of the screener in screen location.

| **Screener** | **Screen Location** |  |  | **Success** | **Fail** | **χ^2^(df=1)** | ***p*** | ***φ*** |
| --- | --- | --- | --- | --- | --- | --- | --- | --- |
| **Low** | **PL** | Win | Frequency | 25 | 11 | 2.010 | 0.156 | 0.169 |
|  |  |  | Percentage | 69.4% | 30.6% |  |  |  |
|  |  | Lose | Frequency | 18 | 16 |  |  |  |
|  |  |  | Percentage | 52.9% | 47.1% |  |  |  |
| **Middle** | **PL** | Win | Frequency | 7 | 5 | 0.083 | 0.774 | 0.053 |
|  |  |  | Percentage | 58.3% | 41.7% |  |  |  |
|  |  | Lose | Frequency | 9 | 8 |  |  |  |
|  |  |  | Percentage | 52.9% | 47.1% |  |  |  |
| **High** | **PL** | Win | Frequency | 3 | 2 |  | 1.000 (**) | 0.100 |
|  |  |  | Percentage | 60.0% | 40.0% |  |  |  |
|  |  | Lose | Frequency | 2 | 2 |  |  |  |
|  |  |  | Percentage | 50.0% | 50.0% |  |  |  |
| **Low** | **PH** | Win | Frequency | 21 | 22 | 0.011 | 0.915 | 0.014 |
|  |  |  | Percentage | 48.8% | 51.2% |  |  |  |
|  |  | Lose | Frequency | 9 | 10 |  |  |  |
|  |  |  | Percentage | 47.4% | 52.6% |  |  |  |
| **Middle** | **PH** | Win | Frequency | 10 | 15 | 0.004 | 0.951 | 0.009 |
|  |  |  | Percentage | 40.0% | 60.0% |  |  |  |
|  |  | Lose | Frequency | 9 | 14 |  |  |  |
|  |  |  | Percentage | 39.1% | 60.9% |  |  |  |
| **High** | **PH** | Win | Frequency | 7 | 14 | 0.069 | 0.793 | -0.043 |
|  |  |  | Percentage | 33.3% | 66.7% |  |  |  |
|  |  | Lose | Frequency | 6 | 10 |  |  |  |
|  |  |  | Percentage | 37.5% | 62.5% |  |  |  |
| **Low** | **Top** | Win | Frequency | 5 | 5 |  | 0.263 (**) | 0.199 |
|  |  |  | Percentage | 50.0% | 50.0% |  |  |  |
|  |  | Lose | Frequency | 8 | 20 |  |  |  |
|  |  |  | Percentage | 28.6% | 71.4% |  |  |  |
| **Middle** | **Top** | Win | Frequency | 15 | 22 | 0.285 | 0.593 | 0.055 |
|  |  |  | Percentage | 40.5% | 59.5% |  |  |  |
|  |  | Lose | Frequency | 20 | 37 |  |  |  |
|  |  |  | Percentage | 35.1% | 64.9% |  |  |  |
| **High** | **Top** | Win | Frequency | 8 | 15 | 0.040 | 0.842 | 0.028 |
|  |  |  | Percentage | 34.8% | 65.2% |  |  |  |
|  |  | Lose | Frequency | 9 | 19 |  |  |  |
|  |  |  | Percentage | 32.1% | 67.9% |  |  |  |
| **Low** | **Corner** | Win | Frequency | 41 | 44 | 0.094 | 0.759 | 0.024 |
|  |  |  | Percentage | 48.2% | 51.8% |  |  |  |
|  |  | Lose | Frequency | 39 | 46 |  |  |  |
|  |  |  | Percentage | 45.9% | 54.1% |  |  |  |
| **Middle** | **Corner** | Win | Frequency | 60 | 64 | 1.978 | 0.160 | 0.094 |
|  |  |  | Percentage | 48.4% | 51.6% |  |  |  |
|  |  | Lose | Frequency | 39 | 61 |  |  |  |
|  |  |  | Percentage | 39.0% | 61.0% |  |  |  |
| **High** | **Corner** | Win | Frequency | 24 | 27 | 0.078 | 0.781 | 0.029 |
|  |  |  | Percentage | 47.1% | 52.9% |  |  |  |
|  |  | Lose | Frequency | 19 | 24 |  |  |  |
|  |  |  | Percentage | 44.2% | 55.8% |  |  |  |
| **Low** | **Wing** | Win | Frequency | 19 | 37 | 0.139 | 0.710 | -0.030 |
|  |  |  | Percentage | 33.9% | 66.1% |  |  |  |
|  |  | Lose | Frequency | 38 | 65 |  |  |  |
|  |  |  | Percentage | 36.9% | 63.1% |  |  |  |
| **Middle** | **Wing** | Win | Frequency | 84 | 141 | 0.043 | 0.835 | 0.010 |
|  |  |  | Percentage | 37.3% | 62.7% |  |  |  |
|  |  | Lose | Frequency | 61 | 107 |  |  |  |
|  |  |  | Percentage | 36.3% | 63.7% |  |  |  |
| **High** | **Wing** | Win | Frequency | 52 | 62 | 3.547 | 0.060 | 0.136 |
|  |  |  | Percentage | 45.6% | 54.4% |  |  |  |
|  |  | Lose | Frequency | 25 | 53 |  |  |  |
|  |  |  | Percentage | 32.1% | 67.9% |  |  |  |
| **Low** | **3P** | Win | Frequency | 2 | 5 |  | 0.648 (**) | -0.171 |
|  |  |  | Percentage | 28.6% | 71.4% |  |  |  |
|  |  | Lose | Frequency | 7 | 8 |  |  |  |
|  |  |  | Percentage | 46.7% | 53.3% |  |  |  |
| **Middle** | **3P** | Win | Frequency | 19 | 15 | 0.154 | 0.694 | 0.046 |
|  |  |  | Percentage | 55.9% | 44.1% |  |  |  |
|  |  | Lose | Frequency | 20 | 19 |  |  |  |
|  |  |  | Percentage | 51.3% | 48.7% |  |  |  |
| **High** | **3P** | Win | Frequency | 6 | 5 |  | 0.241 (**) | 0.263 |
|  |  |  | Percentage | 54.5% | 45.5% |  |  |  |
|  |  | Lose | Frequency | 4 | 10 |  |  |  |
|  |  |  | Percentage | 28.6% | 71.4% |  |  |  |

Low, low-point classification; Middle, middle-point classification; High, high-point classification; PL, paint-low; PH, paint-high; 3P, 3-point field goal area.

**We adopted *p*-value by Fisher's method.

TABLE S53 Differences in shot-success rate of the screener in pass location.

| **Screener** | **Pass Location** |  |  | **Success** | **Fail** | **χ^2^(df=1)** | ***p*** | ***φ*** |
| --- | --- | --- | --- | --- | --- | --- | --- | --- |
| **Low** | **PL** | Win | Frequency | 3 | 4 |  | 1.000 (**) | -0.169 |
|  |  |  | Percentage | 42.9% | 57.1% |  |  |  |
|  |  | Lose | Frequency | 3 | 2 |  |  |  |
|  |  |  | Percentage | 60.0% | 40.0% |  |  |  |
| **Middle** | **PL** | Win | Frequency | 6 | 5 |  | 1.000 (**) | -0.051 |
|  |  |  | Percentage | 54.5% | 45.5% |  |  |  |
|  |  | Lose | Frequency | 3 | 2 |  |  |  |
|  |  |  | Percentage | 60.0% | 40.0% |  |  |  |
| **High** | **PL** | Win | Frequency | 2 | 2 |  | 0.467 (**) | 0.500 |
|  |  |  | Percentage | 50.0% | 50.0% |  |  |  |
|  |  | Lose | Frequency | 0 | 2 |  |  |  |
|  |  |  | Percentage | 0.0% | 100.0% |  |  |  |
| **Low** | **PH** | Win | Frequency | 14 | 13 | 0.017 | 0.897 | 0.018 |
|  |  |  | Percentage | 51.9% | 48.1% |  |  |  |
|  |  | Lose | Frequency | 11 | 11 |  |  |  |
|  |  |  | Percentage | 50.0% | 50.0% |  |  |  |
| **Middle** | **PH** | Win | Frequency | 21 | 21 | 2.957 | 0.086 | 0.199 |
|  |  |  | Percentage | 50.0% | 50.0% |  |  |  |
|  |  | Lose | Frequency | 10 | 23 |  |  |  |
|  |  |  | Percentage | 30.3% | 69.7% |  |  |  |
| **High** | **PH** | Win | Frequency | 6 | 10 |  | 1.000 (**) | -0.010 |
|  |  |  | Percentage | 37.5% | 62.5% |  |  |  |
|  |  | Lose | Frequency | 5 | 8 |  |  |  |
|  |  |  | Percentage | 38.5% | 61.5% |  |  |  |
| **Low** | **Top** | Win | Frequency | 14 | 12 | 0.161 | 0.688 | 0.047 |
|  |  |  | Percentage | 53.8% | 46.2% |  |  |  |
|  |  | Lose | Frequency | 23 | 24 |  |  |  |
|  |  |  | Percentage | 48.9% | 51.1% |  |  |  |
| **Middle** | **Top** | Win | Frequency | 13 | 29 | 5.365* | 0.021 | -0.238 |
|  |  |  | Percentage | 31.0% | 69.0% |  |  |  |
|  |  | Lose | Frequency | 29 | 24 |  |  |  |
|  |  |  | Percentage | 54.7% | 45.3% |  |  |  |
| **High** | **Top** | Win | Frequency | 12 | 15 | 0.103 | 0.748 | -0.051 |
|  |  |  | Percentage | 44.4% | 55.6% |  |  |  |
|  |  | Lose | Frequency | 6 | 6 |  |  |  |
|  |  |  | Percentage | 50.0% | 50.0% |  |  |  |
| **Low** | **Corner** | Win | Frequency | 18 | 27 | 0.049 | 0.825 | 0.025 |
|  |  |  | Percentage | 40.0% | 60.0% |  |  |  |
|  |  | Lose | Frequency | 12 | 20 |  |  |  |
|  |  |  | Percentage | 37.5% | 62.5% |  |  |  |
| **Middle** | **Corner** | Win | Frequency | 21 | 31 | 0.415 | 0.520 | -0.065 |
|  |  |  | Percentage | 40.4% | 59.6% |  |  |  |
|  |  | Lose | Frequency | 22 | 25 |  |  |  |
|  |  |  | Percentage | 46.8% | 53.2% |  |  |  |
| **High** | **Corner** | Win | Frequency | 16 | 17 | 0.006 | 0.938 | 0.011 |
|  |  |  | Percentage | 48.5% | 51.5% |  |  |  |
|  |  | Lose | Frequency | 9 | 10 |  |  |  |
|  |  |  | Percentage | 47.4% | 52.6% |  |  |  |
| **Low** | **Wing** | Win | Frequency | 21 | 26 | 0.141 | 0.707 | -0.036 |
|  |  |  | Percentage | 44.7% | 55.3% |  |  |  |
|  |  | Lose | Frequency | 29 | 31 |  |  |  |
|  |  |  | Percentage | 48.3% | 51.7% |  |  |  |
| **Middle** | **Wing** | Win | Frequency | 24 | 44 | 1.220 | 0.269 | -0.093 |
|  |  |  | Percentage | 35.3% | 64.7% |  |  |  |
|  |  | Lose | Frequency | 32 | 40 |  |  |  |
|  |  |  | Percentage | 44.4% | 55.6% |  |  |  |
| **High** | **Wing** | Win | Frequency | 22 | 19 | 1.104 | 0.293 | 0.120 |
|  |  |  | Percentage | 53.7% | 46.3% |  |  |  |
|  |  | Lose | Frequency | 15 | 21 |  |  |  |
|  |  |  | Percentage | 41.7% | 58.3% |  |  |  |
| **Low** | **3P** | Win | Frequency | 11 | 13 | 0.118 | 0.731 | 0.043 |
|  |  |  | Percentage | 45.8% | 54.2% |  |  |  |
|  |  | Lose | Frequency | 17 | 24 |  |  |  |
|  |  |  | Percentage | 41.5% | 58.5% |  |  |  |
| **Middle** | **3P** | Win | Frequency | 28 | 18 | 6.712* | 0.010 | 0.269 |
|  |  |  | Percentage | 60.9% | 39.1% |  |  |  |
|  |  | Lose | Frequency | 16 | 31 |  |  |  |
|  |  |  | Percentage | 34.0% | 66.0% |  |  |  |
| **High** | **3P** | Win | Frequency | 6 | 12 |  | 0.493 (**) | 0.141 |
|  |  |  | Percentage | 33.3% | 66.7% |  |  |  |
|  |  | Lose | Frequency | 6 | 23 |  |  |  |
|  |  |  | Percentage | 20.7% | 79.3% |  |  |  |

Low, low-point classification; Middle, middle-point classification; High, high-point classification; PL, paint-low; PH, paint-high; 3P, 3-point field goal area.

**p* < 0.05.

**We adopted *p*-value by Fisher's method.

TABLE S54 Differences in shot-success rate of the screener in type of screen.

| **Screener** | **Type of Screen** |  |  | **Success** | **Fail** | **χ^2^(df=1)** | ***p*** | ***φ*** |
| --- | --- | --- | --- | --- | --- | --- | --- | --- |
| **Low** | **On-the-ball screen** | Win | Frequency | 56 | 70 | 0.602 | 0.438 | 0.044 |
|  |  |  | Percentage | 44.4% | 55.6% |  |  |  |
|  |  | Lose | Frequency | 72 | 108 |  |  |  |
|  |  |  | Percentage | 40.0% | 60.0% |  |  |  |
| **Middle** | **On-the-ball screen** | Win | Frequency | 157 | 217 | 0.460 | 0.498 | 0.026 |
|  |  |  | Percentage | 42.0% | 58.0% |  |  |  |
|  |  | Lose | Frequency | 123 | 189 |  |  |  |
|  |  |  | Percentage | 39.4% | 60.6% |  |  |  |
| **High** | **On-the-ball screen** | Win | Frequency | 78 | 103 | 2.158 | 0.142 | 0.081 |
|  |  |  | Percentage | 43.1% | 56.9% |  |  |  |
|  |  | Lose | Frequency | 52 | 96 |  |  |  |
|  |  |  | Percentage | 35.1% | 64.9% |  |  |  |
| **Low** | **Off-the-ball screen** | Win | Frequency | 57 | 54 | 0.816 | 0.366 | 0.062 |
|  |  |  | Percentage | 51.4% | 48.6% |  |  |  |
|  |  | Lose | Frequency | 47 | 57 |  |  |  |
|  |  |  | Percentage | 45.2% | 54.8% |  |  |  |
| **Middle** | **Off-the-ball screen** | Win | Frequency | 38 | 45 | 1.075 | 0.300 | 0.078 |
|  |  |  | Percentage | 45.8% | 54.2% |  |  |  |
|  |  | Lose | Frequency | 35 | 57 |  |  |  |
|  |  |  | Percentage | 38.0% | 62.0% |  |  |  |
| **High** | **Off-the-ball screen** | Win | Frequency | 22 | 22 | 1.306 | 0.253 | 0.129 |
|  |  |  | Percentage | 50.0% | 50.0% |  |  |  |
|  |  | Lose | Frequency | 13 | 22 |  |  |  |
|  |  |  | Percentage | 37.1% | 62.9% |  |  |  |

Low, low-point classification; Middle, middle-point classification; High, high-point classification.

TABLE S55 Differences in shot-success rate of the screener in type of screen-play.

| **Screener** | **Type of Screen-play** |  |  | **Success** | **Fail** | **χ^2^(df=1)** | ***p*** | ***φ*** |
| --- | --- | --- | --- | --- | --- | --- | --- | --- |
| **Low** | **ON-U** | Win | Frequency | 32 | 29 | 6.398* | 0.011 | 0.215 |
|  |  |  | Percentage | 52.5% | 47.5% |  |  |  |
|  |  | Lose | Frequency | 24 | 53 |  |  |  |
|  |  |  | Percentage | 31.2% | 68.8% |  |  |  |
| **Middle** | **ON-U** | Win | Frequency | 83 | 119 | 3.350 | 0.067 | 0.098 |
|  |  |  | Percentage | 41.1% | 58.9% |  |  |  |
|  |  | Lose | Frequency | 47 | 102 |  |  |  |
|  |  |  | Percentage | 31.5% | 68.5% |  |  |  |
| **High** | **ON-U** | Win | Frequency | 38 | 48 | 1.220 | 0.269 | 0.085 |
|  |  |  | Percentage | 44.2% | 55.8% |  |  |  |
|  |  | Lose | Frequency | 29 | 52 |  |  |  |
|  |  |  | Percentage | 35.8% | 64.2% |  |  |  |
| **Low** | **ON-S** | Win | Frequency | 7 | 7 | 1.146 | 0.284 | -0.186 |
|  |  |  | Percentage | 50.0% | 50.0% |  |  |  |
|  |  | Lose | Frequency | 13 | 6 |  |  |  |
|  |  |  | Percentage | 68.4% | 31.6% |  |  |  |
| **Middle** | **ON-S** | Win | Frequency | 12 | 18 | 2.200 | 0.138 | -0.183 |
|  |  |  | Percentage | 40.0% | 60.0% |  |  |  |
|  |  | Lose | Frequency | 21 | 15 |  |  |  |
|  |  |  | Percentage | 58.3% | 41.7% |  |  |  |
| **High** | **ON-S** | Win | Frequency | 12 | 13 | 0.242 | 0.622 | 0.078 |
|  |  |  | Percentage | 48.0% | 52.0% |  |  |  |
|  |  | Lose | Frequency | 6 | 9 |  |  |  |
|  |  |  | Percentage | 40.0% | 60.0% |  |  |  |
| **Low** | **ON-A** | Win | Frequency | 13 | 26 | 0.434 | 0.510 | -0.068 |
|  |  |  | Percentage | 33.3% | 66.7% |  |  |  |
|  |  | Lose | Frequency | 22 | 33 |  |  |  |
|  |  |  | Percentage | 40.0% | 60.0% |  |  |  |
| **Middle** | **ON-A** | Win | Frequency | 44 | 53 | 1.247 | 0.264 | 0.083 |
|  |  |  | Percentage | 45.4% | 54.6% |  |  |  |
|  |  | Lose | Frequency | 32 | 54 |  |  |  |
|  |  |  | Percentage | 37.2% | 62.8% |  |  |  |
| **High** | **ON-A** | Win | Frequency | 17 | 25 | 0.533 | 0.465 | 0.084 |
|  |  |  | Percentage | 40.5% | 59.5% |  |  |  |
|  |  | Lose | Frequency | 11 | 23 |  |  |  |
|  |  |  | Percentage | 32.4% | 67.6% |  |  |  |
| **Low** | **ON-E** | Win | Frequency | 4 | 8 |  | 0.729 (**) | -0.106 |
|  |  |  | Percentage | 33.3% | 66.7% |  |  |  |
|  |  | Lose | Frequency | 13 | 16 |  |  |  |
|  |  |  | Percentage | 44.8% | 55.2% |  |  |  |
| **Middle** | **ON-E** | Win | Frequency | 18 | 27 | 2.229 | 0.135 | -0.161 |
|  |  |  | Percentage | 40.0% | 60.0% |  |  |  |
|  |  | Lose | Frequency | 23 | 18 |  |  |  |
|  |  |  | Percentage | 56.1% | 43.9% |  |  |  |
| **High** | **ON-E** | Win | Frequency | 11 | 17 | 0.167 | 0.683 | 0.060 |
|  |  |  | Percentage | 39.3% | 60.7% |  |  |  |
|  |  | Lose | Frequency | 6 | 12 |  |  |  |
|  |  |  | Percentage | 33.3% | 66.7% |  |  |  |
| **Low** | **OF-U** | Win | Frequency | 57 | 53 | 0.710 | 0.400 | 0.058 |
|  |  |  | Percentage | 51.8% | 48.2% |  |  |  |
|  |  | Lose | Frequency | 46 | 54 |  |  |  |
|  |  |  | Percentage | 46.0% | 54.0% |  |  |  |
| **Middle** | **OF-U** | Win | Frequency | 29 | 41 | 0.373 | 0.541 | 0.050 |
|  |  |  | Percentage | 41.4% | 58.6% |  |  |  |
|  |  | Lose | Frequency | 30 | 52 |  |  |  |
|  |  |  | Percentage | 36.6% | 63.4% |  |  |  |
| **High** | **OF-U** | Win | Frequency | 18 | 22 | 0.771 | 0.380 | 0.106 |
|  |  |  | Percentage | 45.0% | 55.0% |  |  |  |
|  |  | Lose | Frequency | 10 | 19 |  |  |  |
|  |  |  | Percentage | 34.5% | 65.5% |  |  |  |
| **Low** | **OF-S** | Win | Frequency | 0 | 1 |  | 1.000 (**) | -0.250 |
|  |  |  | Percentage | 0.0% | 100.0% |  |  |  |
|  |  | Lose | Frequency | 1 | 3 |  |  |  |
|  |  |  | Percentage | 25.0% | 75.0% |  |  |  |
| **Middle** | **OF-S** | Win | Frequency | 9 | 4 |  | 0.417 (**) | 0.195 |
|  |  |  | Percentage | 69.2% | 30.8% |  |  |  |
|  |  | Lose | Frequency | 5 | 5 |  |  |  |
|  |  |  | Percentage | 50.0% | 50.0% |  |  |  |
| **High** | **OF-S** | Win | Frequency | 4 | 0 |  | 0.200 (**) | 0.535 |
|  |  |  | Percentage | 100.0% | 0.0% |  |  |  |
|  |  | Lose | Frequency | 3 | 3 |  |  |  |
|  |  |  | Percentage | 50.0% | 50.0% |  |  |  |

Low, low-point classification; Middle, middle-point classification; High, high-point classification; ON-U, the plays where the user shot using the on-the-ball screen; ON-S, plays where the screener of the on-the-ball screen shot after receiving a pass from the user; ON-A, plays where another player shot after receiving a pass from the user of the on-the-ball screen; ON-E, plays that led to a shot through two or more extra passes after the user used the on-the-ball screen; OF-U, plays where the user shot using the off-the-ball screen; OF-S, plays where the screener of the off-the-ball screen shot.

**p* < 0.05.

**We adopted *p*-value by Fisher's method.

TABLE S56 Differences in shot-success rate of the screener in movement of on-the-ball screen plays.

| **Screener** | **Movement of on-the-ball screen plays** |  |  | **Success** | **Fail** | **χ^2^(df=1)** | ***p*** | ***φ*** |
| --- | --- | --- | --- | --- | --- | --- | --- | --- |
| **Low** | **Around** | Win | Frequency | 0 | 0 |  |  |  |
|  |  |  | Percentage | 0.0% | 0.0% |  |  |  |
|  |  | Lose | Frequency | 0 | 0 |  |  |  |
|  |  |  | Percentage | 0.0% | 0.0% |  |  |  |
| **Middle** | **Around** | Win | Frequency | 3 | 8 |  | 1.000 (**) | -0.127 |
|  |  |  | Percentage | 27.3% | 72.7% |  |  |  |
|  |  | Lose | Frequency | 2 | 3 |  |  |  |
|  |  |  | Percentage | 40.0% | 60.0% |  |  |  |
| **High** | **Around** | Win | Frequency | 5 | 3 |  | 0.667 (**) | 0.151 |
|  |  |  | Percentage | 62.5% | 37.5% |  |  |  |
|  |  | Lose | Frequency | 7 | 8 |  |  |  |
|  |  |  | Percentage | 46.7% | 53.3% |  |  |  |
| **Low** | **Center-line** | Win | Frequency | 25 | 32 | 1.154 | 0.283 | 0.094 |
|  |  |  | Percentage | 43.9% | 56.1% |  |  |  |
|  |  | Lose | Frequency | 26 | 49 |  |  |  |
|  |  |  | Percentage | 34.7% | 65.3% |  |  |  |
| **Middle** | **Center-line** | Win | Frequency | 72 | 89 | 0.647 | 0.421 | 0.046 |
|  |  |  | Percentage | 44.7% | 55.3% |  |  |  |
|  |  | Lose | Frequency | 57 | 85 |  |  |  |
|  |  |  | Percentage | 40.1% | 59.9% |  |  |  |
| **High** | **Center-line** | Win | Frequency | 33 | 48 | 0.981 | 0.322 | 0.081 |
|  |  |  | Percentage | 40.7% | 59.3% |  |  |  |
|  |  | Lose | Frequency | 22 | 45 |  |  |  |
|  |  |  | Percentage | 32.8% | 67.2% |  |  |  |
| **Low** | **End-line** | Win | Frequency | 31 | 35 | 0.057 | 0.812 | 0.018 |
|  |  |  | Percentage | 47.0% | 53.0% |  |  |  |
|  |  | Lose | Frequency | 46 | 56 |  |  |  |
|  |  |  | Percentage | 45.1% | 54.9% |  |  |  |
| **Middle** | **End-line** | Win | Frequency | 78 | 115 | 0.088 | 0.767 | 0.016 |
|  |  |  | Percentage | 40.4% | 59.6% |  |  |  |
|  |  | Lose | Frequency | 61 | 96 |  |  |  |
|  |  |  | Percentage | 38.9% | 61.1% |  |  |  |
| **High** | **End-line** | Win | Frequency | 39 | 49 | 2.003 | 0.157 | 0.116 |
|  |  |  | Percentage | 44.3% | 55.7% |  |  |  |
|  |  | Lose | Frequency | 20 | 41 |  |  |  |
|  |  |  | Percentage | 32.8% | 67.2% |  |  |  |
| **Low** | **ON-Down** | Win | Frequency | 0 | 3 |  |  |  |
|  |  |  | Percentage | 0.0% | 100.0% |  |  |  |
|  |  | Lose | Frequency | 0 | 3 |  |  |  |
|  |  |  | Percentage | 0.0% | 100.0% |  |  |  |
| **Middle** | **ON-Down** | Win | Frequency | 3 | 5 |  | 1.000 (**) | 0.000 |
|  |  |  | Percentage | 37.5% | 62.5% |  |  |  |
|  |  | Lose | Frequency | 3 | 5 |  |  |  |
|  |  |  | Percentage | 37.5% | 62.5% |  |  |  |
| **High** | **ON-Down** | Win | Frequency | 1 | 3 |  | 0.524 (**) | - .350 |
|  |  |  | Percentage | 25.0% | 75.0% |  |  |  |
|  |  | Lose | Frequency | 3 | 2 |  |  |  |
|  |  |  | Percentage | 60.0% | 40.0% |  |  |  |

Low, low-point classification; Middle, middle-point classification; High, high-point classification; Around, plays where the screener held a ball; Center-line, plays where the user moved toward the center-line side against the screener; End-line, plays where the user moved toward the end-line side against the screener; ON-Down, plays where the screener was on the center-line side of the defense who protected the user holding a ball in a Top or 3P on Top extension.

**We adopted *p*-value by Fisher's method.

TABLE S57 Differences in shot-success rate of the screener in movement of off-the-ball screen plays.

| **Screener** | **Movement of off-the-ball screen plays** |  |  | **Success** | **Fail** | **χ^2^(df=1)** | ***p*** | ***φ*** |
| --- | --- | --- | --- | --- | --- | --- | --- | --- |
| **Low** | **Back** | Win | Frequency | 11 | 7 | 2.179 | 0.140 | 0.243 |
|  |  |  | Percentage | 61.1% | 38.9% |  |  |  |
|  |  | Lose | Frequency | 7 | 12 |  |  |  |
|  |  |  | Percentage | 36.8% | 63.2% |  |  |  |
| **Middle** | **Back** | Win | Frequency | 9 | 12 | 0.152 | 0.697 | 0.054 |
|  |  |  | Percentage | 42.9% | 57.1% |  |  |  |
|  |  | Lose | Frequency | 12 | 20 |  |  |  |
|  |  |  | Percentage | 37.5% | 62.5% |  |  |  |
| **High** | **Back** | Win | Frequency | 9 | 4 |  | 0.203 (**) | 0.311 |
|  |  |  | Percentage | 69.2% | 30.8% |  |  |  |
|  |  | Lose | Frequency | 3 | 5 |  |  |  |
|  |  |  | Percentage | 37.5% | 62.5% |  |  |  |
| **Low** | **Cross** | Win | Frequency | 10 | 6 |  | 1.000 (**) | -0.043 |
|  |  |  | Percentage | 62.5% | 37.5% |  |  |  |
|  |  | Lose | Frequency | 8 | 4 |  |  |  |
|  |  |  | Percentage | 66.7% | 33.3% |  |  |  |
| **Middle** | **Cross** | Win | Frequency | 5 | 2 |  | 0.633 (**) | 0.163 |
|  |  |  | Percentage | 71.4% | 28.6% |  |  |  |
|  |  | Lose | Frequency | 5 | 4 |  |  |  |
|  |  |  | Percentage | 55.6% | 44.4% |  |  |  |
| **High** | **Cross** | Win | Frequency | 2 | 0 |  | 1.000 (**) | 0.577 |
|  |  |  | Percentage | 100.0% | 0.0% |  |  |  |
|  |  | Lose | Frequency | 1 | 1 |  |  |  |
|  |  |  | Percentage | 50.0% | 50.0% |  |  |  |
| **Low** | **Down** | Win | Frequency | 14 | 11 | 3.181 | 0.074 | 0.257 |
|  |  |  | Percentage | 56.0% | 44.0% |  |  |  |
|  |  | Lose | Frequency | 7 | 16 |  |  |  |
|  |  |  | Percentage | 30.4% | 69.6% |  |  |  |
| **Middle** | **Down** | Win | Frequency | 10 | 10 | 1.997 | 0.158 | 0.213 |
|  |  |  | Percentage | 50.0% | 50.0% |  |  |  |
|  |  | Lose | Frequency | 7 | 17 |  |  |  |
|  |  |  | Percentage | 29.2% | 70.8% |  |  |  |
| **High** | **Down** | Win | Frequency | 3 | 6 |  | 1.000 (**) | 0.124 |
|  |  |  | Percentage | 33.3% | 66.7% |  |  |  |
|  |  | Lose | Frequency | 2 | 7 |  |  |  |
|  |  |  | Percentage | 22.2% | 77.8% |  |  |  |
| **Low** | **Flare** | Win | Frequency | 22 | 30 | 0.607 | 0.436 | -0.077 |
|  |  |  | Percentage | 42.3% | 57.7% |  |  |  |
|  |  | Lose | Frequency | 25 | 25 |  |  |  |
|  |  |  | Percentage | 50.0% | 50.0% |  |  |  |
| **Middle** | **Flare** | Win | Frequency | 14 | 21 | 0.003 | 0.953 | -0.007 |
|  |  |  | Percentage | 40.0% | 60.0% |  |  |  |
|  |  | Lose | Frequency | 11 | 16 |  |  |  |
|  |  |  | Percentage | 40.7% | 59.3% |  |  |  |
| **High** | **Flare** | Win | Frequency | 8 | 12 | 0.051 | 0.821 | -0.038 |
|  |  |  | Percentage | 40.0% | 60.0% |  |  |  |
|  |  | Lose | Frequency | 7 | 9 |  |  |  |
|  |  |  | Percentage | 43.8% | 56.3% |  |  |  |

Low, low-point classification; Middle, middle-point classification; High, high-point classification; Back, plays where the screener was on the end-line side of the defense who protected the user; Cross, plays where the screener was on the middle-line (the imaginary line connecting baskets running through the center of the court) side of the defense who protected the user; Down, plays where the screener on the center-line side of the defense who protected the user; Flare, plays where the screener on the side-line side of the defense who protected the user.

**We adopted *p*-value by Fisher's method.

TABLE S58 Differences in shot-success rate of the screener × the passer.

| **Screener** | **Passer** |  |  | **Success** | **Fail** | **χ^2^(df=1)** | ***p*** | ***φ*** |
| --- | --- | --- | --- | --- | --- | --- | --- | --- |
| **Low** | **Low** | Win | Frequency | 1 | 4 |  | 0.308 (**) | -0.323 |
|  |  |  | Percentage | 20.0% | 80.0% |  |  |  |
|  |  | Lose | Frequency | 6 | 5 |  |  |  |
|  |  |  | Percentage | 54.5% | 45.5% |  |  |  |
| **Low** | **Middle** | Win | Frequency | 34 | 39 | 0.558 | 0.455 | -0.062 |
|  |  |  | Percentage | 46.6% | 53.4% |  |  |  |
|  |  | Lose | Frequency | 38 | 34 |  |  |  |
|  |  |  | Percentage | 52.8% | 47.2% |  |  |  |
| **Low** | **High** | Win | Frequency | 46 | 52 | 0.751 | 0.386 | 0.058 |
|  |  |  | Percentage | 46.9% | 53.1% |  |  |  |
|  |  | Lose | Frequency | 51 | 73 |  |  |  |
|  |  |  | Percentage | 41.1% | 58.9% |  |  |  |
| **Middle** | **Low** | Win | Frequency | 2 | 9 |  | 1.000 (**) | -0.099 |
|  |  |  | Percentage | 18.2% | 81.8% |  |  |  |
|  |  | Lose | Frequency | 4 | 11 |  |  |  |
|  |  |  | Percentage | 26.7% | 73.3% |  |  |  |
| **Middle** | **Middle** | Win | Frequency | 55 | 67 | 0.001 | 0.975 | 0.002 |
|  |  |  | Percentage | 45.1% | 54.9% |  |  |  |
|  |  | Lose | Frequency | 57 | 70 |  |  |  |
|  |  |  | Percentage | 44.9% | 55.1% |  |  |  |
| **Middle** | **High** | Win | Frequency | 56 | 72 | 0.009 | 0.925 | -0.006 |
|  |  |  | Percentage | 43.8% | 56.3% |  |  |  |
|  |  | Lose | Frequency | 51 | 64 |  |  |  |
|  |  |  | Percentage | 44.3% | 55.7% |  |  |  |
| **High** | **Low** | Win | Frequency | 4 | 1 |  | 0.580 (**) | 0.244 |
|  |  |  | Percentage | 80.0% | 20.0% |  |  |  |
|  |  | Lose | Frequency | 5 | 4 |  |  |  |
|  |  |  | Percentage | 55.6% | 44.4% |  |  |  |
| **High** | **Middle** | Win | Frequency | 30 | 36 | 1.156 | 0.282 | 0.101 |
|  |  |  | Percentage | 45.5% | 54.5% |  |  |  |
|  |  | Lose | Frequency | 17 | 31 |  |  |  |
|  |  |  | Percentage | 35.4% | 64.6% |  |  |  |
| **High** | **High** | Win | Frequency | 30 | 38 | 0.999 | 0.317 | 0.091 |
|  |  |  | Percentage | 44.1% | 55.9% |  |  |  |
|  |  | Lose | Frequency | 19 | 35 |  |  |  |
|  |  |  | Percentage | 35.2% | 64.8% |  |  |  |

Low, low-point classification; Middle, middle-point classification; High, high-point classification.

**We adopted *p*-value by Fisher's method.

TABLE S59 Differences in shot-success rate depending on the PC of the passer.

| **Passer** |  |  | **Success** | **Fail** | **χ^2^(df=1)** | ***p*** | ***φ*** |
| --- | --- | --- | --- | --- | --- | --- | --- |
| **Low** | Win | Frequency | 8 | 14 | 0.237 | 0.627 | -0.064 |
|  |  | Percentage | 36.4% | 63.6% |  |  |  |
|  | Lose | Frequency | 15 | 20 |  |  |  |
|  |  | Percentage | 42.9% | 57.1% |  |  |  |
| **Middle** | Win | Frequency | 121 | 143 | 0.012 | 0.912 | 0.005 |
|  |  | Percentage | 45.8% | 54.2% |  |  |  |
|  | Lose | Frequency | 112 | 135 |  |  |  |
|  |  | Percentage | 45.3% | 54.7% |  |  |  |
| **High** | Win | Frequency | 133 | 163 | 0.858 | 0.354 | 0.038 |
|  |  | Percentage | 44.9% | 55.1% |  |  |  |
|  | Lose | Frequency | 121 | 173 |  |  |  |
|  |  | Percentage | 41.2% | 58.8% |  |  |  |

PC, player classification; Low, low-point classification; Middle, middle-point classification; High, high-point classification.

TABLE S60 Differences in shot-success rate of the passer in shot location.

| **Passer** | **Shot Location** |  |  | **Success** | **Fail** | **χ^2^(df=1)** | ***p*** | ***φ*** |
| --- | --- | --- | --- | --- | --- | --- | --- | --- |
| **Low** | **PL** | Win | Frequency | 2 | 5 |  | 0.622 (**) | -0.214 |
|  |  |  | Percentage | 28.6% | 71.4% |  |  |  |
|  |  | Lose | Frequency | 5 | 5 |  |  |  |
|  |  |  | Percentage | 50.0% | 50.0% |  |  |  |
| **Middle** | **PL** | Win | Frequency | 48 | 34 | 0.463 | 0.496 | -0.053 |
|  |  |  | Percentage | 58.5% | 41.5% |  |  |  |
|  |  | Lose | Frequency | 51 | 29 |  |  |  |
|  |  |  | Percentage | 63.7% | 36.3% |  |  |  |
| **High** | **PL** | Win | Frequency | 59 | 49 | 0.001 | 0.972 | -0.002 |
|  |  |  | Percentage | 54.6% | 45.4% |  |  |  |
|  |  | Lose | Frequency | 62 | 51 |  |  |  |
|  |  |  | Percentage | 54.9% | 45.1% |  |  |  |
| **Low** | **PH** | Win | Frequency | 2 | 3 |  |  |  |
|  |  |  | Percentage | 40.0% | 60.0% |  |  |  |
|  |  | Lose | Frequency | 2 | 3 |  |  |  |
|  |  |  | Percentage | 40.0% | 60.0% |  |  |  |
| **Middle** | **PH** | Win | Frequency | 12 | 10 | 1.773 | 0.183 | 0.188 |
|  |  |  | Percentage | 54.5% | 45.5% |  |  |  |
|  |  | Lose | Frequency | 10 | 18 |  |  |  |
|  |  |  | Percentage | 35.7% | 64.3% |  |  |  |
| **High** | **PH** | Win | Frequency | 12 | 11 | 3.725 | 0.054 | 0.291 |
|  |  |  | Percentage | 52.2% | 47.8% |  |  |  |
|  |  | Lose | Frequency | 5 | 16 |  |  |  |
|  |  |  | Percentage | 23.8% | 76.2% |  |  |  |
| **Low** | **Top** | Win | Frequency | 0 | 0 |  |  |  |
|  |  |  | Percentage | 0.0% | 0.0% |  |  |  |
|  |  | Lose | Frequency | 1 | 0 |  |  |  |
|  |  |  | Percentage | 100.0% | 0.0% |  |  |  |
| **Middle** | **Top** | Win | Frequency | 10 | 17 | 0.145 | 0.704 | -0.050 |
|  |  |  | Percentage | 37.0% | 63.0% |  |  |  |
|  |  | Lose | Frequency | 13 | 18 |  |  |  |
|  |  |  | Percentage | 41.9% | 58.1% |  |  |  |
| **High** | **Top** | Win | Frequency | 21 | 30 | 0.038 | 0.846 | -0.021 |
|  |  |  | Percentage | 41.2% | 58.8% |  |  |  |
|  |  | Lose | Frequency | 16 | 21 |  |  |  |
|  |  |  | Percentage | 43.2% | 56.8% |  |  |  |
| **Low** | **Corner** | Win | Frequency | 5 | 3 | 0.281 | 0.596 | 0.125 |
|  |  |  | Percentage | 62.5% | 37.5% |  |  |  |
|  |  | Lose | Frequency | 5 | 5 |  |  |  |
|  |  |  | Percentage | 50.0% | 50.0% |  |  |  |
| **Middle** | **Corner** | Win | Frequency | 24 | 31 | 0.435 | 0.510 | -0.064 |
|  |  |  | Percentage | 43.6% | 56.4% |  |  |  |
|  |  | Lose | Frequency | 26 | 26 |  |  |  |
|  |  |  | Percentage | 50.0% | 50.0% |  |  |  |
| **High** | **Corner** | Win | Frequency | 19 | 33 | 0.820 | 0.365 | -0.083 |
|  |  |  | Percentage | 36.5% | 63.5% |  |  |  |
|  |  | Lose | Frequency | 30 | 37 |  |  |  |
|  |  |  | Percentage | 44.8% | 55.2% |  |  |  |
| **Low** | **Wing** | Win | Frequency | 1 | 6 |  | 1.000 (**) | -0.033 |
|  |  |  | Percentage | 14.3% | 85.7% |  |  |  |
|  |  | Lose | Frequency | 1 | 5 |  |  |  |
|  |  |  | Percentage | 16.7% | 83.3% |  |  |  |
| **Middle** | **Wing** | Win | Frequency | 13 | 32 | 0.060 | 0.806 | -0.027 |
|  |  |  | Percentage | 28.9% | 71.1% |  |  |  |
|  |  | Lose | Frequency | 11 | 24 |  |  |  |
|  |  |  | Percentage | 31.4% | 68.6% |  |  |  |
| **High** | **Wing** | Win | Frequency | 19 | 23 | 5.696* | 0.017 | 0.277 |
|  |  |  | Percentage | 45.2% | 54.8% |  |  |  |
|  |  | Lose | Frequency | 6 | 26 |  |  |  |
|  |  |  | Percentage | 18.8% | 81.3% |  |  |  |
| **Low** | **3P** | Win | Frequency | 1 | 2 |  |  |  |
|  |  |  | Percentage | 33.3% | 66.7% |  |  |  |
|  |  | Lose | Frequency | 1 | 2 |  |  |  |
|  |  |  | Percentage | 33.3% | 66.7% |  |  |  |
| **Middle** | **3P** | Win | Frequency | 14 | 19 | 9.074* | 0.003 | 0.410 |
|  |  |  | Percentage | 42.4% | 57.6% |  |  |  |
|  |  | Lose | Frequency | 1 | 20 |  |  |  |
|  |  |  | Percentage | 4.8% | 95.2% |  |  |  |
| **High** | **3P** | Win | Frequency | 3 | 17 |  | 0.646 (**) | 0.105 |
|  |  |  | Percentage | 15.0% | 85.0% |  |  |  |
|  |  | Lose | Frequency | 2 | 22 |  |  |  |
|  |  |  | Percentage | 8.3% | 91.7% |  |  |  |

Low, low-point classification; Middle, middle-point classification; High, high-point classification; PL, paint-low; PH, paint-high; 3P, 3-point field goal area.

**p* < 0.05.

**We adopted *p*-value by Fisher's method.

TABLE S61 Differences in shot-success rate of the passer in screen location.

| **Passer** | **Screen Location** |  |  | **Success** | **Fail** | **χ^2^(df=1)** | ***p*** | ***φ*** |
| --- | --- | --- | --- | --- | --- | --- | --- | --- |
| **Low** | **PL** | Win | frequency | 0 | 1 |  | 1.000 (**) | -0.500 |
|  |  |  | percentage | 0.0% | 100.0% |  |  |  |
|  |  | Lose | Frequency | 1 | 1 |  |  |  |
|  |  |  | Percentage | 50.0% | 50.0% |  |  |  |
| **Middle** | **PL** | Win | Frequency | 11 | 2 |  | 0.378 (**) | 0.210 |
|  |  |  | Percentage | 84.6% | 15.4% |  |  |  |
|  |  | Lose | Frequency | 8 | 4 |  |  |  |
|  |  |  | Percentage | 66.7% | 33.3% |  |  |  |
| **High** | **PL** | Win | Frequency | 10 | 7 | 0.889 | 0.346 | 0.146 |
|  |  |  | Percentage | 58.8% | 41.2% |  |  |  |
|  |  | Lose | Frequency | 11 | 14 |  |  |  |
|  |  |  | Percentage | 44.0% | 56.0% |  |  |  |
| **Low** | **PH** | Win | Frequency | 1 | 0 |  | 1.000 (**) | 1.000 |
|  |  |  | Percentage | 100.0% | 0.0% |  |  |  |
|  |  | Lose | Frequency | 0 | 1 |  |  |  |
|  |  |  | Percentage | 0.0% | 100.0% |  |  |  |
| **Middle** | **PH** | Win | Frequency | 12 | 20 | 2.163 | 0.141 | -0.219 |
|  |  |  | Percentage | 37.5% | 62.5% |  |  |  |
|  |  | Lose | Frequency | 8 | 5 |  |  |  |
|  |  |  | Percentage | 61.5% | 38.5% |  |  |  |
| **High** | **PH** | Win | Frequency | 14 | 16 | 0.135 | 0.713 | 0.050 |
|  |  |  | Percentage | 46.7% | 53.3% |  |  |  |
|  |  | Lose | Frequency | 10 | 14 |  |  |  |
|  |  |  | Percentage | 41.7% | 58.3% |  |  |  |
| **Low** | **Top** | Win | Frequency | 0 | 1 |  | 1.000 (**) | -0.577 |
|  |  |  | Percentage | 0.0% | 100.0% |  |  |  |
|  |  | Lose | Frequency | 2 | 1 |  |  |  |
|  |  |  | Percentage | 66.7% | 33.3% |  |  |  |
| **Middle** | **Top** | Win | Frequency | 7 | 6 | 1.242 | 0.265 | 0.186 |
|  |  |  | Percentage | 53.8% | 46.2% |  |  |  |
|  |  | Lose | Frequency | 8 | 15 |  |  |  |
|  |  |  | Percentage | 34.8% | 65.2% |  |  |  |
| **High** | **Top** | Win | Frequency | 9 | 6 | 1.540 | 0.215 | 0.181 |
|  |  |  | Percentage | 60.0% | 40.0% |  |  |  |
|  |  | Lose | Frequency | 13 | 19 |  |  |  |
|  |  |  | Percentage | 40.6% | 59.4% |  |  |  |
| **Low** | **Corner** | Win | Frequency | 2 | 8 |  | 0.204 (**) | -0.311 |
|  |  |  | Percentage | 20.0% | 80.0% |  |  |  |
|  |  | Lose | Frequency | 6 | 6 |  |  |  |
|  |  |  | Percentage | 50.0% | 50.0% |  |  |  |
| **Middle** | **Corner** | Win | Frequency | 32 | 33 | 0.031 | 0.861 | -0.015 |
|  |  |  | Percentage | 49.2% | 50.8% |  |  |  |
|  |  | Lose | Frequency | 33 | 32 |  |  |  |
|  |  |  | Percentage | 50.8% | 49.2% |  |  |  |
| **High** | **Corner** | Win | Frequency | 41 | 47 | 0.423 | 0.516 | 0.050 |
|  |  |  | Percentage | 46.6% | 53.4% |  |  |  |
|  |  | Lose | Frequency | 35 | 49 |  |  |  |
|  |  |  | Percentage | 41.7% | 58.3% |  |  |  |
| **Low** | **Wing** | Win | Frequency | 4 | 4 |  | 0.662 (**) | 0.140 |
|  |  |  | Percentage | 50.0% | 50.0% |  |  |  |
|  |  | Lose | Frequency | 5 | 9 |  |  |  |
|  |  |  | Percentage | 35.7% | 64.3% |  |  |  |
| **Middle** | **Wing** | Win | Frequency | 46 | 73 | 0.145 | 0.704 | 0.025 |
|  |  |  | Percentage | 38.7% | 61.3% |  |  |  |
|  |  | Lose | Frequency | 38 | 67 |  |  |  |
|  |  |  | Percentage | 36.2% | 63.8% |  |  |  |
| **High** | **Wing** | Win | Frequency | 49 | 75 | 0.047 | 0.828 | -0.014 |
|  |  |  | Percentage | 39.5% | 60.5% |  |  |  |
|  |  | Lose | Frequency | 45 | 65 |  |  |  |
|  |  |  | Percentage | 40.9% | 59.1% |  |  |  |
| **Low** | **3P** | Win | Frequency | 0 | 0 |  |  |  |
|  |  |  | Percentage | 0.0% | 0.0% |  |  |  |
|  |  | Lose | Frequency | 1 | 2 |  |  |  |
|  |  |  | Percentage | 33.3% | 66.7% |  |  |  |
| **Middle** | **3P** | Win | Frequency | 11 | 8 | 0.002 | 0.960 | -0.007 |
|  |  |  | Percentage | 57.9% | 42.1% |  |  |  |
|  |  | Lose | Frequency | 17 | 12 |  |  |  |
|  |  |  | Percentage | 58.6% | 41.4% |  |  |  |
| **High** | **3P** | Win | Frequency | 9 | 11 | 0.145 | 0.703 | 0.062 |
|  |  |  | Percentage | 45.0% | 55.0% |  |  |  |
|  |  | Lose | Frequency | 7 | 11 |  |  |  |
|  |  |  | Percentage | 38.9% | 61.1% |  |  |  |

Low, low-point classification; Middle, middle-point classification; High, high-point classification; PL, paint-low; PH, paint-high; 3P, 3-point field goal area.

**We adopted *p*-value by Fisher's method.

TABLE S62 Differences in shot-success rate of the passer in pass location.

| **Passer** | **Pass Location** |  |  | **Success** | **Fail** | **χ^2^(df=1)** | ***p*** | ***φ*** |
| --- | --- | --- | --- | --- | --- | --- | --- | --- |
| **Low** | **PL** | Win | Frequency | 0 | 2 |  | 1.000 (**) | -0.316 |
|  |  |  | Percentage | 0.0% | 100.0% |  |  |  |
|  |  | Lose | Frequency | 1 | 3 |  |  |  |
|  |  |  | Percentage | 25.0% | 75.0% |  |  |  |
| **Middle** | **PL** | Win | Frequency | 6 | 3 |  | 1.000 (**) | -0.051 |
|  |  |  | Percentage | 66.7% | 33.3% |  |  |  |
|  |  | Lose | Frequency | 5 | 2 |  |  |  |
|  |  |  | Percentage | 71.4% | 28.6% |  |  |  |
| **High** | **PL** | Win | Frequency | 5 | 6 |  | 1.000 (**) | 0.255 |
|  |  |  | Percentage | 45.5% | 54.5% |  |  |  |
|  |  | Lose | Frequency | 0 | 1 |  |  |  |
|  |  |  | Percentage | 0.0% | 100.0% |  |  |  |
| **Low** | **PH** | Win | Frequency | 0 | 1 |  | 1.000 (**) | -0.408 |
|  |  |  | Percentage | 0.0% | 100.0% |  |  |  |
|  |  | Lose | Frequency | 2 | 2 |  |  |  |
|  |  |  | Percentage | 50.0% | 50.0% |  |  |  |
| **Middle** | **PH** | Win | Frequency | 17 | 19 | 0.040 | 0.842 | -0.027 |
|  |  |  | Percentage | 47.2% | 52.8% |  |  |  |
|  |  | Lose | Frequency | 10 | 10 |  |  |  |
|  |  |  | Percentage | 50.0% | 50.0% |  |  |  |
| **High** | **PH** | Win | Frequency | 24 | 24 | 3.130 | 0.077 | 0.184 |
|  |  |  | Percentage | 50.0% | 50.0% |  |  |  |
|  |  | Lose | Frequency | 14 | 30 |  |  |  |
|  |  |  | Percentage | 31.8% | 68.2% |  |  |  |
| **Low** | **Top** | Win | Frequency | 5 | 7 | 0.001 | 0.981 | -0.004 |
|  |  |  | Percentage | 41.7% | 58.3% |  |  |  |
|  |  | Lose | Frequency | 8 | 11 |  |  |  |
|  |  |  | Percentage | 42.1% | 57.9% |  |  |  |
| **Middle** | **Top** | Win | Frequency | 15 | 26 | 2.095 | 0.148 | -0.159 |
|  |  |  | Percentage | 36.6% | 63.4% |  |  |  |
|  |  | Lose | Frequency | 22 | 20 |  |  |  |
|  |  |  | Percentage | 52.4% | 47.6% |  |  |  |
| **High** | **Top** | Win | Frequency | 19 | 23 | 0.860 | 0.354 | -0.096 |
|  |  |  | Percentage | 45.2% | 54.8% |  |  |  |
|  |  | Lose | Frequency | 28 | 23 |  |  |  |
|  |  |  | Percentage | 54.9% | 45.1% |  |  |  |
| **Low** | **Corner** | Win | Frequency | 1 | 3 |  | 1.000 (**) | -0.091 |
|  |  |  | Percentage | 25.0% | 75.0% |  |  |  |
|  |  | Lose | Frequency | 1 | 2 |  |  |  |
|  |  |  | Percentage | 33.3% | 66.7% |  |  |  |
| **Middle** | **Corner** | Win | Frequency | 23 | 29 | 0.008 | 0.929 | -0.009 |
|  |  |  | Percentage | 44.2% | 55.8% |  |  |  |
|  |  | Lose | Frequency | 23 | 28 |  |  |  |
|  |  |  | Percentage | 45.1% | 54.9% |  |  |  |
| **High** | **Corner** | Win | Frequency | 31 | 43 | 0.019 | 0.891 | -0.013 |
|  |  |  | Percentage | 41.9% | 58.1% |  |  |  |
|  |  | Lose | Frequency | 19 | 25 |  |  |  |
|  |  |  | Percentage | 43.2% | 56.8% |  |  |  |
| **Low** | **Wing** | Win | Frequency | 1 | 0 |  |  |  |
|  |  |  | Percentage | 100.0% | 0.0% |  |  |  |
|  |  | Lose | Frequency | 2 | 0 |  |  |  |
|  |  |  | Percentage | 100.0% | 0.0% |  |  |  |
| **Middle** | **Wing** | Win | Frequency | 32 | 42 | 0.007 | 0.934 | -0.007 |
|  |  |  | Percentage | 43.2% | 56.8% |  |  |  |
|  |  | Lose | Frequency | 29 | 37 |  |  |  |
|  |  |  | Percentage | 43.9% | 56.1% |  |  |  |
| **High** | **Wing** | Win | Frequency | 36 | 48 | 0.054 | 0.817 | -0.017 |
|  |  |  | Percentage | 42.9% | 57.1% |  |  |  |
|  |  | Lose | Frequency | 45 | 56 |  |  |  |
|  |  |  | Percentage | 44.6% | 55.4% |  |  |  |
| **Low** | **3P** | Win | Frequency | 1 | 1 |  | 1.000 (**) | 0.167 |
|  |  |  | Percentage | 50.0% | 50.0% |  |  |  |
|  |  | Lose | Frequency | 1 | 2 |  |  |  |
|  |  |  | Percentage | 33.3% | 66.7% |  |  |  |
| **Middle** | **3P** | Win | Frequency | 28 | 24 | 2.953 | 0.086 | 0.162 |
|  |  |  | Percentage | 53.8% | 46.2% |  |  |  |
|  |  | Lose | Frequency | 23 | 38 |  |  |  |
|  |  |  | Percentage | 37.7% | 62.3% |  |  |  |
| **High** | **3P** | Win | Frequency | 18 | 19 | 3.884* | 0.049 | 0.208 |
|  |  |  | Percentage | 48.6% | 51.4% |  |  |  |
|  |  | Lose | Frequency | 15 | 38 |  |  |  |
|  |  |  | Percentage | 28.3% | 71.7% |  |  |  |

Low, low-point classification; Middle, middle-point classification; High, high-point classification; PL, paint-low; PH, paint-high; 3P, 3-point field goal area.

**p* < 0.05.

**We adopted *p*-value by Fisher's method.

TABLE S63 Differences in shot-success rate of the passer in type of screen.

| **Passer** | **Type of Screen** |  |  | **Success** | **Fail** | **χ^2^(df=1)** | ***p*** | ***φ*** |
| --- | --- | --- | --- | --- | --- | --- | --- | --- |
| **Low** | **On-the-ball screen** | Win | Frequency | 0 | 6 |  | 0.066 (**) | -0.408 |
|  |  |  | Percentage | 0.0% | 100.0% |  |  |  |
|  |  | Lose | Frequency | 8 | 10 |  |  |  |
|  |  |  | Percentage | 44.4% | 55.6% |  |  |  |
| **Middle** | **On-the-ball screen** | Win | Frequency | 64 | 82 | 0.048 | 0.826 | -0.013 |
|  |  |  | Percentage | 43.8% | 56.2% |  |  |  |
|  |  | Lose | Frequency | 69 | 84 |  |  |  |
|  |  |  | Percentage | 45.1% | 54.9% |  |  |  |
| **High** | **On-the-ball screen** | Win | Frequency | 77 | 109 | 0.235 | 0.628 | -0.026 |
|  |  |  | Percentage | 41.4% | 58.6% |  |  |  |
|  |  | Lose | Frequency | 76 | 97 |  |  |  |
|  |  |  | Percentage | 43.9% | 56.1% |  |  |  |
| **Low** | **Off-the-ball screen** | Win | Frequency | 8 | 8 | 0.259 | 0.611 | 0.089 |
|  |  |  | Percentage | 50.0% | 50.0% |  |  |  |
|  |  | Lose | Frequency | 7 | 10 |  |  |  |
|  |  |  | Percentage | 41.2% | 58.8% |  |  |  |
| **Middle** | **Off-the-ball screen** | Win | Frequency | 57 | 61 | 0.138 | 0.711 | 0.025 |
|  |  |  | Percentage | 48.3% | 51.7% |  |  |  |
|  |  | Lose | Frequency | 43 | 51 |  |  |  |
|  |  |  | Percentage | 45.7% | 54.3% |  |  |  |
| **High** | **Off-the-ball screen** | Win | Frequency | 56 | 54 | 4.407* | 0.036 | 0.138 |
|  |  |  | Percentage | 50.9% | 49.1% |  |  |  |
|  |  | Lose | Frequency | 45 | 76 |  |  |  |
|  |  |  | Percentage | 37.2% | 62.8% |  |  |  |

Low, low-point classification; Middle, middle-point classification; High, high-point classification.

**p* < 0.05.

**We adopted *p*-value by Fisher's method.

TABLE S64 Differences in shot-success rate of the passer in type of screen-play.

| **Passer** | **Type of Screen-play** |  |  | **Success** | **Fail** | **χ^2^(df=1)** | ***p*** | ***φ*** |
| --- | --- | --- | --- | --- | --- | --- | --- | --- |
| **Low** | **ON-U** | Win | Frequency | 0 | 0 |  |  |  |
|  |  |  | Percentage | 0.0% | 0.0% |  |  |  |
|  |  | Lose | Frequency | 0 | 0 |  |  |  |
|  |  |  | Percentage | 0.0% | 0.0% |  |  |  |
| **Middle** | **ON-U** | Win | Frequency | 2 | 5 |  | 1.000 (**) | -0.189 |
|  |  |  | Percentage | 28.6% | 71.4% |  |  |  |
|  |  | Lose | Frequency | 1 | 1 |  |  |  |
|  |  |  | Percentage | 50.0% | 50.0% |  |  |  |
| **High** | **ON-U** | Win | Frequency | 3 | 0 |  | 0.231 (**) | 0.433 |
|  |  |  | Percentage | 100.0% | 0.0% |  |  |  |
|  |  | Lose | Frequency | 5 | 5 |  |  |  |
|  |  |  | Percentage | 50.0% | 50.0% |  |  |  |
| **Low** | **ON-S** | Win | Frequency | 0 | 0 |  |  |  |
|  |  |  | Percentage | 0.0% | 0.0% |  |  |  |
|  |  | Lose | Frequency | 0 | 2 |  |  |  |
|  |  |  | Percentage | 0.0% | 100.0% |  |  |  |
| **Middle** | **ON-S** | Win | Frequency | 11 | 19 | 3.699 | 0.054 | -0.246 |
|  |  |  | Percentage | 36.7% | 63.3% |  |  |  |
|  |  | Lose | Frequency | 19 | 12 |  |  |  |
|  |  |  | Percentage | 61.3% | 38.7% |  |  |  |
| **High** | **ON-S** | Win | Frequency | 18 | 17 | 0.342 | 0.559 | -0.069 |
|  |  |  | Percentage | 51.4% | 48.6% |  |  |  |
|  |  | Lose | Frequency | 21 | 15 |  |  |  |
|  |  |  | Percentage | 58.3% | 41.7% |  |  |  |
| **Low** | **ON-A** | Win | Frequency | 0 | 1 |  | 1.000 (**) | -0.500 |
|  |  |  | Percentage | 0.0% | 100.0% |  |  |  |
|  |  | Lose | Frequency | 1 | 1 |  |  |  |
|  |  |  | Percentage | 50.0% | 50.0% |  |  |  |
| **Middle** | **ON-A** | Win | Frequency | 29 | 31 | 3.443 | 0.064 | 0.156 |
|  |  |  | Percentage | 48.3% | 51.7% |  |  |  |
|  |  | Lose | Frequency | 27 | 55 |  |  |  |
|  |  |  | Percentage | 32.9% | 67.1% |  |  |  |
| **High** | **ON-A** | Win | Frequency | 45 | 72 | 0.104 | 0.748 | -0.022 |
|  |  |  | Percentage | 38.5% | 61.5% |  |  |  |
|  |  | Lose | Frequency | 37 | 54 |  |  |  |
|  |  |  | Percentage | 40.7% | 59.3% |  |  |  |
| **Low** | **ON-E** | Win | Frequency | 0 | 5 |  | 0.106 (**) | -0.456 |
|  |  |  | Percentage | 0.0% | 100.0% |  |  |  |
|  |  | Lose | Frequency | 7 | 7 |  |  |  |
|  |  |  | Percentage | 50.0% | 50.0% |  |  |  |
| **Middle** | **ON-E** | Win | Frequency | 22 | 27 | 1.446 | 0.229 | -0.129 |
|  |  |  | Percentage | 44.9% | 55.1% |  |  |  |
|  |  | Lose | Frequency | 22 | 16 |  |  |  |
|  |  |  | Percentage | 57.9% | 42.1% |  |  |  |
| **High** | **ON-E** | Win | Frequency | 11 | 20 | 0.003 | 0.957 | -0.007 |
|  |  |  | Percentage | 35.5% | 64.5% |  |  |  |
|  |  | Lose | Frequency | 13 | 23 |  |  |  |
|  |  |  | Percentage | 36.1% | 63.9% |  |  |  |
| **Low** | **OF-U** | Win | Frequency | 7 | 8 | 0.032 | 0.858 | -0.033 |
|  |  |  | Percentage | 46.7% | 53.3% |  |  |  |
|  |  | Lose | Frequency | 7 | 7 |  |  |  |
|  |  |  | Percentage | 50.0% | 50.0% |  |  |  |
| **Middle** | **OF-U** | Win | Frequency | 49 | 58 | 0.067 | 0.796 | 0.019 |
|  |  |  | Percentage | 45.8% | 54.2% |  |  |  |
|  |  | Lose | Frequency | 36 | 46 |  |  |  |
|  |  |  | Percentage | 43.9% | 56.1% |  |  |  |
| **High** | **OF-U** | Win | Frequency | 52 | 52 | 3.737 | 0.053 | 0.130 |
|  |  |  | Percentage | 50.0% | 50.0% |  |  |  |
|  |  | Lose | Frequency | 43 | 73 |  |  |  |
|  |  |  | Percentage | 37.1% | 62.9% |  |  |  |
| **Low** | **OF-S** | Win | Frequency | 1 | 0 |  | 0.250 (**) | 1.000 |
|  |  |  | Percentage | 100.0% | 0.0% |  |  |  |
|  |  | Lose | Frequency | 0 | 3 |  |  |  |
|  |  |  | Percentage | 0.0% | 100.0% |  |  |  |
| **Middle** | **OF-S** | Win | Frequency | 8 | 3 |  | 0.667 (**) | 0.151 |
|  |  |  | Percentage | 72.7% | 27.3% |  |  |  |
|  |  | Lose | Frequency | 7 | 5 |  |  |  |
|  |  |  | Percentage | 58.3% | 41.7% |  |  |  |
| **High** | **OF-S** | Win | Frequency | 4 | 2 |  | 0.567 (**) | 0.267 |
|  |  |  | Percentage | 66.7% | 33.3% |  |  |  |
|  |  | Lose | Frequency | 2 | 3 |  |  |  |
|  |  |  | Percentage | 40.0% | 60.0% |  |  |  |

Low, low-point classification; Middle, middle-point classification; High, high-point classification; ON-U, the plays where the user shot using the on-the-ball screen; ON-S, plays where the screener of the on-the-ball screen shot after receiving a pass from the user; ON-A, plays where another player shot after receiving a pass from the user of the on-the-ball screen; ON-E, plays that led to a shot through two or more extra passes after the user used the on-the-ball screen; OF-U, plays where the user shot using the off-the-ball screen; OF-S, plays where the screener of the off-the-ball screen shot.

**We adopted *p*-value by Fisher's method.

TABLE S65 Differences in shot-success rate of the passer in movement of on-the-ball screen plays.

| **Passer** | **Movement of on-the-ball screen plays** |  |  | **Success** | **Fail** | **χ^2^(df=1)** | ***p*** | ***φ*** |
| --- | --- | --- | --- | --- | --- | --- | --- | --- |
| **Low** | **Around** | Win | Frequency | 0 | 0 |  |  |  |
|  |  |  | Percentage | 0.0% | 0.0% |  |  |  |
|  |  | Lose | Frequency | 0 | 0 |  |  |  |
|  |  |  | Percentage | 0.0% | 0.0% |  |  |  |
| **Middle** | **Around** | Win | Frequency | 3 | 6 |  | 1.000 (**) | 0.083 |
|  |  |  | Percentage | 33.3% | 66.7% |  |  |  |
|  |  | Lose | Frequency | 1 | 3 |  |  |  |
|  |  |  | Percentage | 25.0% | 75.0% |  |  |  |
| **High** | **Around** | Win | Frequency | 3 | 3 |  | 1.000 (**) | 0.000 |
|  |  |  | Percentage | 50.0% | 50.0% |  |  |  |
|  |  | Lose | Frequency | 7 | 7 |  |  |  |
|  |  |  | Percentage | 50.0% | 50.0% |  |  |  |
| **Low** | **Center-line** | Win | Frequency | 0 | 1 |  | 1.000 (**) | -0.378 |
|  |  |  | Percentage | 0.0% | 100.0% |  |  |  |
|  |  | Lose | Frequency | 4 | 3 |  |  |  |
|  |  |  | Percentage | 57.1% | 42.9% |  |  |  |
| **Middle** | **Center-line** | Win | Frequency | 32 | 37 | 0.032 | 0.858 | -0.015 |
|  |  |  | Percentage | 46.4% | 53.6% |  |  |  |
|  |  | Lose | Frequency | 34 | 37 |  |  |  |
|  |  |  | Percentage | 47.9% | 52.1% |  |  |  |
| **High** | **Center-line** | Win | Frequency | 41 | 49 | 0.004 | 0.949 | -0.005 |
|  |  |  | Percentage | 45.6% | 54.4% |  |  |  |
|  |  | Lose | Frequency | 35 | 41 |  |  |  |
|  |  |  | Percentage | 46.1% | 53.9% |  |  |  |
| **Low** | **End-line** | Win | Frequency | 0 | 5 |  | 0.245 (**) | -0.389 |
|  |  |  | Percentage | 0.0% | 100.0% |  |  |  |
|  |  | Lose | Frequency | 4 | 7 |  |  |  |
|  |  |  | Percentage | 36.4% | 63.6% |  |  |  |
| **Middle** | **End-line** | Win | Frequency | 29 | 39 | 0.009 | 0.925 | -0.008 |
|  |  |  | Percentage | 42.6% | 57.4% |  |  |  |
|  |  | Lose | Frequency | 33 | 43 |  |  |  |
|  |  |  | Percentage | 43.4% | 56.6% |  |  |  |
| **High** | **End-line** | Win | Frequency | 32 | 56 | 0.381 | 0.537 | -0.047 |
|  |  |  | Percentage | 36.4% | 63.6% |  |  |  |
|  |  | Lose | Frequency | 34 | 49 |  |  |  |
|  |  |  | Percentage | 41.0% | 59.0% |  |  |  |
| **Low** | **ON-Down** | Win | Frequency | 0 | 0 |  |  |  |
|  |  |  | Percentage | 0.0% | 0.0% |  |  |  |
|  |  | Lose | Frequency | 0 | 0 |  |  |  |
|  |  |  | Percentage | 0.0% | 0.0% |  |  |  |
| **Middle** | **ON-Down** | Win | Frequency | 0 | 0 |  |  |  |
|  |  |  | Percentage | 0.0% | 0.0% |  |  |  |
|  |  | Lose | Frequency | 1 | 1 |  |  |  |
|  |  |  | Percentage | 50.0% | 50.0% |  |  |  |
| **High** | **ON-Down** | Win | Frequency | 1 | 1 |  |  |  |
|  |  |  | Percentage | 50.0% | 50.0% |  |  |  |
|  |  | Lose | Frequency | 0 | 0 |  |  |  |
|  |  |  | Percentage | 0.0% | 0.0% |  |  |  |

Low, low-point classification; Middle, middle-point classification; High, high-point classification; Around, plays where the screener held a ball; Center-line, plays where the user moved toward the center-line side against the screener; End-line, plays where the user moved toward the end-line side against the screener; ON-Down, plays where the screener was on the center-line side of the defense who protected the user holding a ball in a Top or 3P on Top extension.

**We adopted *p*-value by Fisher's method.

TABLE S66 Differences in shot-success rate of the passer in movement of off-the-ball screen plays.

| **Passer** | **Movement of off-the-ball screen plays** |  |  | **Success** | **Fail** | **χ^2^(df=1)** | ***p*** | ***φ*** |
| --- | --- | --- | --- | --- | --- | --- | --- | --- |
| **Low** | **Back** | Win | Frequency | 3 | 1 |  | 1.000 (**) | 0.258 |
|  |  |  | Percentage | 75.0% | 25.0% |  |  |  |
|  |  | Lose | Frequency | 2 | 2 |  |  |  |
|  |  |  | Percentage | 50.0% | 50.0% |  |  |  |
| **Middle** | **Back** | Win | Frequency | 16 | 10 | 4.464* | 0.035 | 0.296 |
|  |  |  | Percentage | 61.5% | 38.5% |  |  |  |
|  |  | Lose | Frequency | 8 | 17 |  |  |  |
|  |  |  | Percentage | 32.0% | 68.0% |  |  |  |
| **High** | **Back** | Win | Frequency | 10 | 12 | 0.155 | 0.694 | 0.055 |
|  |  |  | Percentage | 45.5% | 54.5% |  |  |  |
|  |  | Lose | Frequency | 12 | 18 |  |  |  |
|  |  |  | Percentage | 40.0% | 60.0% |  |  |  |
| **Low** | **Cross** | Win | Frequency | 1 | 1 |  | 0.400 (**) | 0.612 |
|  |  |  | Percentage | 50.0% | 50.0% |  |  |  |
|  |  | Lose | Frequency | 0 | 3 |  |  |  |
|  |  |  | Percentage | 0.0% | 100.0% |  |  |  |
| **Middle** | **Cross** | Win | Frequency | 7 | 4 |  | 0.338 (**) | -0.268 |
|  |  |  | Percentage | 63.6% | 36.4% |  |  |  |
|  |  | Lose | Frequency | 7 | 1 |  |  |  |
|  |  |  | Percentage | 87.5% | 12.5% |  |  |  |
| **High** | **Cross** | Win | Frequency | 9 | 3 |  | 0.667 (**) | 0.177 |
|  |  |  | Percentage | 75.0% | 25.0% |  |  |  |
|  |  | Lose | Frequency | 7 | 5 |  |  |  |
|  |  |  | Percentage | 58.3% | 41.7% |  |  |  |
| **Low** | **Down** | Win | Frequency | 0 | 2 |  | 0.400 (**) | -0.707 |
|  |  |  | Percentage | 0.0% | 100.0% |  |  |  |
|  |  | Lose | Frequency | 3 | 1 |  |  |  |
|  |  |  | Percentage | 75.0% | 25.0% |  |  |  |
| **Middle** | **Down** | Win | Frequency | 15 | 12 | 3.495 | 0.062 | 0.270 |
|  |  |  | Percentage | 55.6% | 44.4% |  |  |  |
|  |  | Lose | Frequency | 6 | 15 |  |  |  |
|  |  |  | Percentage | 28.6% | 71.4% |  |  |  |
| **High** | **Down** | Win | Frequency | 12 | 13 | 3.989* | 0.046 | 0.267 |
|  |  |  | Percentage | 48.0% | 52.0% |  |  |  |
|  |  | Lose | Frequency | 7 | 24 |  |  |  |
|  |  |  | Percentage | 22.6% | 77.4% |  |  |  |
| **Low** | **Flare** | Win | Frequency | 4 | 4 |  | 0.627 (**) | 0.167 |
|  |  |  | Percentage | 50.0% | 50.0% |  |  |  |
|  |  | Lose | Frequency | 2 | 4 |  |  |  |
|  |  |  | Percentage | 33.3% | 66.7% |  |  |  |
| **Middle** | **Flare** | Win | Frequency | 18 | 35 | 4.116* | 0.042 | -0.210 |
|  |  |  | Percentage | 34.0% | 66.0% |  |  |  |
|  |  | Lose | Frequency | 22 | 18 |  |  |  |
|  |  |  | Percentage | 55.0% | 45.0% |  |  |  |
| **High** | **Flare** | Win | Frequency | 25 | 26 | 0.892 | 0.345 | 0.095 |
|  |  |  | Percentage | 49.0% | 51.0% |  |  |  |
|  |  | Lose | Frequency | 19 | 29 |  |  |  |
|  |  |  | Percentage | 39.6% | 60.4% |  |  |  |

Low, low-point classification; Middle, middle-point classification; High, high-point classification; Back, plays where the screener was on the end-line side of the defense who protected the user; Cross, plays where the screener was on the middle-line (the imaginary line connecting baskets running through the center of the court) side of the defense who protected the user; Down, plays where the screener on the center-line side of the defense who protected the user; Flare, plays where the screener on the side-line side of the defense who protected the user.

*: *p* < 0.05.

**We adopted *p*-value by Fisher's method.
